# Comparison of transcriptional responses between pathogenic and nonpathogenic hantavirus infections in Syrian hamsters using NanoString

**DOI:** 10.1371/journal.pntd.0009592

**Published:** 2021-08-02

**Authors:** Rebecca L. Brocato, Louis A. Altamura, Brian D. Carey, Casey C. Perley, Candace D. Blancett, Timothy D. Minogue, Jay W. Hooper

**Affiliations:** 1 Virology Division, United States Army Medical Research Institute of Infectious Diseases, Fort Detrick, Maryland, United States of America; 2 Diagnostic Systems Division, United States Army Medical Research Institute of Infectious Diseases, Fort Detrick, Maryland, United States of America; Instituto Evandro Chagas, BRAZIL

## Abstract

**Background:**

Syrian hamsters infected with Andes virus (ANDV) develop a disease that recapitulates many of the salient features of human hantavirus pulmonary syndrome (HPS), including lethality. Infection of hamsters with Hantaan virus (HTNV) results in an asymptomatic, disseminated infection. In order to explore this dichotomy, we examined the transcriptome of ANDV- and HTNV-infected hamsters.

**Results:**

Using NanoString technology, we examined kinetic transcriptional responses in whole blood collected from ANDV- and HTNV-infected hamsters. Of the 770 genes analyzed, key differences were noted in the kinetics of type I interferon sensing and signaling responses, complement activation, and apoptosis pathways between ANDV- and HTNV-infected hamsters.

**Conclusions:**

Delayed activation of type I interferon responses in ANDV-infected hamsters represents a potential mechanism that ANDV uses to subvert host immune responses and enhance disease. This is the first genome-wide analysis of hantavirus-infected hamsters and provides insight into potential avenues for therapeutics to hantavirus disease.

## Author summary

Hantaviruses co-evolved in specific animal hosts (e.g. rodents) where they persistently infect endothelial cells without causing disease. When these zoonotic viruses cross into humans, usually by inhalation, ingestion, or bite, the endothelium becomes infected, and this infection leads to severe and often lethal disease. Attempts to develop animal models for hantavirus disease have only met with partial success. Syrian hamsters infected with Andes virus (ANDV), a New World hantavirus, develop a disease that closely mimics hantavirus pulmonary syndrome (HPS) in humans. Old World hantaviruses, such as Hantaan virus (HTNV), readily infect Syrian hamsters but do not cause disease. We were interested in understanding why both ANDV and HTNV can readily infect hamsters, but only ANDV causes disease. To investigate this, Syrian hamsters were infected with ANDV or HTNV and a transcriptomics (i.e., NanoString) approach was used to evaluate the animals’ responses to exposure. We identified key differences in gene activity. For example, HTNV exposure triggered early activity in the interferon pathway, whereas the interferon response was delayed following ANDV exposure. This difference in host response will be the focus of future studies aimed at understanding hantavirus pathogenesis and developing HFRS animal models. This Syrian hamster NanoString codset can be used for studying pathogenesis in other disease models, such as SARS-CoV-2.

## Introduction

Hantavirus disease encompasses two syndromes, hantavirus pulmonary syndrome (HPS) caused by New World hantaviruses, and hemorrhagic fever with renal syndrome (HFRS) caused by Old World hantaviruses [1]. Hantaviruses predominantly infect microvascular endothelial cells, resulting in vascular leakage as a result of endothelium dysfunction [2]. In the case of HPS, this endothelial dysfunction occurs primarily in the lung whereas in HFRS, this occurs primarily in the kidney [1]. Both HPS- and HFRS-causing hantaviruses share a similar incubation period and infection manifests with many shared clinical signs [3–8]. There is also dissemination and pathology beyond the target organ. For example, in severe cases of HPS, kidney failure is also evident [4,9,10].

The Syrian hamster has become the animal model of choice for the testing of medical countermeasures against Andes virus (ANDV), a New World hantavirus, given that infection of these animals results in a disease with similarities to human HPS [11]. The ANDV hamster disease model is characterized by severe dyspnea, pulmonary edema, and fluid in the pleural cavity [11]. In contrast, Syrian hamsters infected with Old World hantaviruses, such as Hantaan virus (HTNV), results in an asymptomatic, disseminated infection [12]. While limited for pathogenesis studies due to the lack of disease, these models have shown utility for the testing of vaccines and neutralizing antibody efficacy by passive transfer [13]. Efforts have been made to develop a HTNV disease animal model in many species, namely mice, rabbits, ferrets, and nonhuman primates, but none have recapitulated the renal failure characteristic of severe human HFRS cases [14]. A single study has shown hemorrhage in the renal medulla of mice exposed to a highly passaged HTNV strain [15], with this being the closest recapitulation of human HFRS disease yet.

The Syrian hamster is used as a model system for not only hantaviruses but has emerged as a model system for other infectious diseases, namely COVID-19 [16–19]. Hamster reagents are becoming more readily available allowing for increased in-depth analysis of mechanisms of pathogenesis that have previously not been possible. For this study, we developed a panel containing hamster-specific genes to evaluate the immune response to hantavirus infection. NanoString’s nCounter platform uses direct hybridization-based digital counting technology to monitor transcriptome profiles. Using this hamster immune response panel, we were able to tease apart host response pathways activated in the pathogenic ANDV/hamster model when compared to the nonpathogenic HTNV/hamster model, or vice versa. This has implications for antiviral targets for hantavirus disease and the continued effort to develop a HTNV disease model.

## Materials and methods

### Ethics statement

Animal research was conducted under an IACUC approved protocol at USAMRIID (USDA Registration Number 51-F-00211728 & OLAW Assurance Number A3473-01) in compliance with the Animal Welfare Act and other federal statutes and regulations related to animals and experiments involving animals. The facility where this research was conducted is fully accredited by the Association for Assessment and Accreditation of Laboratory Animal Care, International and adheres to principles stated in the Guide for the Care and Use of Laboratory Animals, National Research Council, 2011.

### Study design

This serial sampling study consisted of 60 total animals split into 3 groups receiving either PBS, ANDV, or HTNV. Three animals per time point per virus were euthanized at 6 hpi, 2, 4, 6, 8, 10 and 12 dpi. In the HTNV challenge group, additional groups of 3 animals each were euthanized on 14, 17, 23 and 35 days post-infection. To compare gene expression between the two challenge viruses, data from HTNV days 14–35 was not included in the analysis.

### Viruses

ANDV strain Chile-9717869 (Genbank IDs: AF291702, AF291703, AF291704) [11] and HTNV strain 76–118 (Genbank IDs: KT885049.1, KT885048.1, KT885047.1) [20], both isolated from their natural reservoir, were twice plaque purified and propagated in Vero E6 cells (Vero C1008; ATCC CRL 1586). For the described studies, ANDV passage 2 (p2) and HTNV p3 were used. Vero E6 cells were maintained in completed Eagle’s minimal essential medium with Earle’s salts (EMEM) containing 10% fetal bovine serum (FBS), 10 mM HEPES pH 7.4, and antibiotics (penicillin [100 U/mL], streptomycin [100 μg/mL]) (cEMEM) at 37°C in a 50% CO_2_ incubator.

### Hamster infections

Female Syrian hamsters, aged 6–8 weeks (Envigo) were anesthetized by inhalation of vaporized isoflurane (3%) using an IMPAC 6 veterinary anesthesia machine. Once anesthetized, hamsters were injected with 2,000 plaque forming units (PFU) of ANDV or HTNV diluted in phosphate-buffered saline (PBS). Intramuscular (caudal thigh) injections consisted of 0.2 mL delivered using a 1-mL syringe with a 25-gauge, 5/8-inch needle. Vena cava blood collection occurred under either isoflurane or injectable anesthesia (0.1 mL/100g of body weight of ketamine-acepromazine-zylazine mixture), and were limited to 7% of total blood volume per week. Terminal blood collection was performed by cardiac puncture at the time of euthanasia. Euthanasia was performed on animals meeting early endpoint criteria. All work involving animals was performed in an animal biosafety level 4 (ABSL-4) laboratory.

### Isolation of RNA and real-time PCR

Approximately 250 mg of frozen lung or kidney tissue was homogenized in 1.0 mL TRIzol reagent using gentleMACS M tubes and a gentleMACS dissociator (Miltenyi Biotec) on the RNA setting. Serum samples were added directly to TRIzol reagent. Whole blood samples were added directly to TRIzol LS reagent. RNA was extracted using Qiagen miRNeasy Mini kit per the manufacturer’s instructions. The concentration of the extracted RNA was determined using a NanoDrop 8000 instrument and standardized to a final concentration of 10 ng/μl. Real-time PCR was conducted on a BioRad CFX thermal cycler using an Agilent Brilliant II QRT-PCR one-step master mix. ANDV primer/probe sequences are described in [21]. HTNV forward primer 5’- GCT GGC GTA TAG CCT TTG AC-3’, reverse primer 5’- AGT TAG CCT CCT TGG TGG TC-3’ and probe 5’- /56-FAM/ATG CCA CTT /ZEN/ GCC GCT GCC GT /3IABkFQ/ -3’. Cycling conditions were 30 min at 48°C, 10 min at 95°C, followed by 40 cycles of 15 s at 95°C and 1 min at 60°C. Data acquisition occurs following the annealing step.

### N-ELISA

Recombinant N protein ELISA were performed on serum samples as previously described [22]. Samples were gamma-irradiated (on dry ice) with 3 x 10^6^ rads from a ^60^C source.

### NanoString

Total RNA samples from whole blood were analyzed using a custom-designed NanoString nCounter gene expression codeset targeting 770 hamster genes for targeted transcriptomics. For each gene, a single probeset was designed (**S3 Table**). Probeset-target RNA hybridization reactions were performed according to the manufacturer’s protocol. For each hybridization reaction, 100 ng total RNA was used. Purified probeset-target RNA complexes from each reaction were processed and immobilized on nCounter Cartridges using an nCounter MAX Prep Station and transcripts were quantified on the Digital Analyzer (GEN 2).

### Analysis methods

NanoString output files by timepoint were compiled using R [23]. Heatmaps were generated using Morpheus with Euclidian distance for hierarchical clustering [24] and then graphed in Prism. Venn diagrams were generated using Venny [25]. All other graphs generated using GraphPad Prism 8. Ingenuity Pathway Analysis (QIAGEN) was used to analyze genes that are significantly differentially expressed for pathway enrichment. Overlap graphs were generated by IPA using the top 25 pathways and heatmaps showing pathways that affect numerous diseases and functions were also generated.

## Results

### Hantavirus infection in Syrian hamsters

**Fig 1:**
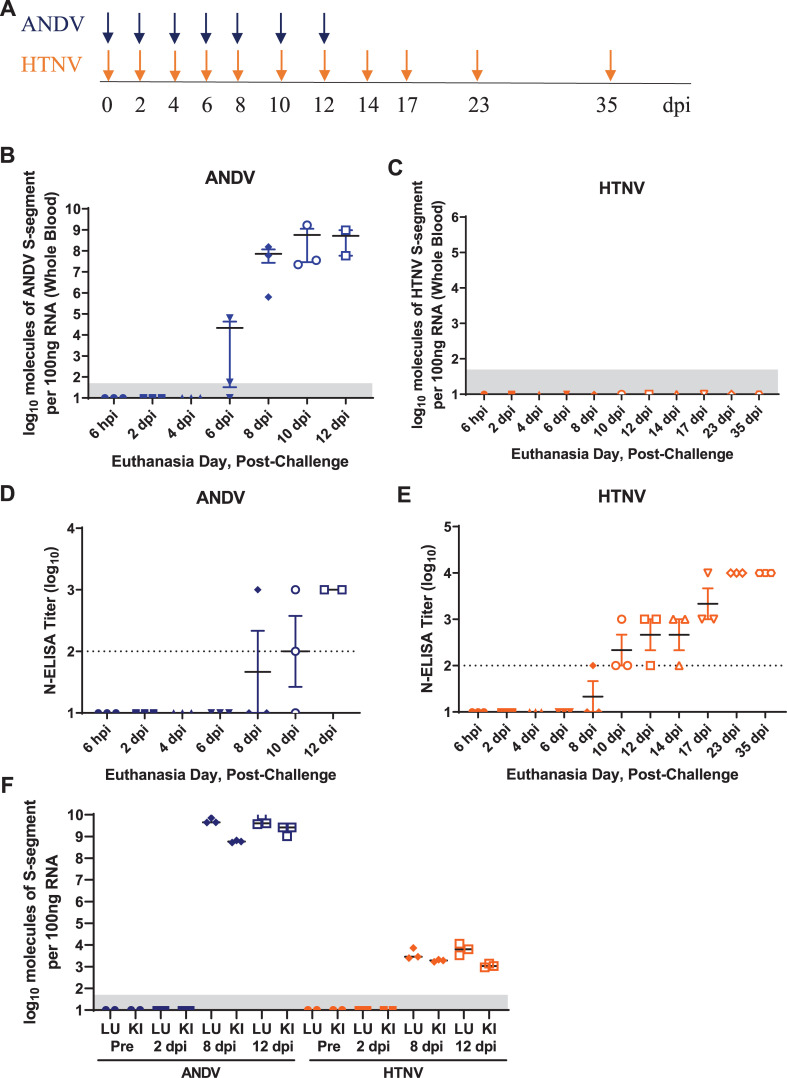
Hantavirus Infections in Syrian Hamsters. **A)** Groups of 3 hamsters each were exposed to either 2,000 PFU ANDV or HTNV and euthanized on the indicated dpi (ANDV, blue arrows; HTNV, orange arrows). RNA from whole blood from **B)** ANDV- and **C)** HTNV-infected hamsters was analyzed for viral RNA by RT-PCR (limit of detection is 50 copies, shaded area). N-ELISA endpoint titers (log_10_) were conducted with serum collected from **D)** ANDV- and **E)** HTNV-infected hamsters at the time of euthanasia. **F)** ANDV and HTNV viral RNA was detected by RT-PCR in homogenized lung (LU) and kidney (KI) tissue collected at the time of euthanasia (limit of detection is 50 copies, shaded area).

In an effort to identify host response signatures of pathogenic and nonpathogenic hantaviruses to differentiate between hantavirus disease syndromes, we characterized host expression kinetics in whole blood from Syrian hamsters infected with 2,000 PFU of ANDV or HTNV by the intramuscular route. A serial euthanasia experiment was conducted in which 3 animals per timepoint (**Fig 1A**) were deeply anesthetized, whole blood collected, euthanized and tissues collected. Confirming a previous report [26], viremia is detectable in whole blood RNA from ANDV-infected hamsters beginning on 6 days post-infection (dpi) (**Fig 1B**). In contrast, viremia was not detected in whole blood RNA from HTNV-infected hamsters (**Fig 1C**). A nucleocapsid ELISA (N-ELISA) was performed from serum demonstrating that hamsters had seroconverted to ANDV and HTNV infection by 12 and 10 dpi, respectively (**Fig 1D and 1E**). RNA from lungs and kidneys was isolated from a subset of hamsters and analyzed using ANDV and HTNV RT-PCR assays. Viral genome was present in both lungs and kidneys on 8 and 12 dpi following ANDV or HTNV challenge (**Fig 1F**).

### Development of a hamster NanoString codeset and pathway analysis

**Fig 2:**
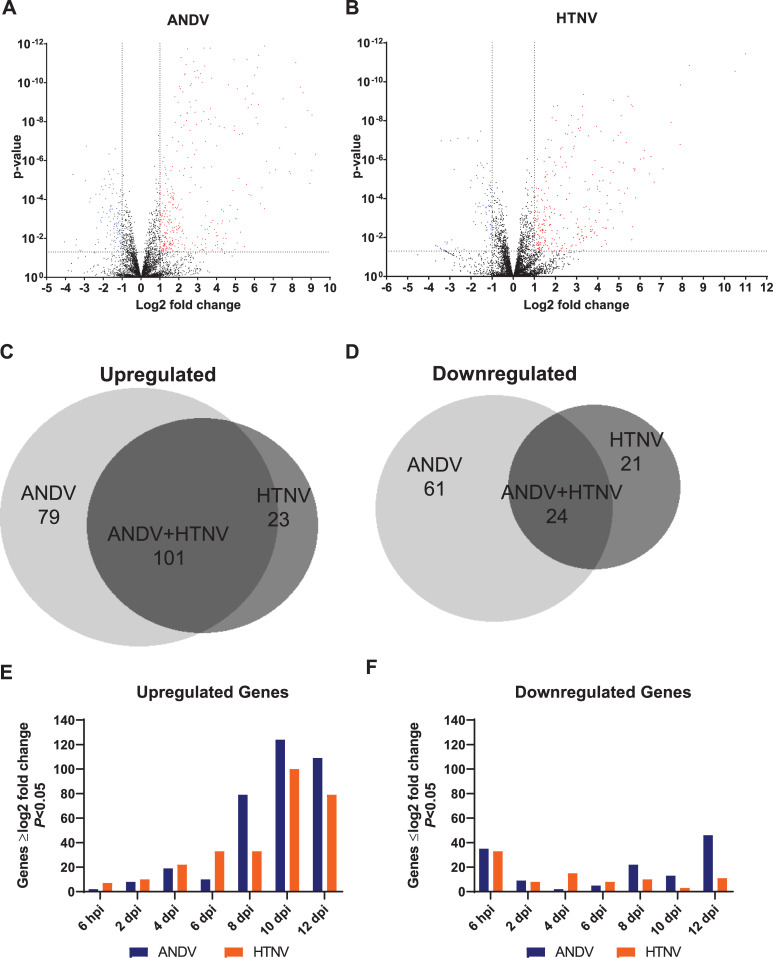
Overview of NanoString Gene Expression. Summary of gene expression from ANDV and HTNV time points 6 hpi, 2, 4, 6, 8, 10, and 12 dpi shown as **A, B)** volcano plots with significant genes that are > log2 fold change (red symbols) and < log2 fold change (blue symbols). **C, D)** Venn diagrams indicating significant genes upregulated and downregulated by ANDV, HTNV, and ANDV + HTNV. **E, F)** Upregulated and downregulated significant genes by time point.

Using published sequences of hamster genes, a NanoString hamster panel was developed targeting 770 Syrian hamster genes. These include housekeeping genes, immunology and inflammation response pathway genes, and genes associated with vascular function and coagulation pathways. *P* values were calculated and volcano plots shown depicting significant genes that are less than -1 or greater than 1 log2 fold change (**Fig 2A and 2B**).

A comparison of total up- and downregulated genes (genes with ≥log2 fold change and *P*<0.05) was compiled from 6 hours post-infection (hpi) to 12 dpi of ANDV and HTNV disease course in hamsters (**Fig 2C–2F**). The increase in upregulated genes in ANDV-infected hamsters corresponds with the increase in viremia, detectable in all animals at 8 dpi (**Fig 1B**). Increases in upregulated genes from HTNV-infected hamsters is detectable prior to antibodies to HTNV nucleocapsid (**Fig 1E**) or viremia (**Fig 1C**). The total number of downregulated genes is similar for both ANDV- and HTNV-infected animals. There were 5 genes that were not detected in the hamster NanoString panel. Four of these genes were undetected in the ANDV samples (*KCNE1*, *TNFSF15*, *IL20* and *PMCH*) and a single gene was undetected in the HTNV samples (*CCL19*).

**Fig 3:**
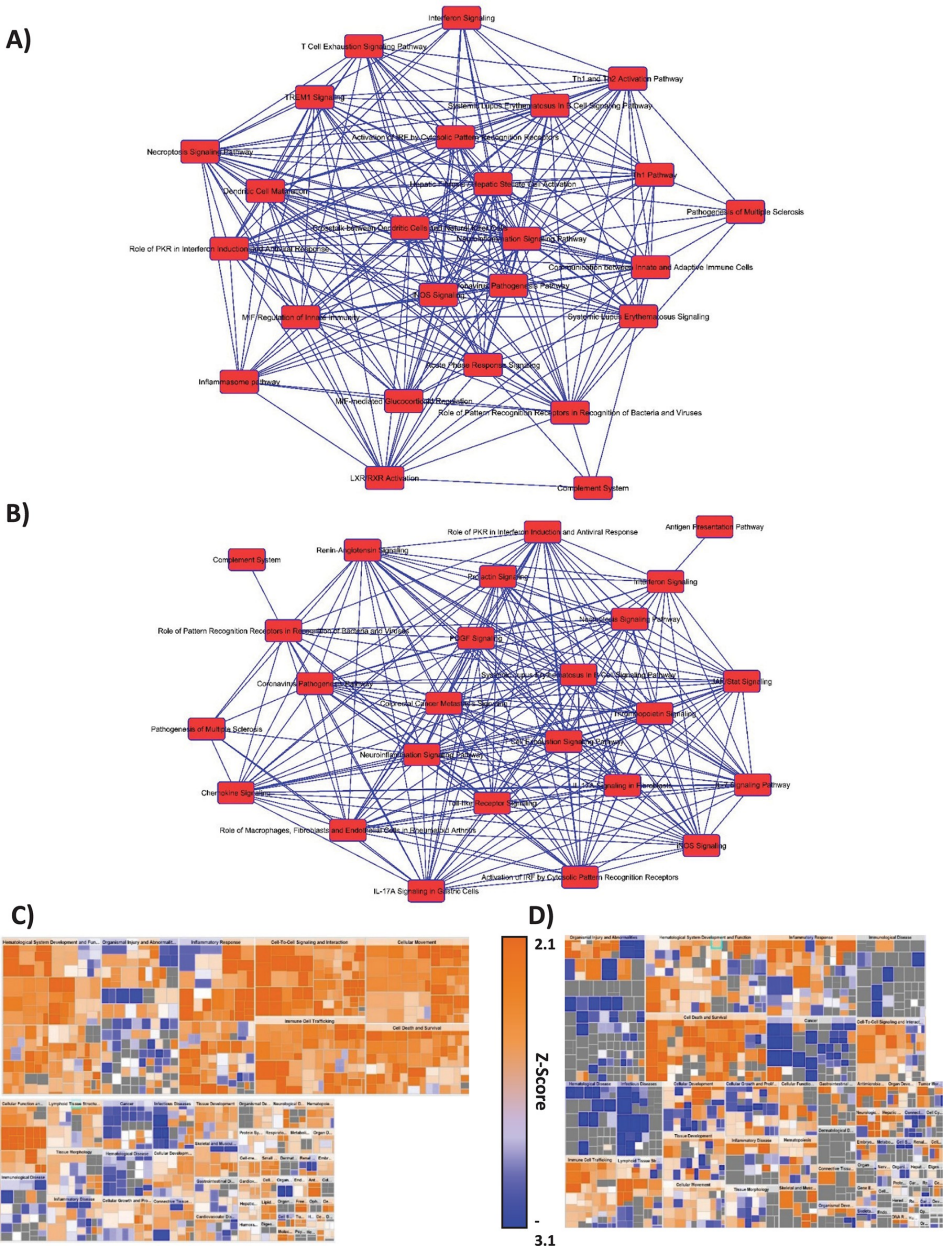
Ingenuity Pathway Analysis of Differentially Expression Genes in ANDV and HTNV Infected Hamsters. Pathway analysis was completed using gene expression data collected from ANDV and HTNV infected hamsters at 10 dpi. Overlap graphs showing relationship between the 25 most significantly enriched pathways in **A)** ANDV and **B)** HTNV infected hamsters. Blue lines indicate at least one shared gene by connected pathways. Tables showing these pathways are listed in **S1 and S2 Tables**. **C, D)** Heatmap of physiological functions effected by identified pathways for ANDV and HTNV infected hamsters. Functions (large boxes) sized by p-value where larger boxes indicate larger -log(p-value) and pathways (small boxes) are colored by z-score with orange indicating upregulated pathways and blue indicating a downregulated pathway. Gray boxes indicate statistically significant pathway enrichment with no discernable direction of regulation. Pathway boxes are also sized by -log(p-value).

Pathway analysis was performed on differentially expressed genes from 10 dpi ANDV and HTNV hamster whole blood samples using Ingenuity Pathway Analysis (IPA). This timepoint was selected as 10 dpi is the peak of ANDV viremia (**Fig 1B**) and the first timepoint where 3/3 HTNV-exposed animals had serocoverted (**Fig 1E**). Overlap graphs depict the relationships between genes in the top 25 most significantly enriched pathways at this single timepoint in ANDV (**Fig 3A and S1 Table**) and HTNV (**Fig 3B and S2 Table**). Heatmaps of physiologic functions, sized by significance, indicate a greater number of upregulated pathways in ANDV infection when compared to HTNV infection (**Fig 3C and 3D**). Highly enriched pathways are discussed below.

### Interferon signaling pathways

**Fig 4:**
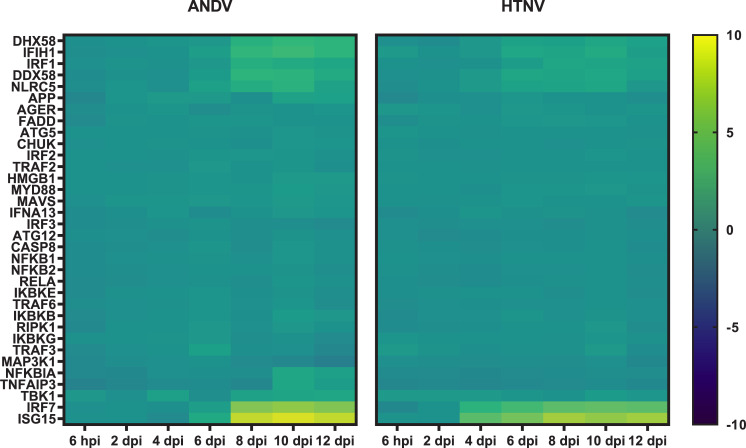
Interferon Sensing and Induction in Hantavirus Infected Hamsters. Genes associated with interferon sensing and induction are displayed as a heat map with hierarchical clustering and intensity of the colors corresponding to the magnitude of fold change over the baseline.

Several interferon signaling pathways were identified by IPA analysis such as Role of JAK1, JAK2, and TYK in Interferon signaling, Role of PKR in Interferon induction and Antiviral response and canonical Interferon signaling pathway. Slightly increased expression (1–2 log2 fold) of the retinoic acid-inducible gene I (RIG-I)-like receptors (RLRs), RIG-I (*DDX58*), melanoma differentiation associated factor 5 (MDA5, *IFIH1*), and laboratory of genetics and physiology 2 (LGP2, *DHX58*) are observed in HTNV-infected hamsters on 4 dpi whereas this level of RLR expression was not observed in ANDV-infected hamsters until 8 dpi (**Fig 4**). Surprisingly, the gene encoding the adaptor protein IPS-1 (also known as *MAVS*, *VISA*, or *Cardif*) was not upregulated at any timepoint during either HTNV or ANDV infection. The transcription factor interferon regulatory factor 7 (*IRF7*), but not *IRF3* nor NF-κB (*NFKB1* and *NFKB2*), had increased expression in both HTNV- and ANDV-infected hamsters (beginning on 4 and 8 dpi, respectively). Viral recognition can also occur through Toll-like receptor (TLR) signaling (**S1 Fig**). However, there is not increased expression of *TLR3* or *TLR7*, nor is *MyD88* (innate immune signal transduction adaptor).

**Fig 5:**
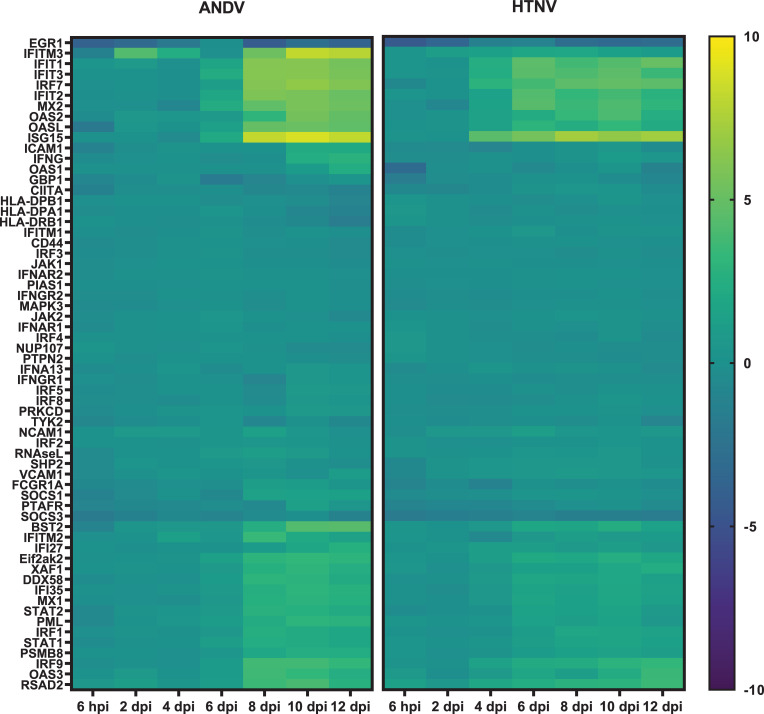
Interferon Signaling in Hantavirus Infected Hamsters. Genes associated with interferon signaling are displayed as a heat map with hierarchical clustering and intensity of the colors corresponding to the magnitude of fold change over the baseline.

Expressed IFN-α and IFN-β act in an autocrine and paracrine manner by binding IFNAR. Yet the NanoString analysis does not indicate increased expression of *IFNAR1*, *IFNAR2*, or the JAK kinases *TyK2* and *JAK1* at any timepoint (**Fig 5**). However, there is increased expression of *STAT1*, *STAT2*, and *IRF9* that form a complex known as ISGF3 [27]. ISGF3 is a transcriptional activator that binds to the IFN stimulated response element to activate the transcription of interferon stimulated genes (ISGs). Genes in several of these IFN-mediated effector pathways have increased expression in the NanoString analysis for both ANDV and HTNV. These include *ISG15* in the ISG15 ubiquitin-like pathway, *Mx1* and *Mx2* from the Mx GTPase pathway, *OAS2*, *OAS3*, and *OASL* from the 2’-5’ oligoadenlate synthetase-directed ribonuclease L (*RNaseL*) pathway, and *EIF2αk2* from the protein kinase R pathway. Additionally, the ISGs *IFIT1*, *IFIT2*, and *IFIT3* that can inhibit viral replication have increased expression.

Activation of genes in these pathways is similar comparing hamsters infected with HTNV and ANDV. The main difference lies in the kinetics of activation. Overall, we begin to observe genes associated with these interferon pathways activated 4 dpi following HTNV infection and 6 dpi following ANDV infection. Another key difference between HTNV and ANDV infected hamsters in the increased expression of *IFITM3* that is only observed at low levels in HTNV-infected hamsters yet is markedly upregulated at 2 dpi ANDV.

### Cytokine related pathways

**Fig 6:**
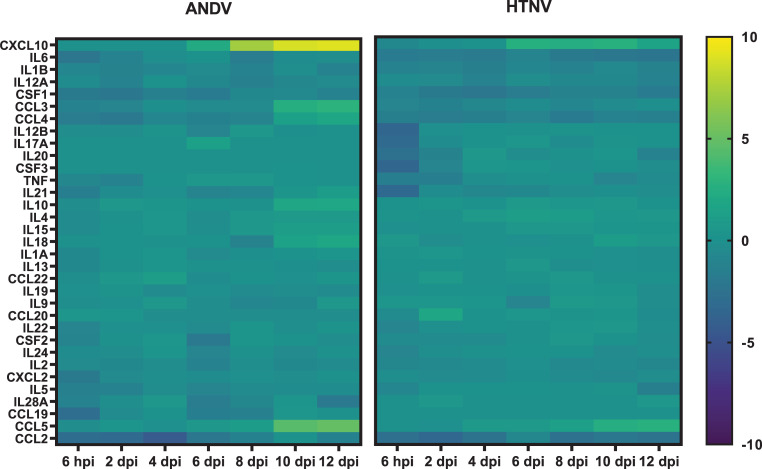
Cytokine/Chemokine Expression in Hantavirus Infected Hamsters. Genes associated with cytokine and chemokine expression are displayed as a heat map with hierarchical clustering and intensity of the colors corresponding to the magnitude of fold change over the baseline.

Numerous cytokine related pathways were also identified to be significantly enriched such as Role of JAK family kinases in IL-6 type cytokine signaling, Role of JAK1 and JAK3 in γc Cytokine Signaling, and Differential Regulation of Cytokine Production in Intestinal Epithelial Cells by IL-17A and IL-17F. In contrast to the early activation of type I IFN genes early in HTNV infection (*i*.*e*. 4 dpi) and later in ANDV infection (*i*.*e*. 6 dpi), there is a lack of cytokine gene expression at comparable timepoints. However, coinciding with the onset of viremia in ANDV-infected hamsters, there is an upregulation of the *CXCL10* gene encoding the IP-10 protein (**Fig 6**).

Of note, there is a subset of chemokine and cytokine genes that are markedly (>2-log2 fold) downregulated initially after HTNV infection. Six hours after hamsters were infected with HTNV, the genes *IL12B* (-3.33 log2 fold), *IL21* (-3.29 log2 fold), *IL17A* (-3.2 log2 fold), *CCL2* (-2.78 log2 fold), *CSF3* (-3.45 log2 fold), and *IL20* (-2.55 log2 fold) were downregulated, with no increase or decrease in expression observed 2 dpi, except in the case of *CCL2*. *CCL2* remains downregulated (>2 log2 fold) early in both HTNV and ANDV infection (6 hpi to 4 dpi) and remained downregulated (>2 log2 fold) from 8 dpi through the remainder of the tested timepoints.

### Complement activation

**Fig 7:**
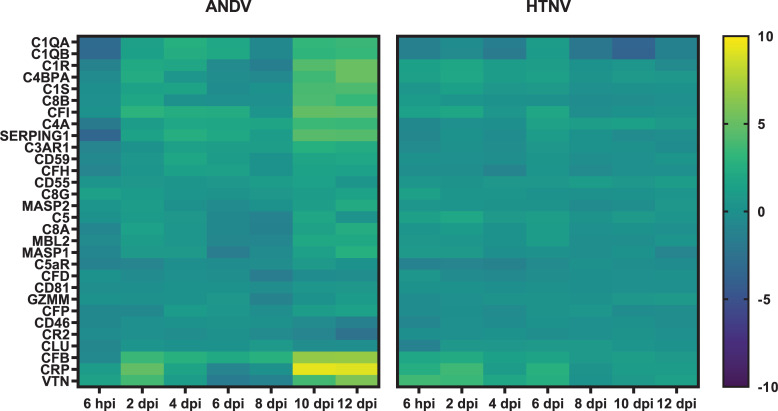
Complement Pathway Expression in Hantavirus Infected Hamsters. Genes associated with complement expression are displayed as a heat map with hierarchical clustering and intensity of the colors corresponding to the magnitude of fold change over the baseline.

Genes associated with complement pathways have increased expression in both HTNV and ANDV hamster models (**Fig 7**). As early as 6 hpi following HTNV challenge, upregulation of *VTN* (3.66 log2 fold) and *CRP* (2.43 log2 fold) was observed. Correspondingly, at 6 hpi following ANDV challenge, a downregulation of *SERPING1* (-3.42 log2 fold), *C1QA* (-3.17 log2 fold), and *C1QB* (-3.24 log2 fold) was observed. However, starting 2 dpi following ANDV challenge, expression of the previously downregulated genes returns to baseline and there is increased expression similar to what is exhibited in HTNV infection including *VTN* (3.69 log2 fold) and *CRP* (4.8 log2 fold), including *CFB* (3.43 log2 fold) and *CFI* (2.82 log2 fold). Robust expression of several classical complement pathway genes are upregulated at 10 and 12 dpi ANDV challenge, corresponding with maximum viremia. These genes include *C4BPA*, *C1R*, *C1S*, *C4A*, *C1QA*, *C1QB*, and *CRP* (all 3–9 log2 fold). Additionally, *CFB*, associated with the alternative complement pathway, *C8B*, a component of the membrane attack complex, and *CFI* and *SERPING1*, complement cascade regulators, were also upregulated. Compared to hamsters infected with HTNV, ANDV-infected hamsters had a marked increase in overall gene expression in this pathway.

### Coagulation pathways

**Fig 8:**
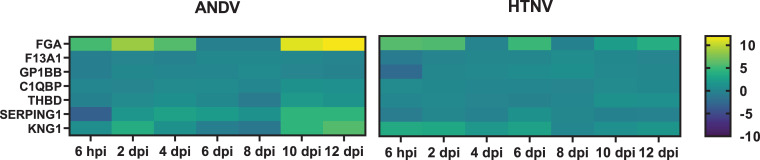
Coagulation Pathway Expression in Hantavirus Infected Hamsters. Genes associated with coagulation pathway expression are displayed as a heat map with hierarchical clustering and intensity of the colors corresponding to the magnitude of fold change over the baseline.

Pathways involved in coagulation including fibrin deposition and Intrinsic Prothombin Activation Pathway were also identified in IPA analysis to be significantly enriched. Increase in genes associated with intrinsic and common coagulation pathways, namely *KNG1* and *FGA*, are upregulated early in both HTNV and ANDV infection (6 hpi to 2 dpi) and peak expression occurs coinciding with ANDV viremia (*FGA* >11 log2 fold on 10 and 12 dpi, *KNG1* >4 log2 fold on 10 and 12 dpi) (**Fig 8**).

### Apoptosis pathways

**Fig 9:**
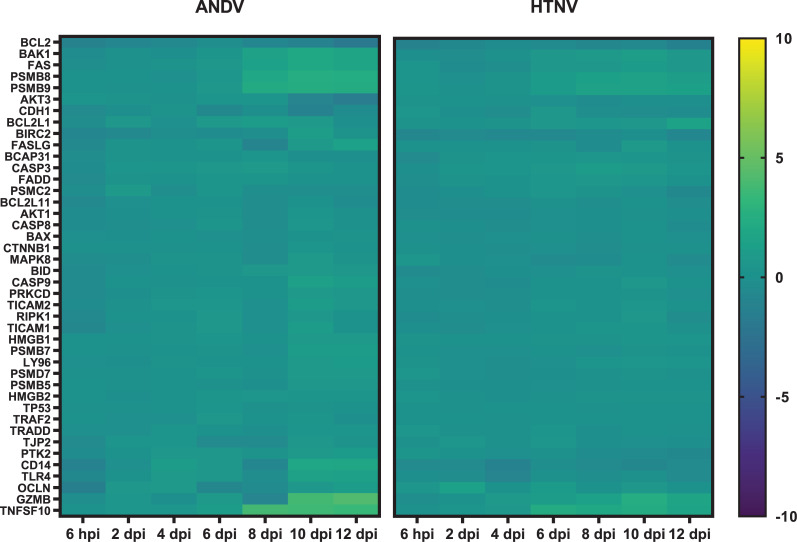
Apoptosis Pathway Expression in Hantavirus Infected Hamsters. Genes associated with apoptosis pathway expression are displayed as a heat map with hierarchical clustering and intensity of the colors corresponding to the magnitude of fold change over the baseline.

Apoptosis pathways are significantly enriched as well including Retinoic acid mediated apoptosis signaling, Calcium induced T lymphocyte apoptosis, Induction of Apoptosis by HIV-1, and canonical Apoptosis signaling. There is early, increased expression of *TNFSF10* (or TRAIL) beginning on 6 dpi in HTNV-infected hamsters (2.23 log2 fold) that continues on 8 dpi (1.99 log2 fold) and 10 dpi (2.22 log2 fold) (**Fig 9**). In ANDV-infected hamsters, increased *TNFSF10* expression is first observed on 8 dpi and is more robust than what is observed in HTNV-infected animals (3.78 log2 fold). Increased *TNFSF10* expression continues in ANDV-infected hamsters through 10 dpi (3.62 log2 fold) and 12 dpi (3.37 log2 fold). A similar expression pattern is observed for Granzyme B (*GZMB*), with modest expression on 10 dpi of HTNV-infected hamsters (2.47 log2 fold), compared to ANDV-infected hamsters (10 dpi, 3.69 log2 fold; 12 dpi, 4.14 log2 fold).

There is increased expression of both *FAS* and *BAK1* (1.98 log2 fold and 1.96 log2 fold on 10 dpi) in ANDV-infected hamsters. However, there is a lack of expression of other genes associated with downstream signaling in apoptosis pathways.

### Cell populations

**Fig 10:**
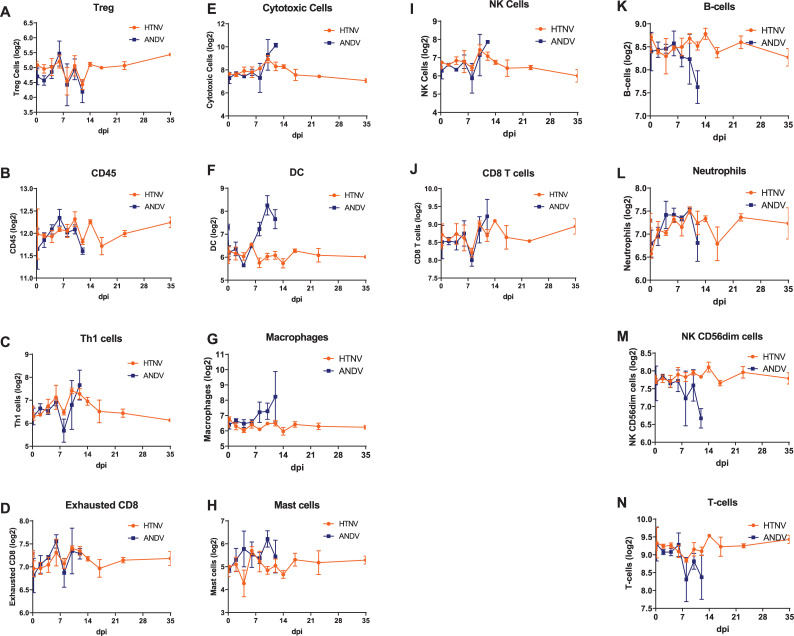
Immune Cell Counts from Hantavirus Infected Hamsters. Genes typically expressed by immune cells are used by NanoString to determine immune cell counts. These cell populations include **A)** Tregs, **B)** CD45 cells, **C)** Th1 cells, **D)** exhausted CD8 cells, **E)** cytotoxic cells, **F)** DCs, **G)** macrophages, **H)** mast cells, **I)** NK cells, **J)** CD8 T cells, **K)** B cells, **L)** neutrophils, **M)** NK CD56dim cells, and **N)** T cells.

The NanoString platform uses genes typically expressed by specific cell types to determine counts of immune cells. Overall, in most cell types analyzed, there are similar patterns through 6 dpi. There is a trend of similar counts between ANDV- and HTNV-infected hamsters through 12 dpi evident in T-regs, CD45 positive cells, Th1 cells, and exhausted CD8 cells throughout the overlapping course of infection (**Fig 10A–10D**). However starting on 8 dpi following ANDV infection, there is an increase in cytotoxic T-cells, dendritic cells, macrophages, mast cells, and to a lesser extent, NK cells and CD8 T-cells when compared to HTNV-infected hamsters (**Fig 10E–10J**). Alternatively, there is a decrease in B-cells, neutrophils, NK CD56dim cells, and T-cells in ANDV-infected hamsters after 8 dpi when compared to HTNV-infected hamsters (**Fig 10K–10N**).

## Discussion

Investigations of the pathogenesis of hantavirus infection is largely limited to human hantavirus disease [7,28,29], nonhuman primate models [30,31] and the ANDV/hamster model [11,26,32] that recapitulate features of human hantavirus disease (HPS). To date, there are no HTNV/nonhuman primate or small animal models that recapitulate the features of human HFRS. We examine the transcriptome of hamsters infected with either ANDV or HTNV and identify some key differences in host immune responses.

The delay in ANDV-induced type I IFN kinetics when compared to HTNV infection in hamsters is the most striking disparity between these two viruses. Early signaling through the RLR pathway of HTNV-infected mice contributes to control of the virus [33]. Cimica, *et al*. noted that the ANDV N protein restricts induction of IFN, namely IFN-β and downstream ISGs, beginning with the inhibition of the RLRs, RIG-I, and MDA5 in cell culture [34]. Similarly, a comparison of glycoprotein, Gn, of pathogenic and nonpathogenic hantaviruses demonstrated that the pathogenic hantavirus NY-1 inhibited cellular IFN responses [35]. Neither of these studies directly compared ANDV with HTNV gene segments, however, we can hypothesize that virulence determinants encoded by ANDV may contribute to the delay in IFN induction observed in the transcriptome of ANDV-infected hamsters.

Of interesting note is two genes, *BST2* and *IFITM3*, which are upregulated in ANDV-infected hamsters on 8 dpi but not in HTNV-infected hamsters. *BST2* is upregulated by type I IFN signaling and plays a dual role of inhibiting *DDX58*-mediated type I IFN signaling through a negative-feedback loop while tethering virus particles in infected cells [36]. Regulated by *IRF1*, *BST2* expression plays a role in regulating vesicular stomatitis virus infection in human bronchial epithelial cells through type I IFN signaling [37]. We have recently demonstrated that hamsters treated with polyI:C, a potent inducer of type I IFN, protects from a lethal ANDV challenge [38]; however, the exact role of *BST2* in the ANDV/hamster model has yet to be elucidated. *IFITM3*, a restriction factor that prevents endocytosed viral particles from reaching the cellular cytoplasm, has been shown to have an effect on enveloped viruses [39]. *IFITM3*, initially upregulated on 2 dpi ANDV challenge and continued through 8–12 dpi, coinciding with peak viremia, is likely a cellular defense against ANDV infection. However, a recent report notes that overexpression of *IFITM3* has an inhibitory effect on type I IFN signaling acting as a negative-feedback loop [40], similar to *BST2* expression. Taken together, ANDV virulence determinants that specifically inhibit IFN responses, combined with activation of negative-feedback loops that limit IFN induction, represent a possible mechanism of ANDV virulence in Syrian hamsters that is not exhibited in HTNV infection.

The upregulation of *CXCL10* and the expression of IP-10 has been noted *in vitro* [41,42] and in human HPS [43] and HFRS [44] cases. Additionally, Sundstrom *et al*. reported induction of RANTES, encoded by *CCL5* in human endothelial cells [42]. The upregulation of *CXCL10* and *CCL5* in ANDV-infected hamsters corresponds with an increase in macrophages, dendritic cells, CD8 T cells, cytotoxic cells and NK cell counts hypothesized to be recruited to the lung by expressed RANTES and IP-10. Differences in IL-6 were observed between human hantavirus infections and the hamster models. HPS disease severity has been associated with IL-6 expression [45]; however, there was a lack of IL-6 transcription observed in ANDV-infected hamsters. This is an interesting finding as *IL6* and other cytokines upregulated in human hantavirus infections (*i*.*e*. *IL1β*) were not upregulated even at the peak of ANDV viremia.

Surprisingly little research has been done regarding complement activation in hantavirus disease. In human cases of Puumala virus infection, complement activation via the alternative pathway correlates with disease severity [46]. We observe the upregulation of several classical complement pathway genes (*i*.*e*. *CRP*, C1, C4) corresponding with peak ANDV viremia in hamsters, supporting a role of complement activation in the development of HPS that has also been observed in human HPS cases [45].

Endothelial cell permeability associated with hantavirus infection has been studied extensively; the lack of cytotoxicity or vascular injury of hantavirus-infected endothelial cells indicate permeability of endothelial barrier function [47]. A wide range of effector molecules have been suggested as the cause of hantavirus-induced endothelial cell permeability and a review of these is beyond the scope of this study. However, the kallikrein-kinin system has been implicated in ANDV-directed permeability *in vitro* [48]. Our *in vivo* analysis indicates upregulation of *KNG1*, *FGA* and *SERPINE*, genes that play a role in the kallikrein-kinin system and blood coagulation. These genes, upregulated both early and late in HTNV- and ANDV-infected hamsters warrant further investigation.

When examining the various pathways examined in HTNV- and ANDV-infected hamsters’ side-by-side, and examining the total number of upregulated genes, there is an overall increase in activated genes coinciding with the increase in ANDV viremia on 8, 10, and 12 dpi. We hypothesize that the delay in IFN activation allows ANDV to replicate and disseminate in greater magnitude, leaving the host’s immune system at a disadvantage to fight off the viral assault. As a result, increased gene expression on 8 dpi is insufficient to protect the host from lethal disease. On the contrary, less genes were upregulated in HTNV- versus ANDV-infected hamsters. However, the kinetics and magnitude of these genes is sufficient to prevent HTNV disease. This study provides insights into this dichotomy that expand our knowledge base comparing pathogenic and nonpathogenic hantaviruses.

## Supporting information

S1 Fig TLR Signaling in Hantavirus Infected Hamsters. Genes associated with TLR signaling 4 are displayed as a heat map with hierarchical clustering and intensity of the colors corresponding to the 5 magnitude of fold change over the baseline.
(DOCX)

S1 Table 10 dpi ANDV 25 most significantly enriched pathways. (DOCX)

S2 Table 10 dpi HTNV 25 most significantly enriched pathways. (DOCX)

S3 Table NanoString probeset design. (DOCX)
