# Supplementary material for: Comparison of transcriptional responses between pathogenic and nonpathogenic hantavirus infections in Syrian hamsters using NanoString
Source: PLoS Negl Trop Dis. 2021 Aug 2;15(8):e0009592. doi: 10.1371/journal.pntd.0009592 (PMC8360559; doi:10.1371/journal.pntd.0009592)
Supplement: S1 Fig — Genes associated with TLR signaling 4 are displayed as a heat map with hierarchical clustering and intensity of the colors corresponding to the 5 magnitude of fold change over the baseline. (DOCX) [file pntd.0009592.s001.docx]

**
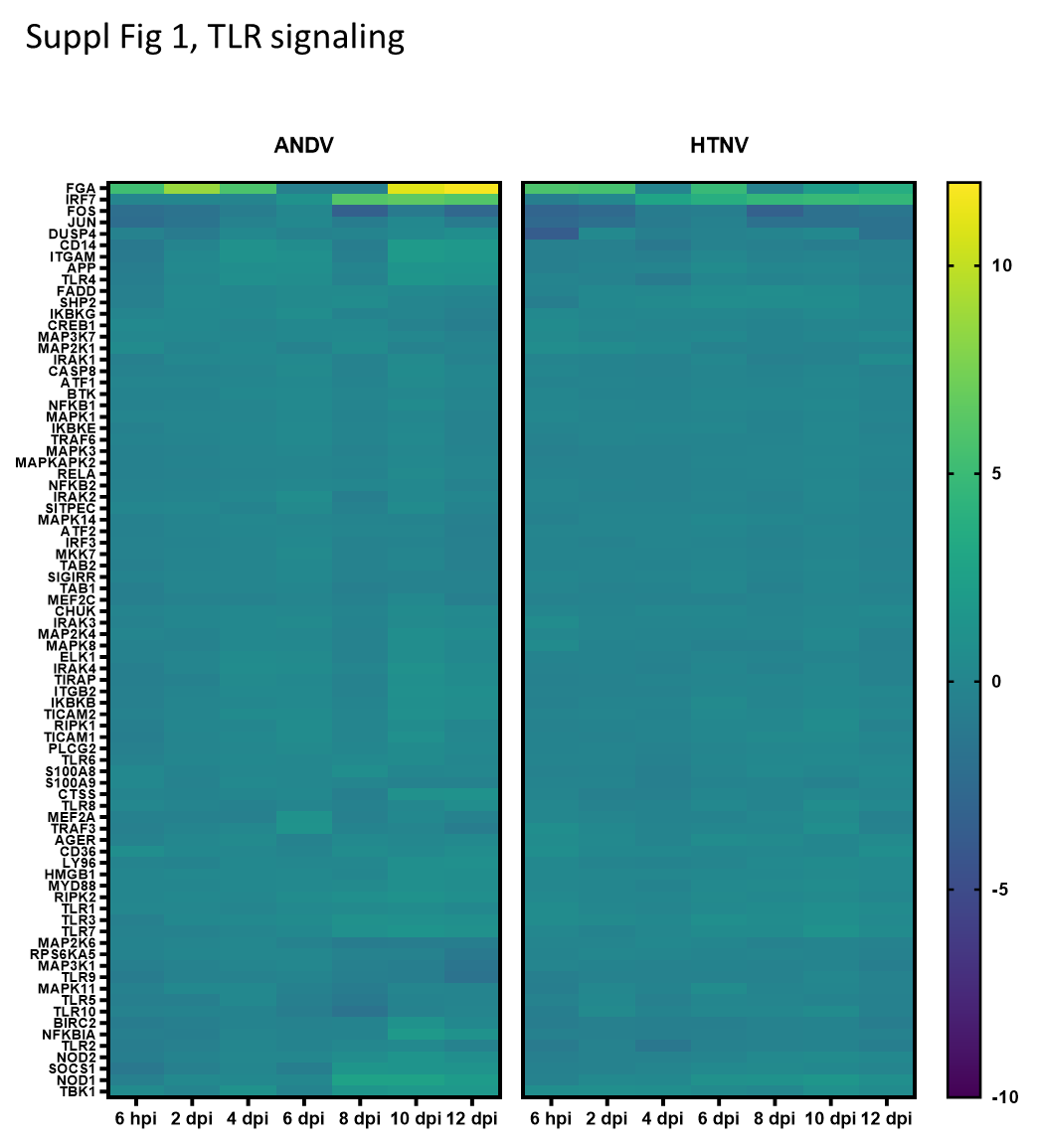
**

**S1 Fig. TLR Signaling in Hantavirus Infected Hamsters.** Genes associated with TLR signaling are displayed as a heat map with hierarchical clustering and intensity of the colors corresponding to the magnitude of fold change over the baseline.
