# Supplementary material for: Comparison of transcriptional responses between pathogenic and nonpathogenic hantavirus infections in Syrian hamsters using NanoString
Source: PLoS Negl Trop Dis. 2021 Aug 2;15(8):e0009592. doi: 10.1371/journal.pntd.0009592 (PMC8360559; doi:10.1371/journal.pntd.0009592)
Supplement: S1 Table — (DOCX) [file pntd.0009592.s002.docx]

S1 Table 1. 10 dpi ANDV 25 most significantly enriched pathways

| **Pathway** | **-log(p-value)** | **Molecules** |
| --- | --- | --- |
| Complement System | 34.9 | C1QA,C1QB,C1QBP,C1R,C1S,C2,C3AR1,C4A/C4B,C4BPA,C5,C6,C8B,C9,CD55,CD59,CFB,CFH,CFI,ITGAM,SERPING1 |
| Interferon Signaling | 21.8 | BAK1,BCL2,IFI35,IFIT1,IFIT3,IFITM2,IFITM3,IRF1,IRF9,ISG15,MX1,PSMB8,STAT1,STAT2 |
| Neuroinflammation Signaling Pathway | 18.3 | APP,BCL2,BIRC2,CASP1,CCL5,CD86,CRP,CXCL10,CXCL12,CYBB,FOS,ICAM1,IRF7,NFE2L2,PLA2G2A,PLA2G4A,PTGS2,PYCARD,STAT1,TBK1,TGFB2,TLR4,TLR7 |
| Role of Pattern Recognition Receptors in Recognition of Bacteria and Viruses | 17.6 | C1QA,C1QB,C3AR1,C5,CASP1,CCL5,CD40LG,DDX58,EIF2AK2,IRF7,NLRC4,NOD2,OAS2,RIPK2,TGFB2,TLR4,TLR7,TNFSF10 |
| Acute Phase Response Signaling | 13.8 | C1R,C1S,C2,C4A/C4B,C4BPA,C5,C9,CFB,CRP,FGA,FOS,HAMP,HP,NFKBIA,SERPING1,TNFRSF1B |
| Role of PKR in Interferon Induction and Antiviral Response | 12.6 | CASP1,DDX58,EIF2AK2,FOS,IRF1,IRF9,MARCO,MSR1,NFKBIA,PYCARD,STAT1,STAT2,TLR4 |
| Coronavirus Pathogenesis Pathway | 11.2 | BCL2,CASP1,CCL5,DDX58,FOS,IRF7,IRF9,NFKBIA,PTGS2,PYCARD,STAT1,STAT2,TBK1 |
| Systemic Lupus Erythematosus In B Cell Signaling Pathway | 11 | BCL2,CCND3,CD40LG,FCGR2B,FOS,IFIT2,IFIT3,IRF7,IRF9,ISG15,STAT1,STAT2,TBK1,TGFB2,TLR7,TNFSF10 |
| Necroptosis Signaling Pathway | 10.9 | BIRC2,CASP1,CYBB,EIF2AK2,IRF9,PLA2G2A,PLA2G4A,PYCARD,STAT1,STAT2,TLR4,TNFRSF1B,TNFSF10 |
| Activation of IRF by Cytosolic Pattern Recognition Receptors | 9.84 | DDX58,IFIT2,IRF7,IRF9,ISG15,NFKBIA,STAT1,STAT2,TBK1 |
| Crosstalk between Dendritic Cells and Natural Killer Cells | 9.81 | CCR7,CD40LG,CD69,CD83,CD86,LTBR,TLR4,TLR7,TNFRSF1B,TNFSF10 |
| LXR/RXR Activation | 9.74 | APOE,C4A/C4B,C9,CD14,FGA,KNG1,MSR1,PTGS2,TLR4,TNFRSF1B,VTN |
| TREM1 Signaling | 9.14 | CASP1,CD83,CD86,FCGR2B,ICAM1,NLRC4,NOD2,TLR4,TLR7 |
| Dendritic Cell Maturation | 8.92 | CCR7,CD40LG,CD83,CD86,FCGR2B,ICAM1,LTBR,NFKBIA,STAT1,STAT2,TLR4,TNFRSF1B |
| Hepatic Fibrosis / Hepatic Stellate Cell Activation | 8.84 | BCL2,CCL5,CCR5,CCR7,CD14,CD40LG,ICAM1,IL10RA,STAT1,TGFB2,TLR4,TNFRSF1B |
| MIF Regulation of Innate Immunity | 8.25 | CD14,FOS,NFKBIA,PLA2G2A,PLA2G4A,PTGS2,TLR4 |
| Communication between Innate and Adaptive Immune Cells | 8.18 | CCL4,CCL5,CCR7,CD40LG,CD83,CD86,CXCL10,TLR4,TLR7 |
| MIF-mediated Glucocorticoid Regulation | 7.3 | CD14,NFKBIA,PLA2G2A,PLA2G4A,PTGS2,TLR4 |
| Th1 Pathway | 7.29 | CCR5,CD3G,CD40LG,CD86,ICAM1,IL10RA,IRF1,NFIL3,STAT1 |
| Th1 and Th2 Activation Pathway | 7.05 | CCR5,CD3G,CD40LG,CD86,ICAM1,IL10RA,IRF1,NFIL3,S1PR1,STAT1 |
| Inflammasome pathway | 6.97 | CASP1,NLRC4,NOD2,PYCARD,TLR4 |
| T Cell Exhaustion Signaling Pathway | 6.95 | CD86,FOS,GZMB,IL10RA,IRF9,LAG3,STAT1,STAT2,TCF7,TNFRSF14 |
| Systemic Lupus Erythematosus Signaling | 6.83 | C5,C6,C8B,C9,CD3G,CD40LG,CD86,FCGR2B,FOS,KNG1,TLR7 |
| Pathogenesis of Multiple Sclerosis | 6.81 | CCL4,CCL5,CCR5,CXCL10 |
| iNOS Signaling | 6.54 | CD14,FOS,IRF1,NFKBIA,STAT1,TLR4 |
