# Supplementary material for: Comparison of transcriptional responses between pathogenic and nonpathogenic hantavirus infections in Syrian hamsters using NanoString
Source: PLoS Negl Trop Dis. 2021 Aug 2;15(8):e0009592. doi: 10.1371/journal.pntd.0009592 (PMC8360559; doi:10.1371/journal.pntd.0009592)
Supplement: S2 Table — (DOCX) [file pntd.0009592.s003.docx]

S2 Table 2. 10 dpi HTNV 25 most significantly enriched pathways

| **Pathway** | **-log(p-value)** | **Molecules** |
| --- | --- | --- |
| Interferon Signaling | 21.7 | BAK1,IFI35,IFIT1,IFIT3,IRF1,IRF9,ISG15,MX1,PSMB8,STAT1,STAT2 |
| Role of PKR in Interferon Induction and Antiviral Response | 13.7 | CASP1,DDX58,EIF2AK2,FOS,IRF1,IRF9,JUN,MSR1,STAT1,STAT2 |
| Systemic Lupus Erythematosus In B Cell Signaling Pathway | 12.8 | FOS,IFIT2,IFIT3,IRF7,IRF9,ISG15,JUN,MYC,STAT1,STAT2,TLR7,TNFSF10 |
| Role of Pattern Recognition Receptors in Recognition of Bacteria and Viruses | 12.5 | C1QA,C1QB,CASP1,CCL5,DDX58,EIF2AK2,IRF7,OAS2,TLR7,TNFSF10 |
| Activation of IRF by Cytosolic Pattern Recognition Receptors | 12.5 | DDX58,IFIT2,IRF7,IRF9,ISG15,JUN,STAT1,STAT2 |
| Coronavirus Pathogenesis Pathway | 11 | CASP1,CCL5,DDX58,FOS,IRF7,IRF9,JUN,STAT1,STAT2 |
| Neuroinflammation Signaling Pathway | 6.86 | CASP1,CCL5,CXCL10,FOS,IRF7,JUN,STAT1,TLR7 |
| IL-17A Signaling in Gastric Cells | 6.83 | CCL5,CXCL10,FOS,JUN |
| Prolactin Signaling | 6.24 | FOS,IRF1,JUN,MYC,STAT1 |
| Necroptosis Signaling Pathway | 6.2 | CASP1,EIF2AK2,IRF9,STAT1,STAT2,TNFSF10 |
| PDGF Signaling | 6.04 | EIF2AK2,FOS,JUN,MYC,STAT1 |
| iNOS Signaling | 5.66 | FOS,IRF1,JUN,STAT1 |
| Thrombopoietin Signaling | 5.08 | FOS,JUN,MYC,STAT1 |
| Toll-like Receptor Signaling | 4.84 | EIF2AK2,FOS,JUN,TLR7 |
| JAK/Stat Signaling | 4.77 | FOS,JUN,STAT1,STAT2 |
| IL-7 Signaling Pathway | 4.77 | BAK1,JUN,MYC,STAT1 |
| Chemokine Signaling | 4.69 | CCL24,CCL5,FOS,JUN |
| T Cell Exhaustion Signaling Pathway | 4.63 | FOS,IRF9,JUN,STAT1,STAT2 |
| Role of Macrophages, Fibroblasts and Endothelial Cells in Rheumatoid Arthritis | 4.5 | CCL5,CSF1,FOS,JUN,MYC,TLR7 |
| IL-17A Signaling in Fibroblasts | 4.38 | FOS,JUN,LCN2 |
| Complement System | 4.27 | C1QA,C1QB,C4A/C4B |
| Antigen Presentation Pathway | 4.24 | PSMB8,PSMB9,TAPBP |
| Renin-Angiotensin Signaling | 3.98 | CCL5,FOS,JUN,STAT1 |
| Pathogenesis of Multiple Sclerosis | 3.89 | CCL5,CXCL10 |
| Colorectal Cancer Metastasis Signaling | 3.87 | FOS,JUN,MYC,STAT1,TLR7 |
|  |  |  |
|  |  |  |
|  |  |  |
|  |  |  |
|  |  |  |
|  |  |  |
|  |  |  |
|  |  |  |
|  |  |  |
|  |  |  |
|  |  |  |
