# Supplementary material for: Comparison of transcriptional responses between pathogenic and nonpathogenic hantavirus infections in Syrian hamsters using NanoString
Source: PLoS Negl Trop Dis. 2021 Aug 2;15(8):e0009592. doi: 10.1371/journal.pntd.0009592 (PMC8360559; doi:10.1371/journal.pntd.0009592)
Supplement: S3 Table — (DOCX) [file pntd.0009592.s004.docx]

**S3 Table 3.** NanoString probeset design

| Gene Identifier | Accession # | Position | Target Sequence |
| --- | --- | --- | --- |
| ABCB1 | XM_013123485.1 | 2268-2367 | TACAGGATCCAGACTTGCTGTCATTACCCAGAATATAGCAAATCTTGGGACAGGAATTATCATATCTCTAATCTATGGCTGGCAGTTGACACTTCTGCTC |
| ABL1 | XM_005085519.2 | 2564-2663 | CTGGGCATTTGGAGTGTTACTCTGGGAGATTGCTACCTATGGCATGTCACCTTACCCAGGAATTGACCTGTCACAGGTTTATGAGCTTCTGGAAAAAGAC |
| ACSL1 | XM_005066672.2 | 714-813 | GCCGAAGCCATCACCTACATAGTGAACAAAGCTGAGCTCGCTGTGGTTTTTGCTGACAAGCCAGAAAAGGCGAAACTCTTGTTAGAAGGTGTAGAAAATA |
| ACTB | NM_001281595.1 | 737-836 | AGGTCATCACCATTGGCAACGAGCGGTTCCGTTGCCCTGAGGCCCTTTTCCAGCCTTCCTTCCTGGGTATGGAATCCTGTGGCATCCACGAAACTACATT |
| ADA | XM_013124411.1 | 305-404 | AGAACAAGCATTCGGGGTCAAGGCCCGGTCCATACTGTGCTGTATGCGTCATCAACCCAACTGGTCCCTTGAGGTGTTGGAGCTGTGTAAGAAGTACCAT |
| AGER | XM_013125842.1 | 320-419 | TTTCCGGTGCCGGGCGGTAAACAGGCACGGAAAGGAGGTCAAGTCCAACTACCGTGTCAGAGTCTACCAGAATCCTGGGAAGCCAGAAATTGTGGACCCT |
| AHR | NM_001281658.1 | 1410-1509 | CCAGCTCGCTTATGAATTGCATGATCCAACAGGATGAGTCCATCTATCTCTGTCCTCCTTCGAGTGCTGCGCCGCTAGACAGCCATTTTCTCACCCACGG |
| AICDA | XM_013110297.1 | 221-320 | TGGACCCTGGACGGTGTTACCGCGTCACCTGGTTCACCTCCTGGAGCCCCTGCTACGACTGCGCCCGGCACGTGGCTGAGTTTCTGAGATGGAACCCCAA |
| AIM2 | XM_005078251.2 | 899-998 | CCAAGTTTGTGTTCCAAATAACATTAGGGAAAAAGCTGGGAGAACTCCCATGATTAAGAAGCTGAAGACTCAGCCTAGTGGAACAATTGTCAATGGGATG |
| AIRE | XM_005070694.2 | 619-718 | GTGGAAGGGATCCTTATCCAGCAGGTGTTTGAGGCAGGAGGCTCCAAGAAGTGCATCCAGGTTGGGGGCGAGTTTTATACTCCTAGCAAGTTCGAAGACC |
| AKT1 | XM_005068305.2 | 1397-1496 | CCACCTTTCAAGCCCCAGGTCACCTCTGAGACGGATACCAGGTATTTCGATGAGGAGTTCACGGCCCAGATGATCACTATCACACCGCCTGATCAAGATG |
| AKT3 | XM_005078162.2 | 362-461 | CATAGGATATAAAGAAAAACCTCAAGATGTGGACTTACCTTATCCCCTCAACAACTTTTCAGTGGCAAAATGCCAGTTAATGAAAACAGAACGACCAAAG |
| ALOX12 | XM_005067498.2 | 1838-1937 | AGTATTTCTCAGACCCCAGGACCAAAACTGTGCTGAGCCGATTTCAATCAGATTTGGAAAATCTGGAAAGAGAGATTACAGCCCGGAATGAGCAACTTGA |
| ALOX5 | NM_001281587.1 | 1479-1578 | GTCGTTCACAAGTGAGGTGGTAAGCATCTACTACGAAGATGACCAGGTAGTGATGGAGGACCAGGAACTACAGGACTTCGTGAAGGATGTTTACGTGTAC |
| ANP32B | XM_005078732.2 | 671-770 | TTGGAGCCTTTGAAAAAGTTGGATTGTCTGAAAAGCCTGGATCTGTTTGGCTGTGAGGTCACTAATCTGAATGATTACCGAGAGAGTGTCTTCAAGCTCC |
| APOE | XM_005086320.2 | 300-399 | GTCCAGGAAGAACTGCAGACGTCACAAGTCACCCAGGAACTGACGGTCCTGATGGAGGACACCATGACAGAAGTCAAGGCTTACAAAAAGGAGCTGGAGG |
| APP | XM_005073916.2 | 1770-1869 | GGCCAACATGATCAGTGAACCCAGAATCAGTTATGGAAACGATGCTCTCATGCCGTCTTTGACTGAAACAAAAACCACCGTGGAGCTTCTTCCTGTGAAT |
| AREG | XM_005068159.1 | 561-660 | TGACAATGACCTATCCAAGATTGCATTAGCAGCCATAACTGTCTTCGTCTCTGCTGTAACCTTGATAGCTGTTGGCATTGTTGTCATAGTCCAGCTTCGC |
| ARFGAP2 | XM_005064922.2 | 1330-1429 | CAGTGAAGCTCGTGAGAAGTTTGCAGGAGCCAAAGCCATCTCATCTGACATGTTCTTTGGTCGCGAGGTGGATTCTGAGTATGAAGCCAGGTCCCGGTTA |
| ARG1 | NM_001281645.1 | 594-693 | CTCAATGACTGAAGTGGATAAACTAGGAATTGGCAAGGTGATGGAAGAGACGTTCAGCTATCTACTAGGAAGAAAGAAAAGGCCCATCCACCTGAGTTTT |
| ARG2 | XM_005072738.2 | 948-1047 | CTGGATCTCGTTGAAGTCAATCCTCAATTGGCCACTTCAGAAGAAGAGGCCAAGGCTACAGCCAGCCTAGCAGTGGACGTGATTGCTTCAAGTTTTGGTC |
| ARHGDIB | XM_013115456.1 | 230-329 | GATGGATAAAGATGACGAGAGTCTAACCAAGTACAAGAAAACACTTCTGGGAGATGCTCCGGTGATAGCAGACCCAACAGTCCCCAATGTAACTGTTACC |
| ARL1 | XM_005067986.2 | 636-735 | CCAAAGGCACTGGTCTTGATGAGGCCATGGAATGGTTAGTTGAAACGTTGAAAAGCAGACAGTAAGTCCATTCACATCAGGTCCTGTGATGCAAATGCCA |
| ATF1 | XM_005067238.2 | 276-375 | GCACCTCAGCCTGGCTCAACAGTTCAGGGGACTCATATTTCTCAGATAGTCCATCAGGTGTCATCTTTGTCAGAAAGTGAGGAGTCTCAGGACTCATCTG |
| ATF2 | XM_013121780.1 | 897-996 | TGATAAAGATGACAGTTCAGAGGACCTTTCCGTGCCAAGCAGCCCACATACAGAAGCAATCCAGCACAGTTCTGTCAGCACCTCCAATGGAGTCAGCTCG |
| ATF3 | XM_005082874.2 | 212-311 | CCTCTCTCCTCCCGGGTCCCTGGTGTTTGAGGATTTTGCTAACCTGACACCCTTTGTCAAGGAAGAGCTGAGGTTTGCCATCCAGAACAAGCACCTGTGT |
| ATG10 | XM_005065674.2 | 329-428 | CGAGTATCATGTCTTATACTCCTGCAGCTACCAAGTGCCCGTTCTTTACTTTAGGGCAAGCTTTTTAGATGGGAAACCTTTATCCCTGACGGATATCTGG |
| ATG12 | XM_005069139.2 | 167-266 | CCCGGAGCCCCCGTCTTCGGCCGCAGTCTCTCCGGGGACGGAGGAACCTCCCGGGGACACCAAGAAAAAAATTTATTTATGTGAATCAGTCTTTTGCACC |
| ATG16L1 | XM_013122101.1 | 1592-1691 | TTTTAAGGCAGCAGGATCAGCCGAGGGTTCTCTTTATGTCTGGAGTGTGCTCACAGGGAAAGTGGAGAAGGTTCTTTCAAAACAGCACAGCTCCTCCATA |
| ATG5 | XM_005070417.2 | 591-690 | ACATTTCAAGAGTTTTCCAGAAAAGGACCTTCTACACTGTCCATCCAAGGATGCAGTCGAAGCCCATTTTATGTCATGTATGAAAGAAGCTGACGCTCTA |
| ATG7 | XM_013110402.1 | 1029-1128 | ATCTGTGTCAAGCTGAAAGAGTCACGGCCCTACCATACTTCTTAATCAAGTACGATGACAACACCGTGCTGGTCTCCTTGCTCAAACACTACAGTGATTT |
| ATM | XM_013112839.1 | 3324-3423 | TCACCAAGTTCGGATGTTGGCTGCAGGGTCAATTAATAGATTATTCCAGGATGTGAAACATGGAGATTCCTCCAGATGCTTGAAAGCACTACCTTTGAAG |
| B2M | XM_005068531.2 | 254-353 | AGATCTGTCCTTCAACAAGGACTGGTCTTTCTATCTCTTGGCTCACAGGGAGTTTGTACCCACTGCGACTGATAAATACGCCTGCAGAGTTTCACACATA |
| B3GAT1 | XM_005078499.2 | 204-303 | CCTTGGAGATGCCGAAGAGACGGGACATCCTTGCGATTGTCCTCATCGTGCTTCCCTGGACACTGCTCATCACCGTCTGGCACCAGAGCAGCCTCGCACC |
| BAK1 | XM_005084866.2 | 382-481 | GCCTCTGCCAACCCCGAGATGGACAACTTGCTCCTAGAACCCAACAGCGTCTTGGGTCAAGTGGGTCGGCAGCTTGCTATCATTGGAGACGACATTAACC |
| BATF | XM_005086367.2 | 370-469 | CGACAGCGGCAGACACAGAAAGCCGACACCCTGCACTTGGAGAGTGAAGACCTGGAGAAACAGAATGCTGCTCTCCGCAAGGAGATCAAACAACTCACAG |
| BATF3 | XM_005082877.1 | 125-224 | TTCGCAGGAGAGAGAAAAACCGGGTTGCTGCTCAGAGAAGCCGGAAGAAGCAGACCCAGAAAGCTGACAAGCTCCATGAGGAGTATGAGTGCCTGGAGCA |
| BAX | XM_005084711.2 | 400-499 | GCTAGCAAACTGGTGCTCAAGGCCCTGTGCACCAAAGTTCCCGAGCTAATCAGAACCATCATGGGCTGGACACTGGACTTCCTCCGAGAGCGGCTGCTTG |
| BCAP31 | NM_001281944.1 | 244-343 | CTCCAGAACAATCCAGGTGCCATGGAGCACTTCCACATGAAGCTTTTCCGTGCTCAGAGGAATCTCTACATTGCTGGCTTTTCCTTGCTGCTGTCCTTCC |
| BCL10 | XM_005081907.2 | 114-213 | AGGAGGACCTCACTGAAGTGAAGAAGGACGCTTTAGAAAATTTACGTGTTTACCTGTGTGAGAAAATCATAGCAGAGAGACATTTTGATCATCTACGTGC |
| BCL2 | XM_005081454.2 | 1218-1317 | ATGGCGCAAGCCGGGAGAACAGGGTATGATAACCGGGAGATCGTGATGAAGTACATCCATTATAAGCTGTCGCAGAGGGGCTACGAGTGGGATGCCGGAG |
| BCL2L1 | XM_005086058.2 | 818-917 | AGCAGGTAGTGAATGAACTCTTCCGGGATGGGGTAAACTGGGGTCGCATTGTGGCCTTTTTCTCCTTCGGTGGAGCCCTCTGTGTGGAAAGCGTAGACAA |
| BCL2L11 | XM_013112274.1 | 778-877 | TGTTACGCTTCATCGTCCGACTAGTATGGAGAAGGCATTGACAGGATCTTCACGCCACCAGGATAAATGTGGACATCGGTCTTGTTCAGACCACCAGAAT |
| BCL3 | XM_005086350.2 | 491-590 | CATTGCTGTGGTCCAGAATAACAAGGCAGTGGCCCTCCGGTTAGTCCTCCTTTTCCAGCAGGGAGGCCGGGAACTAGACGTCCACAACAACCTACGGCAG |
| BCL6 | XM_005071603.2 | 683-782 | TCAACACCACCGGCCTCATATCCTATGTACAGCCATCTCCCACTCAGTAGCTTCCTTTTCGCTGATGAGGAGCCGCGAGATGCCCATGCCCGCATGCCTG |
| BID | XM_013127166.1 | 127-226 | CCGTCCCTGCTCCGTGATGTCTTCCAAACGACAGTGAACTTTATTAACCAGAATTTACTCACCTATGTGAGGAACTTACTTAGAAATTGCCAGGCTGGAC |
| BIRC2 | XM_013118536.1 | 866-965 | GCCGTTTCGTCATGAACATGGCTCCAGACAGTGCTTTCCTGTCCAGACTGATGAAGGGTGCGGACACCTTTGAGCTGAAGTACGACTTTTCATGTGAGCT |
| BLK | XM_013117245.1 | 1195-1294 | CCAGAGGCCATCCACTTCGGCGTGTTCACCATCAAGGCTGATGTATGGTCCTTCGGAGTCCTGCTGATGGAGATCATCACATATGGGCGCGTTCCCTACC |
| BLNK | XM_005063609.2 | 1214-1313 | GCTGAAGAGGCCCTGCACAGATCCAACAAGGACGGATCATTTCTTATTCGGAAAAGCTCTGGCCATGATTCTAAGCAGCCATATACACTAGTTGCATTCT |
| BST2 | XM_013126528.1 | 560-659 | CAGAGGCGCAGGCGGGCACCTGCAACCGGACCGTGGTGACCATTCAAGATTCTCTGGAGAAGAAGGTGTCTCAGATCCAGGAGCAGCAAACTCGGATCCA |
| BTK | XM_005072490.2 | 1100-1199 | TCAAGGGGTGATACGCCATTACGTTGTGTGTTCCACACCGCAGAGCCAGTATTACCTGGCTGAGAAACACCTTTTCAGTACCATCCCAGAGCTCATTAAC |
| BTLA | XM_013116855.1 | 1216-1315 | AGCCTTCTGACTTAGCAGGAAGGGAAATTAACCTGGTTGATATTCCAGTGAGTCCCAGGATAAATTCCCAGACACTTCTATCAGAAACTGGCATTTACGA |
| C14orf166 | XM_005083571.2 | 274-373 | GAGAAGTATCTCAAAGATGTAAACTGTCCTTTCAAGATTCAGGAGCGGCAAGAAGCAATTGACTGGCTTCTCGGTTTAGCTGTTAGACTTGAATATGGAG |
| C1QA | XM_005081017.2 | 523-622 | AACAAGACAGGCCGCTTCATCTGTGCGGTGCCCGGCTTCTATTACTTCACCTTCCAAGTAACCTCCAAGAGGGACCTTTGTCTGTCTCTCATGTCCTCCT |
| C1QB | XM_005081015.2 | 583-682 | AAAACTATGAGCCACGAAGTGGCAAGTTCACCTGCAAAGTGCCTGGCCTCTACTATTTCACCTACCACGCCAGTTCCCGAGGGAACCTGTGCGTGAACCT |
| C1QBP | XM_005067454.2 | 547-646 | GAAGGCTGAAGAACAGGAGCCGGAACTGACATCAACTCCCAATTTTGTGGTTGAAGTTACAAAGAGTGATGGCAAGAAGACCCTTGTACTGGACTGTCAC |
| C1R | XM_005065935.2 | 760-859 | CATGGTTCCCAGGGGGTACAGGGTGAAGCTCGTCTTCTGGCATTTTGACGTGGAGCCCTCTGAAGGCTGTTTCTATGACTATGTCAAGATTTCTGCTGAT |
| C1S | NM_001281660.1 | 1170-1269 | CAATCCCTTGTCCCAAGGAAATCACCGCCAACTCTGTTTGGGCGCCTGAAAAGGCAAAATACGTGTTTAAAGATGTGGTGAAGATATCCTGTGTGGACGG |
| C2 | XM_005086834.2 | 527-626 | CGGTTGACAACATCAGAAATATCCTGGATATCAATCAGAACAGGAACGACTATCTGGACATCTATGCTATTGGAGTGGGCAACCTGGATGTGGACTGGAG |
| C3 | XM_013121434.1 | 679-778 | GATCCGAGCCTTCTACGAACACTCGCCGCAGCAGATCTTCACGGCAGAGTTTGAGGTGAAGGAGTACGTGCTGCCCAGTTTCGAGGTCCTGGTGGAGCCC |
| C3AR1 | XM_005066031.2 | 743-842 | ACGATACAGATCTACCAGAAAGCAATTCTACTGACAACTCCACTGCTCAGCTAACAGGACAAATGGATGACAGGTCCGATCCTTCCTCTTTCCAAATAAG |
| C4A | XM_013125836.1 | 2543-2642 | CGCGTCCGAGTGTTCCGAGAATTCCACATGCATCTTCGCCTGCCCACCTCTGTCCGCCGCTTTGAGCAGCTTGAACTACGGCCTGTTCTCTACAACTATC |
| C4BPA | XM_005079894.2 | 1228-1327 | CTGGCTATGAGGCTGCTACAAAAGAGCCAATGAGTGTGGTTTGTCAGAAAGATTTCACCTGGAGCCCATTTAAAGGATGCAAGGAGATATGTTGTCCAGA |
| C5 | XM_013121988.1 | 1394-1493 | TTCCTTCAGCCCGACTCCTGGTTTATTACATCGTCACAGGAGAGCAGACAGCAGAATTAGTGGCTGACTCGGTTTGGATAAACATTGAAGAGAAATGTGG |
| C5aR | XM_013125510.1 | 516-615 | GTGGAGTGGCCTGGGTCTTAGCGCTACTCCTCACCATCCCATCCTTCATATTCCGTCAGGTGTACCAAGACCCCTTCTCCGATAAGTTGATGTGTGGCAT |
| C6 | XM_005086514.2 | 671-770 | GAGCCCAGAGGGGATGTCCTTGATAACTCTTTCACTGGAGGAATATGTAAAACTGTCAAAAGCAGTCGAGCAAGTAATCCATACCGTGTTCCAGCCAATC |
| C7 | XM_013125557.1 | 1331-1430 | TTGCTGGTTGTTCGGAACAGTGTAGAAGTGGCCCAGTTCATTAACAATAATCCTGAATTTTTACAACTTGCTGAACCATTCTGGAAGGAACTCTCCCATC |
| C8A | XM_005072184.2 | 1157-1256 | GGTGCTCGACAAAAAGAAAATGGAGGTTGATGGTATTACCACAAGAGATGTCCAAAATTGCATCGGAGGTTCCGTGGGCCTGGAGTTTGGGGATACTTTA |
| C8B | XM_005072185.2 | 1073-1172 | AGATCTCCTTCGTGACTTTGGGACTCACTTTATCACCGAGGCCGTGCTTGGGGGCATTTACGAGTACACACTTATCATGAACAAAGACATCATGGAGCAA |
| C8G | XM_013123333.1 | 776-875 | AGACTGACTACCAGAACTTTGCCATCCTGTATCTGGAGAAGGCAAGGAAGCTGTCTGTGAAACTGTACGTCCGCTCATTGCCCGTGAGTGATTCTGCCCT |
| C9 | XM_005078028.2 | 1019-1118 | TATGCATGTGAAGGGAATGGTTCACCTGGGAAGATTTGTGATGAGAAATCGGGATGTTGTGCTGACAACAACTTTCGTGGATGATATAAAAGCTCTCCCA |
| CAMP | XM_005075080.2 | 234-333 | CAGAATGAAGGAGACAGTGTGTGACAAGGCAACACATCAGATACCTGAGCTGTGTAACTTCAGGGAACATGGGGTAGTGAAGCGGTGTGTGGGGACAATC |
| CARD9 | XM_013123323.1 | 139-238 | GTTATCACTTAAGGACAGAATCCTACAGTTTCCAGAGGGGACAGAGGTGTCCTCTCCTGATGGAGTAGCCACAACCCTATCAGTCCTCATTCATCTCATG |
| CASP1 | XM_005077340.2 | 598-697 | CGAGGGGTGGTGTTGGTGAAAGATTCAGTAGAAGACACTGGAAAGAAATTCTTAGTGGATGCAGATTTGGAAGATGATGGCATTAAGAAAGCCCACATAG |
| CASP2 | XM_005083076.2 | 720-819 | TCTTCAAGCTTTTGGGCTATAACGTCCACGTTCTACGGGACCAGACTGCACAGCAAATGCAAGAGAAACTTGAGAATTTCGCACAGTTGCCTGCGCACCG |
| CASP3 | NM_001281582.1 | 229-328 | CCTCGCTCTGGTACTGATGTTGATGCAGCCAAACTCAGGGAGACATTCATGGCCCTGAAATACGAAGTTCGGAATAAGAACGATCTTACTCGTGAAGAAA |
| CASP8 | XM_005070606.2 | 1272-1371 | GAACAAGACAGCGGCTCGCAGATGTCAAACAAAGTTTACCAAATGAAGAACAAACCTCGGGGATACTGTCTGATCTTCAACAACAATGATTTTAGCAAGG |
| CASP9 | XM_005139261.2 | 960-1059 | CAGGCCAGCCACCTGCAGTTCCCAGGTGCCGTCTATGGCACAGATGGATGCTCCGTGTCCATCGAGAAAATTGTGAACATCTTCAACGGGGCTGGCTGTC |
| CCBP2 | XM_005082371.2 | 316-415 | CCCTTCTGGGCCATCTCCGTGGCCTGGCAGTGGGTTTTTGGCAGTTTCTTGTGCAAGGTGGTGAGCATTCTCTACACGATCAACTTCTACTGTGGCATCT |
| CCDC117 | XM_005087802.2 | 1037-1136 | CCTGTGTTCAGTAATGAGCCTGCCTGTATAGTATAGGGACCTGAAAGTTTTATGAAACGGGTGTAATATCTCCACCTGTGATTTAGGGTGGGACTGTCAT |
| CCL1 | XM_005076964.1 | 155-254 | AGATCAGCTCCTCCTGTTCCCACCCAGCTGTTGTATTCAGGCTGAAGAAAGGCCAAGAGAGCTGTGCCTTGAGTACAAGTGCTTGGGTTCAAGATTACCT |
| CCL11 | XM_005076966.2 | 102-201 | TCTATCCCAGTTTCCTGCTGCTTTGCTATGACCAGTAAGAAGATCCCCAAGCCACTGCTGAAAAGCTACAAAAAAATCACCAACAGCAGATGCACCCTGA |
| CCL16 | XM_013118284.1 | 536-635 | AGCAGACCAGTGGCCAAAATAAAGAGCAGTGCTTTGAATTGTCTCAGTTTTGAAAGAAAATGTCAGGGTTAGGGGAAGTAGAAAGTAACTCCTCTCTCTC |
| CCL17 | XM_005078618.2 | 200-299 | CAGCTGAGTGTCCCAGGGATGCCATCGTGTTTGTGACTGTCCAGGGCAGGTCCATCTGTTCAGACCCCAACGACAAGCATGTGAAGAAGGCCATCAGACA |
| CCL19 | XM_005139250.2 | 319-418 | TCCTGGGAACATCGTGAAAGCCTTTCGCTACCTTCTTATCAAGGACGGATGCAGGGTGCCTGCCGTTGTGTTCACCACACTAAGAGGCTACCAGCTCTGC |
| CCL2 | XM_005076967.2 | 355-454 | AAAACAGCACCTTTGAATGCTAACTTGACGCAAGCTCCTGCAGTTAATGTGTCTACTACTGCCTTTCCCACCGCAGACTCAAGAACTTCTGTAAGACTGA |
| CCL20 | NM_001281699.1 | 100-199 | TGAGAATGTTTTGCAGTGGCAAGAGCGTGCTCTTCATTGCTCTGGCATGGGTGCTGCTAGCTCACTTCTATAGTCAGTCAGAAGCAAGCAACTTTGACTG |
| CCL21 | XM_005078820.2 | 135-234 | GAGGTCAGGATTGCTGCCTCAAATACAGCCAGAGGAAGATTCCCTACAGTATTGTCCGAGGCTACAGGAAGCAAGAGCCAAGCTTAGGCTGTGCCCTCTC |
| CCL22 | NM_001281664.1 | 142-241 | CCCTGCCGCCGCGCGTAGTGAAGGAGTTCTTCTGGACCTCAAAGTCCTGCCGCAAGCCTGGCGTTGTTTTGATAACCATCAAGAACCGGGATATCTGTGC |
| CCL24 | XM_005080365.2 | 101-200 | CACGTGCCGGGTCTACCCTACAGACTCTGTGACCATCCCGTTTTCTTGCTGCATGTCCTTTATTACCAAGGAAATTCCAGGAAACCGAGTGGCTAGCTAC |
| CCL3 | NM_001281338.1 | 118-217 | TCTATGGCCGGCAGATTTCCCGCAAATTCATCGCCGACTATTTTGAGACCAGCAGCCTTTGCTCTGAGCCAGGTGTCATTTTCCTAACCAAGAGAAACCG |
| CCL4 | XM_005076934.2 | 120-219 | TTGGAAGATTCCTCGAATGTTTGTGACAGATTACTATGAGACCAGCAGCCTTTGCTCCAAGCCAGCTGTGGTATTCCTGACCAGAAAAGGCAAGCAAATC |
| CCL5 | XM_005076936.2 | 153-252 | CTGCCTCGTGTTCACATCAAGGAGTATTTCTACACTAGCAGCAAATGCTCCAACTTTGCAGTCGTTTTTGTCACCCGAAGGAACCGTCAAGTGTGTGCCA |
| CCL7 | XM_005076968.2 | 129-228 | GAAGTATCTCGAGAGTTACAAAAGGATCACCAGTAGTCACTGCCCCTGGGAAGCTGTGATCTTTAAGACCAAGAGGGGTGTGGAAATCTGTACTGAAGCC |
| CCL8 | XM_005077080.2 | 406-505 | GCATAAGCCTTCACACCTGGGCTGAGAGACAGTCAGAGCTTGAAGTTCTTCCCTAACTTCTCCAGGCATGGATTTGTTACAAGAAGATGGTGTCTGGTTT |
| CCND3 | XM_005072382.2 | 532-631 | GCCCCTGACTATTGAGAAGCTTTGCATCTATACGGACCAAGCTGTGGCTCCTTGGCAGTTGCGGGAATGGGAGGTGCTGGTCCTGGGGAAGCTCAAGTGG |
| CCR1 | XM_005082282.2 | 492-591 | TATCATTACCTGGGTCCTAGCCATCTTGGCCTCCATTCCCGGCTTATACTTTTTCAAAGCCCAGTGGGAGTTCACGCACAATACCTGTAGTCCTCATTAC |
| CCR10 | XM_005070150.2 | 39-138 | CTACTCCGGATACGATGAGGCTTACTCGGTTGGGCCGTTGCCGGAACTCTGTTACAAGGCGGATGTCCAGGCTTTTAGTCGGGCCTTCCAACCCAGTGTC |
| CCR2 | XM_005082361.2 | 63-162 | GGAAGACAGCGATATGTTTCCTCAGCTCATCCATGGCATATTATCAACAAAAAATATCCAAGAGTTAGATGAAGACTTCACCACACCTTATGACTATGAT |
| CCR3 | XM_013122202.1 | 540-639 | CATCTTCGGAGAACATTTCTGTGGTCCTCATTATCCAGAGGATGAAGAAGACAGCTGGAAGCGTTTCCATGCTCTGAGAATGAATATCTTTGGTCTAGCT |
| CCR4 | NM_001281562.1 | 428-527 | TGTTTTCCTTGAAAGCGAGGACTCTGACCTATGGGGTTATCACCAGCTTGGTCACGTGGTCAGTGGCTGTATTTGCCTCCCTCCCAGGCCTCTTGTTCAG |
| CCR5 | XM_005082278.2 | 320-419 | CATGCTCTTCTTGCTCACTCTCCCAGTCTGGGCTCACTATGCTGCAGATGAGTGGGTCTTTGGGGGTATAATGTGCAAATTATTCACAGGGCTCTATCAC |
| CCR6 | XM_013122747.1 | 1022-1121 | CCGTGCTGTACGCATTCGTTGGGCAGAAGTTCAGAAACTACTTCGTGAAGATCATGAAGGATGTGTGGTGTGTGAGAAGAAAGAACAAGGTGCCGGGCTT |
| CCR7 | XM_005075968.2 | 339-438 | CTGGGCTTATAGTGCCGCCAAGTCCTGGATCTTTGGTGCCTACCTGTGTAAGTGCATCTTCGGCATCTATAAAGTGAGCTTCTTCAGCGGCATGCTGCTT |
| CCR8 | XM_005082334.1 | 233-332 | CCGACCTGCTTTTCGTCTTCTCCGTCCCCTTCCAGACTCACTACCTGCTGGACCAGTGGGTGTTCGGGACTGCGGTGTGTAAAGCGGTGTCTGGCTTTTA |
| CCRL1 | XM_005074829.2 | 1327-1426 | CCCTCAAGGTTCTGCTCACAGTGGTTGTAGTTTTCATTGTCACCCAGCTGCCGTATAACATTGTCAAGTTCTGCCAAGCCATAGATGCCATCTACTTGCT |
| CCRL2 | XM_013116979.1 | 737-836 | GTGGTTTTTATACTCTGCTGTGGGCAAATGAGGAAAACGCAGGCCTTCAGGGAGAGGCAGAATGATCTTCGAAAGCTTGGTTTTGTCATAATGGCTGTGT |
| CD14 | XM_013112657.1 | 1207-1306 | TGCGTTTAACCCTGGCAGTGAGTGTGTCAGGAACTTTGGCTGTGCTCCAAGGAGCTGGCCTTTTTGCCTAATACCAAAGGAAGTAAAAGGAACATTTACC |
| CD160 | XM_013123798.1 | 792-891 | GCTAAGGGTTAAAAGGGATCCTGGGACGGATAGCATCACTGAACAGTCATCACAGTTGGTGTTCACCATAGAACAAGCCACGCCATCAGACAGCGGGACC |
| CD163 | XM_013110291.1 | 1439-1538 | AAAGACAAGTGGCTTCAGTTATATGCTCAGGAAACCAATCCCAGACACTACAGCCATGTAGTTCATCGTCCTCGGTCCAAACAACGAGCTCTACCATTCC |
| CD164 | XM_013115813.1 | 285-384 | GATGTCACCATTAATAATACTACCTGCTTTTGGGTAGAATGCAAAGAAGCTAATAAAACGTATTGTTCAAGTGTGCCCATGAGTAACTGTAGCATGGCGA |
| CD1D | XM_005080275.2 | 350-449 | ATCGAACCAGCTTTACCAGGGACATACAGGAATTAGTCAAAATGTTGCCTAGCATGCACTATCCCATTGAGATACAGTTGTCTGCTGGGTGTGTACTGCA |
| CD2 | XM_005076665.2 | 315-414 | TGTGATAGTGTATGGTGCCAATGGCAGTAGTAAGCTACAAAAAGCATTCCACTTGAGGATTCTAGAGAAAGAGCTGCATTTCTATGTCATCGTGGGGGCC |
| CD205 | NM_001281884.1 | 2466-2565 | CCCCGGTTATAATTGATGGCAGTGAATACTGGTTTGTTGAGGAACCCCGCTTAAACTACGAAGAAGCCGTCCTGTACTGTGCTAGCAACCACAGCTTTCT |
| CD209 | XM_005085279.2 | 833-932 | ACCAGGTAGCCTGTCTTCTGCATGGCTGGACCACTTTTGTATAATCCTCTGGTGTCTGCGGGTTCTCTTTGGGTTAGTCGTTATGGCTCTGCTTCACCTT |
| CD22 | XM_005081870.1 | 366-465 | TGATACCAAGAAATTCAAGGGGAGTGTCCTCTATAATAACACCAGGATCGAGTCTTCTCCTTCTGAATATGGAAGGGTGACGTTCATGGGAAACAGAAGA |
| CD24 | XM_005086648.2 | 123-222 | CTCTACTTTCCCAAATACAACTAATGCCACCACCATAGCAGGTGGCAGTGCCCTGCAGTCAACAGCTAGTCTCCTCATCGTCTCACTTTCTCTCCTACAT |
| CD244 | XM_013119128.1 | 1564-1663 | CCACTGCAAGATCAAGGTTGTTAAAAATCTCCCAAGTTTACAGACCAGACAAGAACAGGTTGAGAGGTTGTTCTGGCCACACTCTGAGATGTTGTAATCC |
| CD247 | XM_013114292.1 | 109-208 | AAACTCTGCTACCTGCTAGATGGAATCCTCTTCATCTAAGGAGTCATTGTCACAGCCCTGTACCTGAGAGCAAAGTTCAGTGGGAGTGTAGATGCTACCG |
| CD27 | XM_005084050.2 | 1091-1190 | GTTCCACCTGCCAGAGAGACACTATCTACAGACACACGTACTTCCTGCTTTGGTTTCATCTTCTCTAGAAGTCGACGCTGCTGTGAGCAAAGTAGAGAGT |
| CD274 | XM_005063709.2 | 370-469 | ATGCGGGTGTTTACGGTTGTATGATCAGCTATGGCGGAGCAGACTACAAGCGGATTACACTGCAAGTCAATGCCCCATACCGAAAAATCAACCACAGAAT |
| CD276 | XM_005069624.2 | 233-332 | ACAGCCCTGGGAGTACTGTACTTCTGCCTCACAGGAGCTGTGGAGGTCCAAGTCTCTGAAGACCCCGTGGTGGCCTTGGTGGACACTGACGCCACCCTAC |
| CD28 | XM_013113684.1 | 364-463 | CTGTTGTGGGCATTGGTCGGGGCTGCTGGAGTCCTGCTTCTTTATGGCTTGGTAGTGACAGTGGCTCTTTGCGTTATCTGGAAAAATAGCAAAAGGAACA |
| CD34 | XM_005082843.2 | 728-827 | ATCAAGGGAGAAATCAAATGTTCAGGAATTCGGGAAGTGAGATTGGCCCAGGGCATCTGCCTGGAACTAAGTGAAGCATCTAGCTGTGATGAATTTAAGA |
| CD36 | XM_005080878.2 | 1344-1443 | CTTCGCTTCACCGCTCCAGAATCCAGACAATCACTGTTTCTGTACTGAACAGGTCATCTCGAATAACTGTACATCATACGGTGTGCTGGACATCAGCAAA |
| CD38 | XM_005068715.2 | 654-753 | TGTCTCAGTGTTCTGGAAAGTGATTTCCCAAAAGTTTGCAGAAGCTGCCTGTGGTGTGGTCCAAGTGATGCTCAATGGGTCCCTCAGTGAACCATTTTAC |
| CD3D | NM_001281420.1 | 72-171 | GAGCTCCTACAAGATACCGGTGATTGAATATGAGGACCAAGTATTTGTGAGTTGCAATACCAGCATTGTGTATCTAGAGGGAACGGCGAAAGTATGGTTT |
| CD3E | XM_013112878.1 | 376-475 | GAGAATTGTGTGGAGGTGGACCTGACAGCAGTAGCCATAATCATCATCGTTGACATCTGCATCACTCTTGGCTTGCTGATGGTCGTTTATTACTGGAGCA |
| CD3EAP | XM_013125449.1 | 960-1059 | CCCCTCGTTTCTCCTTGGAGGCGCTGACATTCCCGGATACAGAAGTGTGGCTCATCCGAGCCCCTGCGGACTTCGCCCCACAGTGCCTCAATGGGCGGCG |
| CD3G | XM_005069273.2 | 246-345 | CACTTATTGGTGTCAAGGAGCAAAGGACAAATCAAAGACACTCCAACTGTATTACAGAATGTGTGAGAACTGCATTGAGATAAATATAGGCACCATATGC |
| CD4 | XM_005065933.2 | 53-152 | AGAGTGAGCGGTTTAGCCGTTTCGACTCAAGAAAAGCTGCGTGGGACCGAGGATCATTCCCTCTCGTCATCAATAAACTTAAGGTGGAAGACTCCAACAC |
| CD40 | XM_005084980.2 | 601-700 | CCGGATGCGAGCCCTACTGGTCATTCCCATCGTGATGGTCATCCTCACCACCATCTTCATAGTGGTGTCTTTCTGTATCAAAAAGGTGGTCAAGGAACCA |
| CD40LG | XM_005084522.2 | 595-694 | GGATCTGAGAGAATCTTACTCAGGGCGGCAAACACCCATAGTTCCTCCAAGCCCTGCGGCCAACAGTCTGTTCACTTGGGAGGAGTATTTGAATTACAAG |
| CD44 | NM_001281873.1 | 529-628 | GTTACCATAACTATTGTCAACCGTGATGGTACCCGCTACAGCAAGAAGGGCGAGTATAGAACACACCAAGAAGACATTGATGCCTCAAATACCACAGATG |
| CD46 | XM_013122615.1 | 1494-1593 | TCACACTTTCATCGCACGTAATGGCTGTGCATTAAGCTCATGTCTGTGTCTCCCACACTCTTACCCTAGTGTTACCCAAAATGCTCAATTCTCTCCAGAT |
| CD48 | XM_013119164.1 | 47-146 | TAGCATGTGCTGCTTCAGAAAATGGGAATGGTGTCTGGTCCTGGAACTGCTACTCCTCCTGCCTCTGATAACAGGACTTCCAGATCATTCAGGATCAAAA |
| CD5 | XM_013110898.1 | 973-1072 | CTCATCTGTACCCAGGAGAAACTGTCTCTGTGTTACCAGCTTCAGAAAAAAACCAACTGCAAGAGGGTGTTCGTCACATGCCAAAACCCAAACCCAGCAG |
| CD53 | XM_005076593.2 | 361-460 | TTCTGCTGATGATTATTCTTCTTGCTGAGGTGACCTTAGCCATCCTGCTCTTTGTGTATGAACAAAAGCTGAACACTTATGTGGCAGAGGGCCTGAATGA |
| CD55 | XM_013120364.1 | 1649-1748 | AAAGGCCCACATTTCATACCAAGGCTCTCCAGATTGTTACCCAAAGATTCACTTCTGCTCATATTACAGCAACTCAGCATGTACCTGCTACCAAGACAAC |
| CD59 | XM_005064546.2 | 314-413 | TGCCACTTGTTCAGTTAACCTGAATTCCTGTCTCATCGCTGTATCCGGAAAGCAATTCTATCATCAGTGTTGGGAATTTTCAGAATGTGATGCCAGCACC |
| CD6 | XM_013112052.1 | 1077-1176 | CTGCTCAGGCTCCCGAAGACATCACAACCTGTCCACTTCTGCAGTGCCTTCTCAAGTGCCGGGCGCCATAGAATCTTCTGTGCCATCGAGTGTGAAGGAC |
| CD68 | XM_005067542.2 | 466-565 | CACAGTTCATCCGACAAGTAATAGCACTGCTACCAGCTACGGATCCACAACTTCCACTCATAATCCTGCTGTGACCACCAGTCCTGGGAATGCCACAGTT |
| CD69 | XM_005087138.1 | 106-205 | TCCATTCAAGTTCCTATCCCTTGGGCTGTGCTGATCGTGGTCGTCATTACTTCCTTAATTATAGCTCTAGTTGCCTTGAGTGTGGGCCAGTACAATTGCC |
| CD7 | XM_005070173.2 | 195-294 | CAGGTTTCCGAAGTGACTTACTTTGAAGACGGGAAGGAGTCCACGGTAGACAAGCGTTTCTCGGGCCGAATTGACTTCTCGGGTTCCCAGAGCAACCTGA |
| CD70 | XM_005081413.1 | 135-234 | CAGTATTGGGATTTGCTGCTTGATCATCTGTGGCCAGTTATGGAGGCCGAAGCAGACGCAGCTGAATCCACTTGAGGTGCATATAGCTGAATTACAGCTG |
| CD74 | XM_005074693.2 | 360-459 | GACCGTCACCTCTCAGAACCTGCAACTGGAGAGCCTTCGCATGAAGCTTCCTAAATCTGCCAAACCTGTCAGCCAGATGCGGATGGCTACTCCCTTGCTG |
| CD79B | XM_005070012.2 | 417-516 | CAACAGCGCCAACTTAGATACTGCCTGGAGCTGTGGCTCGGAACTTAAAGTCTTAGGATTCAGCACCTTGGAGCAACTGAAGCGGCGAAACACACTGAAG |
| CD80 | NM_001281419.1 | 644-743 | GTTCAGAAGAAAGAAAGAGGGTCTTATGTACGGAAGCACTTGACTTCTGTGGAGCTGTCCGTCAGAGCTGACTTCCCAGTTCCTAACATTACTCAGTTTG |
| CD81 | XM_005064144.2 | 531-630 | TGCCTGTGAGGTGGCTGCTGGCATCTGGGGCTTCGTAAACAAAGACCAGATTGCCAAGGATGTGAAGCAGTTCTATGACCAGGCTCTTCAACAGGCTGTG |
| CD82 | XM_005064889.2 | 383-482 | TCATGGGGCTCATGGGTTGTATCGGTGCTGTCAACGAGGTCCGCTGCCTGCTGGGGCTGTACTTCGTCTTCCTTCTGCTCATCCTGATCGCACAGGTGAC |
| CD83 | XM_005066343.2 | 423-522 | CAGAGCAGGCAAAACAACTCCTCTGAGGCACCCGGGACAAAGTCTTATTCCCTGACGATCCAAAACACCACCATTTGCAGCTCTGGTACCTACAGGTGTG |
| CD86 | NM_001310555.1 | 655-754 | TGCAGATATCACAAGACAATGTCACAGAACTGTTCAGCGTTTCCATTAGCCTGTCTATTCCATTCCCGGACGGTGTGTACAATGTGACAGCCTTGTGTGT |
| CD8A | XM_005076083.1 | 303-402 | CCTGACCCTGGAGAATTTCAGCAAGGAATTTGAAGGCTACTATTTCTGCTCTGTCACAGGAAACTCGGTGGTGTACTTCAGTCCTCTTGTACCAGTCTTT |
| CD9 | XM_013123609.1 | 695-794 | CCTCCTGGTGATATTCGCCATTGAGATAGCTGCGGCCGTCTGGGGCTACACCCACAAGGATGAGGTGATTAAGGAAGTCCAGGAATTTTACAAGGACACC |
| CD96 | XM_005074811.2 | 683-782 | AAACATTCTCTTGCCGCCTGACAGTCAATCCTCTTAAAATCTGGAAGACCTCCACTACAGTCAAGGTATTCGCTAAGCCAGAAATCCTCCTGATTGTGGA |
| CD97 | XM_013126321.1 | 1432-1531 | CACGGACACTAGGAAGCTCGCCTCCAACGTCACATTCAACTTCTCCTATTCTGTGGGGTCAAAAAAGCGGCCACGTGAAGAGCTGATGTGTGCCTTTTTG |
| CDC42 | XM_005081021.2 | 282-381 | TGGAGAGCCATATACTCTTGGACTTTTTGATACTGCAGGGCAAGAGGATTATGACAGACTACGACCGCTGAGTTACCCACAAACAGACGTTTTCCTAGTA |
| CDH1 | DQ237892.1 | 250-349 | GGATGTAACTCAGTTAAGGTTCTGGAGATGAGATTGGATTATGTAGATGACCATGACTTTAATGACAAATGTCTCGGAGGGGAAAGATGGACAGGGGAGG |
| CDH5 | XM_005078647.2 | 1448-1547 | GACCCAGACAAGGCTCAGCGCACCATTGGATACTCCATCCGTAAGACCAGTGACAGAGGGCTATTCTTTGGAATAACCAAACAGGGGGACATCTATAATG |
| CDKN1A | XM_005084910.2 | 1558-1657 | CTGGAGGGCTATTCAAACTAACCGGAGATTGAGAGCGGCGGCTGACCCTTACCTAGGACCGAATCTCTGTGGGTGTAGAAGCACTTACTAAGACTGACCC |
| CEACAM1 | XM_013125976.1 | 2045-2144 | CTCCCTTGGCCAGGATTGAAAGGCCTCCCAAGCCCTTTTGATCAGGAGTTCCAACCCAAAATACATAAATGAATAGCAAGGCAGTCACTTGCAGAACCAC |
| CEBPE | XM_005088231.2 | 233-332 | CCCACTACCTGCCGGCTGACCCTCGGCCATTTGCTTATCCCTCGCATACGTTTGGCCCAGATAGGAAGGCTTTGGGGCCTGGCATTTACGGTAACCCGGG |
| CENPF | XM_013122286.1 | 4132-4231 | GTGAGCTCTAAGATGTCAGAGCTGTGTTCCTATGTTGACACATTAAAAGCTGAAAACTCTGTCTTGTCAGTGAATCTGAGAAACTTGCAGGATGACTTGG |
| CFB | XM_005086836.2 | 1628-1727 | ATCAATGCCTTGGCTTCCAAAAAGGACAACGAGCAGCACGTGTTCAAAGTCAAGGACATGGAGAGCCTGGAGGACGTTTTCTTCCAGATGATTGATGAAA |
| CFD | XM_013122939.1 | 550-649 | GTCTGCGGAAACCGCAAGAAGCCGGGCGTCTTTACCCGCGTGTCGACCTACATAGACTGGATCGAAAACGTCAGGCTTGGTAACTGGACGGTCTGAGGTG |
| CFH | XM_013115851.1 | 475-574 | GTGAGATTGATTACCGGGAATGTGATGCTGATGGGTGGACCAATGATGTTCCATTATGTGAAGTTGTTAAGTGTTTGCCAGTGACAGAACCGGAGAATGG |
| CFI | XM_013122607.1 | 648-747 | CAGTTTGGCGGAGTGCACCTTTACCAAGAGAAGAACTAGCGGTTACCAGGGCTTAGCTGGTGTAGCATGTTACACACAGGATACAGATTCTCCATCAAAT |
| CFL1 | XM_005088397.2 | 257-356 | GAGGAGGGCAAGGAGATTCTGGTAGGAGATGTGGGGCAGACTGTGGACGACCCCTACACCACTTTTGTCAAGATGCTGCCAGACAAGGACTGCCGCTATG |
| CFP | XM_005085711.2 | 766-865 | CTTTCTCACTTCCCTTGCACTGACCTTCCACACACATGCTGACCACAAGAACCTCACTTTTAGGAAAGAGGTGGGTTTTCAAGAAGGAAGATAAAGCAGG |
| CHIT1 | XM_005073371.2 | 371-470 | AACAGACGGACCTTTGTCAACTCAGCCATAGTGTTCCTGCGCACTCATGGTTTTGATGGCCTTGACCTTGACTGGGAGTTCCCAGGAAGCCGTGGGAGCC |
| CHUK | XM_005063554.2 | 1738-1837 | CTCTGGAACAACGTGCCATTGATCTCTATAAGCAGTTAAAGCACAGACCTACAGATCACTCTTACAGTGATAGCACGGAGATGGTGAAGATCATTGTGCA |
| CIITA | XM_013124007.1 | 1031-1130 | CCTCCCCATGCCAAGAGGGTCCCGAGTCTCCCATCAAGCTTCCGAAATGGCCGGCGTCTGTGGAGCGGTTCCAGCACTCCCTGAAGGACAAGTACCAGGT |
| CISH | XM_005074871.2 | 660-759 | CTGTTCACGCTGTCCGTCAAAACCACCCGTGGCCCAACCAATGTACGTATCGAGTATGCTGATTCTAGCTTCCGACTGGACTCCAACTGCTTGTCCAGAC |
| CLDN1 | EU856105.1 | 117-216 | CCCCGTCAATGCCAGGTATGAATTTGGTCAGGCCCTCTTCACTGGCTGGGCTGCTGCCTCCCTTTGCCTCCTGGGAGGTGCCCTACTTTCTTGCTCTTGT |
| CLEC4A | XM_013110294.1 | 826-925 | CTGAAGAAAGCAGGTCTCTTCTCAATATGGCCTCACCAAACATTTACACTGACGTGAACTTCAAAACTCAACCTGTTTCTGCAGGCAGTATCTCAGACTC |
| CLEC4E | XM_005065936.2 | 265-364 | GAGCAGAAAAAGTTACTGTCACATGAAAGTGTCAAGGAGCTCTCGTGCTACAGTGTCGCATCAGGTTCAGTCAAGAACTGCTGTCCTTTGAACTGGAAAC |
| CLEC5A | XM_013125017.1 | 561-660 | TCAACAACTCTGTGTTCAGTGGCAATGTTACGAACGAGAACCAGAACTTCCACTGCGTCACGATAGGCCTGACAAAGACATTTGACGCTGCATCTTGTGA |
| CLEC6A | XM_013110293.1 | 758-857 | GTAAAAGTGTCAGGTTCTGGCACCCAAATGAGCCCAATCTTCCCGAAGAGCGATGCGCTTCGATTGTTTACTGGCATCCTATGAAATGGGGCTGGAATGA |
| CLEC7A | XM_005087132.2 | 372-471 | GCATGACAAGAGCTGTTATCTGTTTAAGATGTCACAAAATTCCTGGGCTGGAAGTAAGAGACACTGCTCCCAGCTAGGTTCTCATCTACTGAAGATAGAC |
| CLU | XM_013117285.1 | 260-359 | ATAAGGAAATTCAAAATGCCGTCCAGGGAGTGAAACAGATAAAGACCCTCATAGAAAAAACCAATGCAGAGCGCAAGTCACTGCTCAACAGTTTAGAGGA |
| CMA1 | XM_005085626.2 | 725-824 | AGATCTTGAGGGAGAATTAACTCTGGAGCTTGGAGCCTGTGATTAAATCTGAAGCTGGAATTGAGCAGGTCTTTTGCACCATGTGCTTGGGCCTGTCTTA |
| CMKLR1 | XM_005078925.2 | 539-638 | CGGCTGGCCTATATGACCTGTGTGGTGGTCTGGGTCCTGGCTTTCTTCTTGAGTTCCCCATCTCTTGTCTTCCGGGACACGAACAATGCAAACGGGAAAA |
| CR2 | XM_013122618.1 | 2100-2199 | TCTTATTGGAGAGAAGACTATCTATTGTGCCAGCGACAGTAAGGGCATTGGGATCTGGAGTGGCCCTGCTCCACATTGTGTACTTTCAATTTCTGCGGTT |
| CRADD | XM_013111918.1 | 975-1074 | AAAACAAAAGACCATGCTACCATGTTGCGTGCAGGTGTCCTACATACAAAGCATTTACGTTGCCTGGAAACTGGACTGTGGACTTAACTGTTCATAATGA |
| CREB1 | NM_001281571.1 | 323-422 | CAACTATTGCAGAAAGTGAAGATTCACAGGAGTCTGTGGATAGTGTAACCGATTCTCAGAAACGAAGGGAAATTCTTTCAAGGAGGCCTTCCTACAGGAA |
| CRP | XM_005078194.2 | 318-417 | CCAAGAACAGCCCTAACGAAATCCTCATATTTTGGTCTAAGGATAGAGGGTATGCTTTTGGAGTGGGTGGGCCTGAAGTACTATTCAAGGCTTCTGAAAT |
| CSF1 | XM_005076608.2 | 1135-1234 | GCTTCACTGATGATTACGAAGAACAGGACAAGGCCTGTGTGCGAACTTTCAATGAGACTCCCCTCAAGCTTCTGGAGAAAATCAAGAATGTCTTTAATGA |
| CSF1R | XM_005074685.2 | 2536-2635 | ATTTTCTCACTCGGTCTGAATCCCTACCCAGGCATCCTGGTGAACAGCAAGTTCTACAAACTGGTGAAGGACGGATACCAAATGGCCCAGCCTGTTTTTG |
| CSF2 | XM_005067904.1 | 149-248 | AGGTGAACATAGAAGTAGAAATTGTCTCTAAGGAGTTCTCCATCAAGAGTCCAACCTGTGTGCAGACCCGCCTGAAGGTGTACGAGAACGGCCTACGGGG |
| CSF2RB | XM_005088753.2 | 993-1092 | TCAAGAAGCCTCCTTGCCAGCCCATGCCCCAGGTACCAGTCATTCAGTTTTTTAAGTCCCTGAAGCATCAGGACTACCTGTCACTCCCTGCTTGGGACAG |
| CSF3 | XM_005076035.1 | 142-241 | AGCTTCCTGCTGAAGTCCTTGGAGCAAGTGAGGAAGATCCAGACCAGGAACTCGGAGCTGCTGGAGCAGCTGTGTGCTACCTACAAGCTGTGCCACCCGG |
| CSF3R | XM_013123850.1 | 464-563 | CCAACTTTACCCTAAAAAGCTTCAGGAGCCGTGCTGACTGTAAGTACCAGAAGGACTCCATCACGGACTGTGTGCCTAAGGACAGGCAGAACACCTGCTC |
| CTLA4 | XM_005070583.1 | 234-333 | CAGTCAGATGACGGAGGTCTGTGCCACGACATTCACAGTGAAGAATAATTTGGGCTTCCTAGATGATCCCTTCTGTAGTGGTACCTTCAATGAAAGCAAA |
| CTNNB1 | XM_005082323.2 | 2223-2322 | ACAAGAAACGGCTCTCAGTAGAGCTGACCAGTTCTCTCTTCAGGACAGAGCCAATGGCTTGGAACGAGACTGCTGATCTTGGACTGGACATTGGTGCCCA |
| CTSC | XM_005085370.2 | 809-908 | CCATGGTGTCAATTATGTTAGCCCTGTTCGAAACCAAGAATCTTGTGGGAGCTGCTATTCATTTGCCTCTATGGGTATGCTAGAAGCAAGAATCCGCATA |
| CTSG | XM_005075353.1 | 444-543 | CCTGGTGAGCCGGAGCAGGCGAACCAATGTACTCCAGGAGGTGCAGCTAAGAGTGCAGAGGGACCAAAAGTGCAGCAATCGCTTCGGCACCTACAACAGC |
| CTSS | XM_013123783.1 | 555-654 | TTTCAGTACGTCATCGATAATGGTGGCATCGACTCAGATGCTTCCTATCCTTATAAAGCCACGGCTGAAAAATGTCACTATGACTCAAAAGGTCGCGCTG |
| CUL9 | XM_013115072.1 | 2667-2766 | CTGAGGGGTGCTCCTCTGCAGTGAGAAATGGTTTGCTTCTCCTCAACTTGCTTTTGTGTAACCACCACACTCTGGGAGACCAGATTATAACCCAGGAGTT |
| CX3CL1 | XM_005078617.2 | 162-261 | TCTATGCGCCCTGCTGCAGGGTCAACACCTCGGCGTGACGAAATGCAACATGACCTGCCACAAGATGACCTCACAAATCCCGGTGACTTTGCTCATCCAC |
| CX3CR1 | XM_013122171.1 | 1830-1929 | CCGTGTGTTTTCATTCGATGTAGTGAGCAGTGGGGGCCCTTGAAGTAGATAAGATGCAGTTCTAAAAATAGACTCCGCGTTGTCTCTTCCTTCCCAAAGG |
| CXCL10 | NM_001281344.1 | 289-388 | GTCTGAATCCAGAGTCTGAGGCCATCAAGAGTTTATTGAAAGCAGTTAGCCAAAGAAGGTCTAAAAGAGCCTCTTAACCAGAGAGAAGCCGATCACCGCA |
| CXCL11 | XM_005068143.1 | 91-190 | CTTTGCATAGGACCCTTAGCGAAAGCAGTCAAGATAGCAGACATTGAGACAGTCTCCGTATTTTACCCAAGCTACCGCTGTGGCAAAGTTGAAGTGATTG |
| CXCL12 | XM_005066089.2 | 369-468 | CCTCAACACTCCGAACTGTGCCCTTCAGATTGTCGCAAGGCTGAAGAGCAACAGCAGACAAGTGTGCATTGACCCGAAACTAAAGTGGATTCAGGAGTAC |
| CXCL13 | NM_001281341.1 | 155-254 | CGGAATGGTCTCAGGCGTTATCCATCCAAGCTTCATAGAACAAATTCAGGTCAGATATCGTGGGAATGGCTGCCCAAAAGATGAAGTCCTGATCTCCACT |
| CXCL2 | XM_005068086.2 | 61-160 | CCTATCCAGCGCCATGGCTCCAGCCACCCGTTCACTCCTCCGTGCCTCTCTGCTGTTGCTGCTGCTTCTGGCCACCAGCCGCCAGGCTACAGGGGCAGTT |
| CXCR1 | XM_005070504.1 | 549-648 | CCGCCAGGCATATAAACCATACGGCTCTGGAGCAATCTGCTACGAGGTCCTGGGTGGAGACACAACAAACTTTCGGATAATGTTGCGTGGCCTGTCCCAC |
| CXCR2 | XM_013113710.1 | 836-935 | ATGCATAACCATGTGGGTACTGTCAGTATTGGTGTCCCTGCCCATCTTAATTCTGCGTAACATCGTCAAGACAGGCTCTTCTACTCTAGTCTGCTATGAG |
| CXCR3 | XM_005081206.2 | 306-405 | CCTTCCTGCTTCATCTGGCTGTGGCTGATGCACTGCTGGTATTGACCCTCCCGCTGTGGGCAGTAGATGCTGCTGTTCAGTGGGTTTTCGGCTCTGGCCT |
| CXCR4 | XM_005079873.2 | 807-906 | GCTTTCTTTGCCTGCTGGCTACCATATTACATCGGGATCAGCATTGACTCCTTCATCCTCCTGGAGGTCATCAAGCAAGGATGCGATTTCGAGAGCCTGG |
| CXCR6 | XM_013122234.1 | 359-458 | CTCTCACTGGCATTACGGTGGACCGTTTCATTGTAGTGGTCCAGGCTACCAAGGTTTTTAACCAGCAGGCTAAGTGGAAGATCTGGGGCCAAATCACTTG |
| CYBB | XM_005080577.2 | 797-896 | CTCCTATGACTTGGAAATGGATAGTGGGTCCCATGTTCCTGTATCTCTGTGAGAGGCTGGTTCGGTTTTGGCGATCTCAACAGAAGGTGGTCATCACTAA |
| CYSLTR1 | XM_005085945.2 | 557-656 | ACATTACCTCTCCGTGTGGTCTATTATGTTCACAAAGGCAAGTGGCTCTTTGGTGACATTTTGTGCCGCCTCACCACCTATGCCTTGTATGTTAATCTCT |
| CYSLTR2 | XM_013114024.1 | 613-712 | CATCTGCTACCTCTTGATCATCCGGGCCTTGTTGAAGGTGGAGATTCCAGAGTCGGGTCTACGGGCTTCTCACAGGAAGGCGTTGATCACCATCGTCATT |
| DAXX | XM_013124321.1 | 834-933 | TCCCTGACTGGCCGGGTCATAGAGCAGCGAATCCCCTACCGCGGCACACGCTACCCCGAGGTCAACAGGAGCATTGAGCGGCTCATCAACAAGCCAGGGC |
| DDR2 | XM_005071424.2 | 2689-2788 | CAGGTTATTGAGAATACTGGAGAGTTCTTCCGAGACCAAGGGAGACAGATTTATCTCCCTCAACCAGCTATTTGCCCCGACTCTGTGTATAAGCTGATGC |
| DDX3X | NM_001281387.1 | 1197-1296 | GTACATTTTTCTGGCTGTAGGCAGAGTTGGGTCTACTTCAGAGAACATCACACAGAAAGTGGTTTGGGTGGAGGAAATGGACAAACGGTCATTTCTGCTT |
| DDX58 | XM_013119639.1 | 1008-1107 | AAGGGAAAGTGGTCTTTTTTGCTAACCAAATTCCTGTCTACGAACAGCAGGCAACTGTGTTCTCTCGCCATTTTGAAAGACTTGGGTACAGCGTAGCAGG |
| DECR1 | XM_005066815.2 | 631-730 | GTGGATCAGGCTTCGTAATGCCAAGTTCCTCAGCTAAATCAGGCGTGGAAGCCATGAATAAGTCTCTTGCAGCTGAATGGGGTAGATATGGAATGCGATT |
| DEFB1 | XM_013122443.1 | 135-234 | AGGTGCGGGTGTTCTCACAAGTCTTGGACGCAGAACGGACCAATACCGATGCCTCCAGCATGGGGGCTTCTGTCTCCGCTCCAGTTGCCCTTCCCATACC |
| DHX58 | XM_005087391.2 | 1367-1466 | GGAGTTCAGGGATGGTATTCTAAACCTGCTAGTGGCCACAAGTGTGGCAGAGGAGGGGCTGGATATTGCCCAGTGCAATGTGGTGGTGCGCTATGGGCTC |
| DNAJA1 | XM_013119633.1 | 484-583 | CACCAGCTCTCAGTGACCTTAGAAGACTTGTATAATGGTGCAACAAGAAAATTAGCTCTGCAAAAGAATGTGATTTGTGACAAGTGTGAAGGCCGAGGAG |
| DOCK9 | XM_013120095.1 | 2699-2798 | AGTGCCATGAGGAAGGATTGGAGAGCCACTTGAGGTCATATGTTAAGTTTGCCTATAAGGCCGAACCGTATATTGCGTCTGAATATAAGACGGTGCATGA |
| DPP4 | NM_001310571.1 | 896-995 | GTGATGTGGCATGGGTTACAGAAGAAAGGATTTCTTTACAGTGGCTCAGGAGGATTCAGAACTACTCTGTGATGGCTATCTGTGACTACAACAAGACTAA |
| DUSP4 | XM_005066620.2 | 729-828 | CGCGAGGACAGCACCGTGTCGCTGGTCGTGCAGGCGCTGCGCCGTAACGCCGAGCGCACCGACATCTGTCTGCTGAAAGGTGGCTATGAGAGGTTTTCTT |
| DYSF | XM_005071078.2 | 2747-2846 | TGTGCAGATCCGGGTCAAGCTCTGGTTTGGGCTTTCTGTGGATGAGAAAGAATTCAACCAGTTTGCCGAGGGGAAGCTCTCTGTCTTTGCCGAAACGTAT |
| EBI3 | XM_005081393.2 | 506-605 | ACTTCCGCCAGGTGGGACCTATTGAAGCCACGACCTTCACCCTCAGGACCACAAAGCCCCACGCCAAGTACTGCGTCCAGGTGTCAGCCCAGGACCTCAC |
| EDNRB | XM_013113501.1 | 587-686 | AGGCGTCTGTGGGAATCACTGTGCTAAGTCTATGTGCTCTAAGTATTGACAGATATCGAGCTGTTGCTTCTTGGAGTCGAATTAAAGGAATTGGGGTTCC |
| EEF1A1 | XM_005073705.2 | 562-661 | GTGTGAAACAGCTAATTGTTGGTGTCAACAAAATGGATTCCACCGAGCCACCCTACAGCCAGAAGAGATATGAGGAAATTGTTAAGGAAGTCAGCACCTA |
| EGR1 | XM_005065288.2 | 1777-1876 | CTAACTCCTTCAGCACCTCCGCTGGGCTTTCGGACATGGCAGCAACCTTTTCTCCCAGGACAATTGAAATTTGCTAAAGGGGAAGCAAGCAAAGGGGAGG |
| EGR2 | XM_005070806.2 | 528-627 | GGAGAGAAGAGGCCCTTGGATCTCCCGTATCCCAGTAGCTTTGCTCCCATCTCCGCGCCTAGAAACCAGACCTTCACTTACATGGGCAAATTCTCCATTG |
| eIF2a | XM_005077906.2 | 221-320 | ATATAATCAGTGTCACTAACAAGGGACTACTGCACTCCTTCGACGTCCCAAAAGCAGTTTGCCTTGAATTCTCGCCAAACAACACTGTCCTGGCAACGTG |
| Eif2ak2 | NM_001281946.1 | 1374-1473 | TTCACGGACCTAAGAGATGGCATCTTCCATGACGACATATTTGGCAGCAAGGAAAAAAGGCTTCTAACGAAATTACTCTCGAAGAAACCCACGGATCGAC |
| ELK1 | XM_005085710.2 | 1979-2078 | CCTGGCAATATCTGAGTCCAGGCAGCACTTGTATGATTTAATGGTTTGGGAGTCGTAGCCAGAGAAGAATCACAGTGTTAAAATAGGAATTGCTACCTCC |
| ENPP4 | XM_005072306.2 | 994-1093 | GATGACCTTGTTGGCGATCTTGTTCAAAAGCTCAAGGTGTTAGGAATGTGGGACAACCTTAATGTGATCATTACAAGTGACCACGGGATGACTCAGTGTT |
| ENTPD1 | XM_013118072.1 | 1243-1342 | ATTTTTTGCTCAAAGCCTTGGGAGGAGGTGAAAACCGCTCACCCTACAGTAAAGGAGAAGTACCTGAATGAGTACTGCTTCTCGGGTGCTTACATCCTTA |
| EOMES | XM_005082356.2 | 1391-1490 | CTGGAGGTCGGTACGGCGTTCAAAACTTCTTCCCGGAGCCTTTTGTCAACACTTTACCTCAAGCCCGATATTACAATGGTGAGAGAACCGTGCCACAGAC |
| ETS1 | XM_005084432.2 | 3256-3355 | ATCTCCAGGCTGGCCCTTTTGGGGATTTCGCTAAGAAAATGAAAATTGTAACTACCTTGTATCATCTTTGTGAGATTTGATGGCTGACTCCCAGATTCCC |
| EWSR1 | XM_013112480.1 | 815-914 | TGACTTTGGATGATCTGGCAGACTTCTTTAAGCAGTGTGGGGTTGTCAAGTTTCTGTTGTGGTGGACTTTGTGCAGGGGCTGTGGAAACCAGAACTTCGC |
| F11R | XM_005087784.2 | 599-698 | TGGGATAGATATGCTTACGGACTCCAAGAAAACCCGTGCCTTCATCAATTCTTCATACACGGTTGATCCAAAGTCGGGGGACCTGATCTTTGATCCTGTA |
| F13A1 | XM_005066385.2 | 1721-1820 | TCTTGGCCTATCTTTCGGGCAACATCACGTTCTACACCGGGGTCACCAAGGAAGAATTCAAGAAGGAGGCATTTGAAATGACACTGGAGCCCTTGTCCTT |
| FADD | XM_005064118.2 | 325-424 | GGCCTTTGACATCGTGTGTGACAATGTGGGGAGAGAATGGAAGAGACTGGCCCGCCAGCTGAAAGTGTCTGAGGTCAAAATTGATGGGATTGAGGCGAAG |
| FAS | XM_013112078.1 | 612-711 | TGACCCTGAATCTGGCATCTCAAATCCTGAAAACAAGCCAATGATTTTCCCAGACGTTGACTTGAGTAAATACATCCCGGATATTGCTGAACGCATGGCA |
| Fascin | XM_005079958.2 | 1073-1172 | CAAGAATGCCAGCTGCTACTTTGACATCGAGTGGTGTGACCGTCGCATCACTCTGAGAGCGTCCAATGGCAAGTTTGTAACTGCCAAGAAAAACGGTCAG |
| FASLG | XM_005071339.2 | 587-686 | GGGATCATTCTTGCAACAATCAGCCCCTAAGTCACAAGGTCTACATGAGGAACTCTAAGTATCTTGGGGATATGGTGCTAATGGAGGAGAAGAAATTGAA |
| FCAR | XM_013123673.1 | 92-191 | CTGTGAGGATCATCTGCCAGGGAACACCTGAAGCCTTCCTGTATCAACTGTCCCTGATGAAAAACTCCACACCCACAGTTATAGAGAAGAAACTGGGATT |
| FCER1A | XM_005078252.1 | 356-455 | CTGATATGGTATTAGACAATGAGTCCTTCGACATCAGATGCTATGGCTGGAAGAACTGGAGTCTCCACAAGGTGACCTACTATAAGAATGACTTCGCTTT |
| FCER1G | XM_005087775.2 | 345-444 | AACACACGGCCTTGGTTGCATTCTGTTTCTAGTTCTCTCCTGGCCCTCATGGTCCTTGTCACTATGCTACCTCCTTGATTTTGATGCTGGCTCACCTAAC |
| FCGR1A | XM_013123787.1 | 366-465 | GACTTTGAGGTGTCACGGCTGGCAGAGTAAGATGGTGTACAACGTGGTTTTCTATCGAGATGGAAAATACTTTCACTCTTCTCAAGACTCTGAGGTCACC |
| FCGR2B | XM_005071437.2 | 1002-1101 | TTCACTTCTCAAGCATCCTGAAGCTCCGGATGAAGATGCAGAGCATGATTACCAGAACCACATTTAATCTTCCTTGTCTTGACTTGGGATTGGGAAAAGC |
| FCGRT | XM_005084733.2 | 535-634 | CCCAACAATTCTTCATTACCCACGGCTGTGTTCGCCCTCAATGGCGAGACCTTTATGGAGTTCAACCCAAGCATTGGCAACTGGAGTGGGGAGTGGCCTG |
| FEZ1 | XM_005085853.2 | 1523-1622 | TTAAACATGAGCAACCGCGCACGCGCGGGGTCCTTTGCTGTTGGCTTCTAGTGCTAGTAATCATTGGATGCATGATCGGGCGCAGGACGCTGCTTCGTCT |
| FGA | XM_005076166.2 | 696-795 | GACTTGCTTCCTTCAAAAGACAGGCAGTACTTGCCAGCAATAAAAATGTCTCCAGTTCCCGACTTGATTCCTGGAAGTTTCAAGAGCCAACTTCAGGAGG |
| FKBP5 | XM_005084894.2 | 2022-2121 | ATTAGAGTGAATGCGTGAGGAGTAGGGAGCTGTTGACGGCTTGTTTCCTGCTGTGCCTCCCTTTGAAGAAGGTTAGCTCTCGATTCCCGGCCTCTCTGTG |
| FLT1 | XM_005083180.2 | 1035-1134 | GAGTTACCCTGGTAAAGCAACTAAGAGAGCTTCTATAAGGCAACGGATTGACCAAAGCAATCCCCACAACAATGTGTTCCACAGCGTTCTGAGGATCAAT |
| FLT3LG | XM_005084829.2 | 230-329 | CCCATCTCCTCCAACTTCCACGTGAGGATCAGCGAGTTGACTGACTACCTGCTTAAAGATTACCCAGTCACTGTGGCCATCAATCTTCAGGACGAGAAAC |
| FN1 | XM_005070516.2 | 5122-5221 | CAATTATCGAACAGAAATCGACAAGCCATCCCAAATGCAAGTGACTGATGTTCAGGACAACAGCATTAGTGTCAGGTGGCTGCCTTCAAGTTCTCCTGTG |
| FOS | XM_005086369.2 | 1451-1550 | CTCTGGACTCAAGTCCTCACCTCTTCCGGAGATGTAGCAAAAAAACAAAAAAACAAAAAACCGCATGGAGTGTATTGTTCCTAGTGACACCTGAGAGTTG |
| FOSB | XM_005086303.2 | 1408-1507 | GACGGCTTCTCTCTTTACACACAGTGAAGTTCAAGTCCTCGGCGACCCCTTCCCCGTTGTTAGCCCTTCGTACACTTCCTCGTTTGTCCTCACCTGCCCG |
| FOXJ1 | XM_005069887.2 | 1310-1409 | GGGCCTCCGGCGGAGCAGGCTGCCGACAGCCTGGACTTCGATGAGACCTTTCTGGCCACGTCCTTCCTCCAGCATCCCTGGGATGAGAGCGGCAGTGCCT |
| FOXP3 | XM_005085088.2 | 1149-1248 | CCGCCACAACCTGAGTTTGCACAAGTGCTTTGTGCGGGTGGAGAGTGAGAAGGGAGCGGTGTGGACCGTAGATGAGTTTGAGTTTCGCAAGAAGAGGAGC |
| FPGS | XM_005087539.2 | 328-427 | GGGACCAAAGGAAAGGGCTCCACCTGCGCCTTCACGGAACGAATCCTGAGGAATTACGGCCTGAAGACAGGCTTCTTTAGCTCTCCCCACCTGGTGCAGG |
| FPR2 | XM_005086647.2 | 441-540 | TGGACCCTGGATTCTTGCTCTCATCCTCACATTGCCCATTTTCCTCTTCTTAACTACAGTTAGAGTTCCAGGAGGGCATGTGTACTGTACATTCAGCTTT |
| FXYD2 | XM_013113008.1 | 177-276 | GGGGGCAGAGGATCCCTTCCACTATGATTATGAGACCGTCCGCAAAGGGGGCCTGATCTTCGCGGGCCTGGCCTTCGTCGTGGGGCTCCTCATACTCCTC |
| FYN | XM_005073256.2 | 1662-1761 | TTGCTGCGGGAATGGCTTACATCGAGCGCATGAACTATATCCACAGAGATCTGCGATCAGCAAACATCCTAGTGGGGAATGGACTGATTTGCAAGATTGC |
| GAPDH | XM_013124485.1 | 171-270 | TACCCATGGCAGTTCAAAGGCACAGTCAAGGCTGAGAATGGGAAGCTTGTCATCAATGGGAAGGCCAACACCATCTTCCAGGTGTGAGATCCCACCAACA |
| GATA3 | XM_013114620.1 | 2179-2278 | CAGTGAGAAGTCTCACCAATTGCCGGTGAGGATTTAAGAATGTCATGTCTAGGCCTCCATGCTCTGTGAACCAGTCCCTGTAATTGTTGTTTGTATGTAT |
| GBP1 | XM_005081952.1 | 1818-1917 | GGAGAAAACACAGGCCCAGATGCAAGAAATGCACCAAAAGAATGAACAATTGCTGCAAGTGAAAAAGAGTCACAAGGAGCGTGTGAAAGAGTTGACTGAG |
| GFI1 | XM_005077391.2 | 391-490 | CCTTCTCCCTCGGTTTCTCCAGGCTCTGAGAAGTCGTTATGCCGCTCTCTGGACGAAGCTCAGCCCTACACGCTGCCTTTCAAGCCCTATGCGTGGAGCG |
| GNAQ | XM_005063781.2 | 623-722 | GACAGACGACGAGAGTATCAGTTATCCGACTCTACCAAATACTATCTGAATGACTTGGATCGTGTAGCCGACCCTTCCTATCTGCCTACACAACAAGATG |
| GNAS | NM_001281941.1 | 540-639 | AGCTGGCCAACCCTGAGAATCAGTTCCGTGTGGACTACATTCTGAGCGTGATGAACGTGCCCAACTTCGACTTCCCACCTGAATTCTACGAGCACGCCAA |
| GNB1 | XM_013126579.1 | 588-687 | TTCACTTATTGCTGAAACCAAGAGCACAACTCCCATTCAGAGAAGGATCTCTGTGCTGTAAACTAAAACAAATCGTGCATTCCTTCCGGGGCCATTGTCT |
| GNGT1 | XM_013122668.1 | 60-159 | CCAGCTCAAGAAAGAAGTGACCCTGGAAAGAATGATGGTTTCCAAGTGTTGTGAAGAAGTAAGAGATTATATTGAAGAAAGATCTGGAGAGGATCCCCTA |
| GP1BB | XM_005077595.2 | 259-358 | CTCGCTGCCCGCTGCCTTCCCTCCCGACACCACCGAACTGGTGCTGACCGGCAATAACTTGACGGCACTGCCGCCCGGGCTTCTGGATTCACTGCCTGCG |
| GPI | XM_005081777.2 | 1107-1206 | CCATGCTGCCCTATGACCAGTACATGCACCGCTTTGCTGCTTACTTCCAGCAGGGCGACATGGAATCCAATGGCAAATACGTCACCAAGTCGGGTGCCCG |
| GPR183 | XM_013120113.1 | 678-777 | GCCTGCTGGGCTACGTGCTGCCTCTCATAGTCATCCTCATCTGCTACTCTCAAATCTGCTGCAAGCTCTTTAAGACGGCCAGGCAAAACCCGCTGGCTGA |
| GRB2 | XM_005069919.2 | 641-740 | GGGCCGGGAAGTATTTCCTGTGGGTGGTGAAGTTTAATTCTTTGAATGAGCTGGTGGATTACCACAGATCGACATCCGTGTCCAGGAACCAGCAAATATT |
| GTF3C1 | XM_005064446.2 | 3850-3949 | TTGCTAATGCTCTGCCGAATTGCCAGCAATGTCCTCAATACCAAGGTGAAAGGACCATTTGTCACCTGGCAGGTTGTGCGAGACATTTTGCATGCTACTT |
| GZMA | XM_005065473.2 | 152-251 | AAAACATCTGTGCTGGCACTTTGATTGCAAAAGACTGGGTGTTGACTGCTGCCCATTGCAACCCGGAAAAGGGTTCTCAAGTCATCCTTGGAGCTCACAC |
| GZMB | XM_005075349.1 | 255-354 | TTATCTTGAGATCATGAATCGGGATACTCGAAGTAGGTGCGGCGGCTTCCTCATACGAGAGGACTTTGTGCTGACGGCTGCTCACTGTTCAGGGAGCTCA |
| GZMH | XM_013124876.1 | 65-164 | ACAACCAAGTCGATGTGGTGGTTTCCTGGTGGAGAAAGACATCGTAATGACAGCAGCTCACTGTTATTCAAGGAAAATAATTGTAACCTTGGGTGCTCAC |
| GZMK | XM_005065528.1 | 231-330 | CATGGTGGTTTTAGGAGCACATTCTCTTTCAAAGAATGAACCCACGAAACAGACGATTAAGATTAAAAGACTCATCCCATTCTCAAGGTCTCCGTCAGGT |
| GZMM | XM_013122967.1 | 990-1089 | GTGCTGGGCCTGCACCACCTCCATGACTTGCGAGACCCGGGCCTCACCTTCTACATCAAGGCGGCCATCAAGCACCCTGGTTACAACCACAACCTTGAAA |
| HAMP | XM_005081868.2 | 237-336 | AAGCGAACGAGGCGAGACAGCCACTTCCCATTCTGCACTTTCTGCTGTTACTGCTGCGGCAATTCTGACTGTGGCTTCTGCTGCAAAACATAGAGCCGCC |
| HAT | XM_013111989.1 | 335-434 | GTAACTCTGGAAGTTTGGAAATAACCCCTTCAAATGAGATAACATCACTCACTGACCAAGATACAGAAAATTTTTTGACTCAAGAATGTGGAGCCCGTCC |
| HAVCR2 | XM_005071890.2 | 294-393 | TAAGGGGGAGTTTCCTCAAAGGAGATGTGTCCCTGACCATAGAGAATGTGACTGTAGATGACCGTGGGACCTACTGTTGCAGGGTAGAATTCCCTGGTCT |
| HDAC4 | XM_005082194.2 | 2939-3038 | ACGGTGGTCATGCCAATTGCAAATGAGTTTGCCCCAGATGTGGTACTGGTGTCATCAGGCTTCGATGCTGTGGAGGGCCACCCCACACCTCTTGGAGGGT |
| HFE | XM_005066463.2 | 1034-1133 | ATGGGACCTATCAGGCCCAGATGACCTTGGCTGTAGCCCCTGGTGATGAGACAAGGTTCACCTGTCGAGTGGAGCACCCAGGCTTGGATCATCCCCTCAC |
| HIF1A | XM_013115411.1 | 2178-2277 | TTGGAACTTTATTGCAGCAGCCAGGTGACCGTGCTCCTACTACATCACTTTCTTGGAAACGTGTGAAAGGTTGCAAATCCAGTGAACAGAATGGAATGGA |
| HLA-DMA | XM_013125805.1 | 767-866 | CTGGGCATCGTCATGGGCATTGCGTTCTTCATCTGGTCCCAGAAGCCATGTTCGGTTGACTGAGTCTTCTGTGACTAGCGCTTGGAACAGCACAGGCCTT |
| HLA-DMB | XM_013125801.1 | 367-466 | CCGGAGGAAGGAAAAATAGTTCCCTGTGAATTTGGGGTGCTGTACAACTTGGCTGTATTCATTTCAGATAACCTCAATGAAAATAAGGCCCTGCTCGAGC |
| HLA-DOB | XM_013125800.1 | 443-542 | TCTACCCTGGGGACATAAAAGTCAGGTGGTTTCGGAATGGACAGGAGGAGAGGTCTGGGGTCGCGTCCACTGGCCTCATTAGGAACGGAGACTGGACTTT |
| HLA-DPA1 | XM_005086891.2 | 150-249 | AGGATCTGGTGCCATCAAAGCGGACCACGTAGCCATGTATGACATATTTGCACAGACACAAAAGCCCTCGGGTGACTGCATGTACGAGTTTGACGGCGAT |
| HLA-DPB1 | XM_005086892.2 | 664-763 | ATGCTGACGGGAGTGTGTGGCCTGGTACTCGGACTCATCTTCCTGGCGGTGGGCGTCGCCATGCACCTGAGGTGTAAGAAGGGTTCCACCTGCCGTGGTG |
| HLA-DRB1 | XM_005088612.2 | 241-340 | AGCCAGAAGGAGTACCTGGAGCAGAAGCGGGCCAACATAGACGTGTACTGCAGACACAACTACAGGATTATGCACAGCTTCACTGTGCAGCGGAGAGTTG |
| HMGB1 | XM_013122822.1 | 228-327 | ACAGACACAGAAAATCATCTAAACATGGGCAAAGGAGATCCTAAGAAGCCGAGAGGCAAAATGTCCTCATATGCATTCTTTGTGCAAACTTGCCGGGAGG |
| HMGB2 | XM_005075154.2 | 504-603 | GAGATACTGCAAAAAAGTTGGGTGAGATGTGGTCTGAACAGTCTGCCAAAGATAAACAACCCTATGAGCAGAGAGCAGCCAAACTAAAGGAGAAGTATGA |
| HMGN1 | XM_005073788.2 | 178-277 | GGCGAAGGCGGAGCCCAAGCGCCGCTCCGCGAGGCTGTCAGCCAAGCCCGCCCCTGCTAAGGTGGACGCGAAGCCGAAAAAGGCCGCGGGAAAGGATAAA |
| HP | NM_001281379.1 | 933-1032 | GCAGGACACCTGGTATGCAGCTGGGATCCTGAGCTTTGATAAGAGCTGTTCTGTAGCTGAGTATGGCGTGTACGTGAAGGTGAACTCCTTCCTGGACTGG |
| HPRT | XM_005085546.2 | 233-332 | TGTTTATTCCTCATGGAGTGATTATGGACAGGACTGAAAGACTTGCTCGAGATGTCATGAAAGAGATGGGAGGCCATCACATTGTGGCCCTCTGTGTGCT |
| HRAS | XM_005064218.2 | 151-250 | CCTGACCATCCAGCTGATCCAGAACCATTTTGTGGACGAGTATGACCCCACCATAGAGGATTCCTACCGGAAACAGGTGGTCATTGATGGGGAGACATGT |
| HSD11B1 | NM_001281353.1 | 528-627 | CCATCCTCTGGTTGCTTCCTACTCTGCATCTAAGTTTGCTCTGGATGGGTTCTTCTCCTCCCTTAGAAGAGAACACGGAGTAACCAATGTCAACGTGTCC |
| HSH2D | XM_005087080.2 | 1081-1180 | GGTTACAGGGCTGAATTCTGCAGTTGCCAACAGAGGGACCTGATTGTTTTAATAATAATAGAATTACCACGTTAGGGACTCGGATGAGATGGCCCATTGG |
| HSPA1L | XM_005086832.2 | 1852-1951 | CAGGGTGCTGGGGTGCCTCTAGGGGAATTTTTATTCATCTTGGAACGTCACTATGACTCTTGAGCTGACTAGACTTGAGCCTAAGTCACCGTCCTTTGGG |
| HSPB1 | XM_005080371.2 | 616-715 | CTTGTCCCCTGAGGGCACACTTACTGTGGAGGCTCCGCTGCCCAAAACAGCCACACAATCATCGGAGATCACCATTCCGGTCACTTTCGAGGCCCGCGCC |
| HSPB2 | XM_005069515.2 | 353-452 | TCCCGAGAGTTCTGTCGCACCTATGTCCTGCCAGCAGATGTAGACCCCTGGCGGGTCCGAGCTGCCCTCTCCCATGATGGTATCCTTAACTTGGAGGCGC |
| ICAM1 | XM_005078593.2 | 1012-1111 | CTCAGTGGCCCAAAGTAGAGCTGGATCGTGGAGAAACCTGGAAGCTGTTTGAATTGAGTGACATCGAAGAAGACAGCAGCCCACTGTGCTTTGAGAACTG |
| ICAM2 | XM_005070009.2 | 349-448 | AACAGTTCTTAGTTTCAAACACCACCAACGACACGAAACTCATCTGCCACTTCACCTGTGCGGAGCAACAGCTCTCGGAGCATCTCAATATCACAGTATT |
| ICAM4 | XM_005078564.2 | 729-828 | CTGCCATGCACGCCTCAATCTCGGTGGGCTAATAGTGCGCAGCAGTTCTGCACCTGTTATGCTGACGGTCCTCGCTTTGAGCCCAGCCTCCAAAGCCTTG |
| ICAM5 | XM_005078563.2 | 2417-2516 | AGGGTGCCAGCGAGGGGGCGGTATTACCCCTGGTATCCTCAAACCCTGGTCCTAGAAACTCTATGACCTCTAGTAAACTTTCCCCGGGTGTCTACCTCTG |
| ICOS | XM_005070650.1 | 225-324 | CAAGGAAACCAAGACCTGTTCGTATCAGCTGTCCAACGGCAGTGTCTCTTTTTTCCTAAACAACCCGGACAGTTCTCAGAGCAGCTATTACTTCTGTAGC |
| ICOSLG | XM_013113837.1 | 158-257 | CCCAGCCTTGGGAGCCAGCCAGCCAGTTTGGAAGAAGCACCATCTCATTTGTCTCTCAGGAGGGTTCTTTTCCAGTCCCAGTCTGCTCTTGCTGCTCATC |
| IDO1 | XM_005066560.2 | 551-650 | CCACTGTATTCAGTGCAGTACAGAATCAAGACCAGAAAACGTTGGCAAGGGCATTGGGTGATATAGCTACCAGTCTGGAGAATGCCAGGGATTATTTTAG |
| IFI27 | XM_013112166.1 | 218-317 | GACTTTCCACAACATCCAACATCATCCTGGGCTCTGTTGGGGCAGTTGTTGGGGCATCTGTCGAGGAAGGTGTTGAAGTCTTGCTTGAATTTATTAGACG |
| IFI27L2 | XM_013112167.1 | 249-348 | TGTTGGGGCTGGCTCTGTTGGGGCAGCTCTTCTGGCCTTGCTTTGAACTTACTGATGACACTTCTGCAGTTGAACCTACTGCCTATAAGGAATATCCAGA |
| IFI35 | XM_005070131.2 | 647-746 | GCCCGGGAGATGCTGCAAGGGACGGTCATGCTAGGTTTTGCTGATGAAAAGGTGGCTCAGAACCTATGCCAGATTGGCCAGTTCAGAGTGCCACTGGGCC |
| IFI44 | XM_013123197.1 | 853-952 | CATCTTAAAAGGCCACACTCCTGACAGATACCAGTTCAATTCCATGAAACCAATCACACCAAATCATCCAAACTACATTCATGACTCACTGCTGAAGGAC |
| IFIH1 | XM_013112600.1 | 2108-2207 | CGCGTCAGAGTACCTATGCACTTTCCCAGTGGATTACGGAAAATGAAAAGTTTGCAGAGGTTGGAGTCAAAGCACATCATCTGATTGGAGCAGGACACAG |
| IFIT1 | XM_013110344.1 | 288-387 | AGCAGAGGCAAATTTCCAGAAGGTTCTGAACATGACTCACAGTGATTATCACATAGAGCAGGATGTGCATTTCCGCTATGGCTGTTACCAGCAATTTCAT |
| IFIT2 | XM_013112267.1 | 649-748 | GACTCTCTGAAGCAAGCCATTAGACTGTCTCCCAACAGTCCTTATGTTAAAGTTCTCTTGGCACTGAAGCTTGAGATGATGCATGAAAACCAAGGAAAGG |
| IFIT3 | XM_005063204.2 | 492-591 | ACGCCTAAAGTGTGGAAGGAACGAAAGGGCTAAAATGTGTTTCTTAAAGGCCCTAGAAGAGAAGCCTAGTGATCCAGAATGTTCGTCTGGGCTTGCAATT |
| IFITM1 | XM_013116372.1 | 403-502 | CCCACATCCCAACTTCTGCAACAACCACCATAATCAACATGCCTGGTGAGGTCTCCCTGCCTGATCATGTGGTCTGGTCCATGTTCAATACACTCTTCAT |
| IFITM2 | XM_013119372.1 | 818-917 | AACTGCCAAGCGACTGAACATCGCTGCTGTGGTCTTCTCCATCATCTCGTTCATTGTGCTTATCATTATAGTCAGCCAACAAAGAGGATAGGATGGGCGC |
| IFITM3 | XM_005064225.2 | 569-668 | GTGGCTGAGCTGGGGGCATCCCACGGCTCAACCTCTGTCAGAACCACTGTGATCAACATGCCCAGAGATGTTGCTGTGCCTGACCATGTGGTCTGGTCTC |
| IFNA13 | XM_005074336.1 | 126-225 | ACAAATGAGGAGACTCTCTCCTCTCTCCTGCCTGAAGGACAGAAAGGACTTTGCATTTCCTCTGGAGAAGGTGGATGTCCAGCATATCCAGAAGGCTCAA |
| IFNAR1 | XM_013116152.1 | 1206-1305 | CGTTTTTTGGGACAAGACTTCCAATACTAAGAGAAGAGTGGTGAAGGAGAGCCCAGAGTTTACCATCGAGAACTTGCAGCCACAGACTGTGTACTGCGTG |
| IFNAR2 | XM_013116154.1 | 601-700 | TATACGTTTTGGTCCACAATCATGAGCAAACTGGAAGACCCGAAGGCTTTGGAGAACTGTACAAATATCACGGAATCCTCTTGCGACGTGACAGATGAGT |
| IFNG | NM_001281631.1 | 417-516 | TTCAGGTTCAGCGGAAAGCTGTCAATGAACTCATCAGTGTGATGCCTCACCTGTCACGAAAACTTAGCCTAAGGAAACGGAAAAGAAGTCGGTGCTGTTT |
| IFNGR1 | XM_005065730.2 | 657-756 | GACACCCTGTGCCAATTCAACATTTCCATGTCCACACTGGATTCCAGATACTGTGTCTCAGTGGACGGATTCTCAGAGTACTGGGGCATTACAACAGAAA |
| IFNGR2 | XM_013116151.1 | 814-913 | CCCTTCAATGTGACGAAGGAAGCGGCTTTTGAGTACCGCGTCCATTACTGGGAAAAAACAGGAGCCCCACAGGCAAGAACACGTCACTTCTTTGTCTTTT |
| IGF2R | XM_013127266.1 | 4643-4742 | ACATTGGCCGAGTCACAGGTCCTCCAATATTCAACCCTGTGGCGAATGAAGTCTACTTGAACTTTGAAAGCAGTACTCCTTGTTTGGCGGACAAGTGCCT |
| IGJ | XM_005068171.2 | 381-480 | GCACTATCTGCAGTGAAGACAGTGTTGTTCCGGAGACCTGCTATATGTATAACAGAAATAAGTGCTACACAGCTATGGTCCCACTTACTTATCGTGGTGA |
| IKBKB | XM_005066537.2 | 1487-1586 | TTCGGAACAACAGCTGCCTTTCCAAGATGAAGAATGTCATGGCCTCCATGGCGCAGCAGCTCAAGGCCAAGTTGGATTTCTTTAAAACCAGCATCCAGAT |
| IKBKE | XM_005079851.2 | 1767-1866 | CTGAGCTGCCTGGGCAGAGAGTTTCTGAAGAACCAGAATCAGATCCACGATGACAGCCGAAGCATCCAGAAGATTCAGTGTTGTTTGGACAAGATGCACT |
| IKBKG | XM_005086898.2 | 3471-3570 | TTGTTAGACAGACTTGGTCTATAGGGTGATGGTGATCATTAGGCTTCAGAATTTGGAGGTCAGGCCCTTGTAGGTTGGCTGTAGCGGGAGAAAGTTGTTG |
| IKZF1 | XM_013122687.1 | 1919-2018 | TGTTAGAGACAGGATTGCATTGCATCGGGAGCATTCAGAACATCCATGCGTCTACACGGTTTTGTTGTCTGCTAGCTGAGATTTCCCTGACCAGTGGCTT |
| IKZF2 | XM_005070532.2 | 1431-1530 | CTGAAGGACATCTATAAGGTTTTCAATGGAGAAGGAGAACAGATAAGGGCCTTCAAGTGTGAGCACTGCCGAGTCCTTTTCCTAGACCATGTCATGTACA |
| IKZF3 | XM_013117634.1 | 756-855 | GCTCATTCTGGACAGATTAGCAAGCAATGTGGCAAAACGAAAAAGCTCAATGCCTCAGAAATTCATTGGTGAGAAGCGTCACTGCTTTGATGTCAACTAC |
| IL10 | XM_005079860.1 | 188-287 | ACATACTACTCACTGACTCCTTACTGCAGGACTTTAAGGGTTACTTGGGTTGCCAAACCTTATCAGAAATGATCCAGTTTTACCTGGTAGAAGTGATGCC |
| IL10RA | XM_005069462.2 | 774-873 | GAGTTTTGTATCAAGGTGTATCCCTTGGTGGAAACCCGAAATAACAGAGGAGAATGGTCAGAGGAGCAGTGTTTGCGAATCACAGAGCAATATATCACCT |
| IL10RB | XM_013116153.1 | 296-395 | CTTGGCCTGGCATTTATCAAAACCTGCCAGTCTCCTGTGGAGGCACCCTTCCTGTTGGCATCGCAGAGGTGGTTATCATCGTTGAGGAAGACAGGACTGG |
| IL11 | XM_005084121.2 | 268-367 | CTTCCAGGTGTGCTGACAAGACTTCGAGTAGACTTGATGTCCTACCTCCGGCATGTACAGTGGTTGCGCCGGGCAGGTGGACCTTCCCTAAAGACCCTGG |
| IL11RA | XM_005078821.2 | 1151-1250 | GCTTGATCACAGAGATCCCTTGGAGCAAGTGGCTGTGTTAGCATCTTTGGGAATCTTCTCTTTTCTTGGCCTGGCTGTTGGAGCCTTGGCACTGGGGCTT |
| IL12A | NM_001281367.1 | 647-746 | GCAGAAGCGGATCCCTACAAAGTGAAAATGAAGCTCTGCATTCTACTGCAGGCTTTCAGCATCCGTGCTGTGACAATCCACAGAGTGATGAGCTACCTGA |
| IL12B | NM_001281689.1 | 349-448 | TCACAAGAAGGAAAATGGAATTTGGTCCACTGATATTTTAAAGGACCAGAAAGATCCTAAAAATAAGACCTTCCTGAAATGCGAGGCAGCAAATTACTCT |
| IL12RB1 | XM_013125271.1 | 1306-1405 | TGTTACCGCATCACCGTCTTCGCCTCTAAGGATCCAGAGAACCCGGTGCTGTGGTACACGCTCCTGTCCGGTTACTACTTTGGGGGTAACGCCTCGGTGG |
| IL12RB2 | XM_013117753.1 | 965-1064 | GCCTGTTGGGACATTAGACATCTGGTACATGAAACAGAACATCAGCTATAACAGACAACAGATCTCTCTTTTCTGGAAGAATCTGAGTCCATCAGAGGCA |
| IL13 | XM_005067910.2 | 208-307 | GCCCTGGATTCCCTGACCAACATCTCTAGTTGCAGACCCATCTACAAGACCCAGAGGATATTGAACGGGCTCTGCGTCCGAAGGGCATCAGCTGGGGTTT |
| IL13RA1 | XM_013122813.1 | 1144-1243 | GAATTTGATAGAAACATGGAGGGTACAAGTTGTTTCGTGCTCCCTGGTGTTCTTCCTGACAATGTCTACACAGTCAGAGTAAGAGTCAAAACAAACAGGC |
| IL15 | XM_005077725.2 | 685-784 | CGAGCGTCTTAATGAAACAGTAAGAAACGTGATCTTCCTAGCAAACAGCAGTCTGTCTTCTAACAAGAATATAACAGAACATGGCTGCAAGGAGTGTGAG |
| IL16 | XM_005077851.2 | 3447-3546 | AGTGAGAGTTCTAGATGAGGCGACATTGAAGCAACTAGACAGCATTCATGTCACCATCTTACACAAGGAAGAAGGTGCTGGCCTTGGATTCAGCTTGGCA |
| IL17A | XM_005072283.2 | 134-233 | AGAATGTGAAGGTCAACCTGAATGTCCTTAACTCTCTTAGTCCGAAAGTGAGTTCCAGAAGGCCCTCAGACTACCTCAACAGATCCACTTCGCCCTGGAC |
| IL17B | XM_013116760.1 | 363-462 | GTGGGAACCTGTAACATCAAGGAGAAGCTATACCATGTTGCCCTGGATTTCCAGTATGACATGCCTGCTGCTGCATCCTTCTTCCTGGATAAGAATTATG |
| IL17F | XM_013115051.1 | 269-368 | ACCGGTTCCCCTCAGAGATTGCTGAGGCTCAGTGCAGATATTCAGGCTGCATCAATGCACAGGGACAGGAAGACAGCTCCATGAACTCCGTTGCCATCCA |
| IL17RA | XM_013110513.1 | 1532-1631 | TGGGGACCTTTTCACGGCAGCCATGAACGTGATCCTGCCAGACTTCAAGAGGCCAGCCTGCTTTGGCACCTACATTGTTTGCTACTTCAGTGGCATCAGT |
| IL18 | XM_005069293.2 | 765-864 | ATATATGTATAGAGATTCTAATGCAAAGGGACTGCCTGTAACCCTCTCTGTGGAGAATCAAAAAATGTTTACTCTCTCCTGTAAGGACAAAGTCATCTCC |
| IL18R1 | XM_005075366.2 | 590-689 | TTGTAAGGAAATAGTCAAACCTCCAATGATCCCGAAGGACGCTGAATTTGGAGATGAGGGCTATTACACCTGTGTGTTTTCTCTCCACCACAATGGGAAG |
| IL18RAP | XM_005075365.2 | 596-695 | ATAAGAACAGCATCATTAAGTTGGGTGCCATTTATGACTCTTACCAGGGCTTGTATGTGTGCGATTACACTCAGTCGGATAATGTGAGCTCCTGGACAGT |
| IL19 | XM_005079888.1 | 296-395 | AAGTCATGAGGCAAATCAGCAGCATTGCCAACTCCTTCCTCTACATGCAGAAAACCCTGGAGCAATGTCAGGTGCATAATCAGTGTCAATGTAGTCAGGA |
| IL1A | XM_005068609.2 | 347-446 | TGGAGACCATAACCCGCAATTTAGAAGAGACCATCCAATCAGATTCAGCACCTTACGTCTTCCAGAGCAATATGAGATACAAACTGATAAGGCGAGTCAT |
| IL1B | XM_005068610.2 | 488-587 | AAGCTCTCCATCTCAACGGACAGAACATAAACCAACAAGTGGTGTTCTCCATGAGCTTTGTACAGGGAGAAACAAGCAACAACAAGATACCTGTGGCCCT |
| IL1R1 | XM_005088244.2 | 1520-1619 | AGCTCAGTGTAGAAGGAAGCCACACACAAGTTTCTTCTGCTCACTTGTCCACCCTGGAATGACTGTTGCATCATCTGTACCCGCCTGCACTTTGCTTATT |
| IL1R2 | XM_005075429.2 | 933-1032 | GATTGTCCCCTGCAAGGTGTTTCTGGGAACTGATACATCGTTCACCACCGTTGTGTGGTGGATGGCTAACAGCACTTTTATCTCAGTGGCCTACCCAAGA |
| IL1RAP | XM_005071593.2 | 1240-1339 | GGTACACAGTGGAACTCGCCTGCGGTTTTGGAGCCACAGTCTTTCTGGTGGTGGTTCTGATTGTGGTCTACCACGTTTACTGGCTGGAGATGGTCCTCTT |
| IL1RL1 | XM_013117334.1 | 489-588 | TCCAACAATTGGCCTCTATAATTGGACAGCACCTGTTCAGTGGTTTAAGAACTGCAAAGCTCTTCAAGGACCAAGGTACAGAGTACATAGATCATACTTG |
| IL1RL2 | XM_013117307.1 | 660-759 | CAGACTCACATACTTGGGGAGACAGTTCATGGTTAGAAACTACATTGCTGTGAGCATCAAGGAAGTTGTGTCTGGAGGAAGGATTCCTAATATCACGTAT |
| IL1RN | XM_005083743.2 | 515-614 | AGCCTTCAGAATCTGGGATATTAACCAGAAGACTTTCTACCTGAGGAACAACCAACTCATTGCCGGCTACTTACAAGGATCAAATACCAAATTAGAAGAA |
| IL2 | NM_001281629.1 | 440-539 | GACAGTAACTGTGGTGGAATTTCTGAACAGATGGATCACCTTCTGTCAAAGCAGTATCGCAACAATGACTCAATAATTACCTGCCTCCTACAACACATTG |
| IL20 | XM_005079861.2 | 139-238 | TCTGAGATTCGGGATAGTGTGCAAGCTGAAGATGAAAACATAGACATCAGAATCTTAAGGACGACGGAGTCTTTGCAAGGCATAAAACATTCGGATAGGT |
| IL21 | XM_005069697.1 | 126-225 | GAAAATTTATGTGAATGATTTGGACCCTGAACTTCTGCCAGCTCCACAAGATGTAAAGGAGCATTGTGAACGCTCAGCTTTTGCCTGTTTTCAGAAGGCC |
| IL21R | XM_005064448.2 | 331-430 | ATGTGTGGTACACGTGCCACATGCCCTTGTCTGGATTCATGGCCGATGATGTTTTCGTTGTCAACACGATGGACCACTCTGGCAACAACTCCCAGGAGTG |
| IL22 | XM_005081506.1 | 194-293 | GCCTTGCAGATAACAACACAGATGTCCGGCTCATTGGAGATGAACTGTTCCAAGGAGTCAATAGGCAGAATCGATGCTACCTGATGAAGCAGGTACTCAA |
| IL22RA2 | XM_005065729.1 | 508-607 | TACCGAGTTTTCATAACTGACAATTCACTAGGAAAGGAGCAAACGGCCTATGAAGGGACTCAGAGAACTGTTCAAATTGAAGGTCTGACAGCTCGTTCTG |
| IL23A | XM_005079727.1 | 260-359 | GCCAGTTCTGCTTGCAAAGGATCCGCCAAGGTCTGGTCTTTTATAAGCAGCTGTTGGACTCAGACATTTTTACAGGGGAGCCTGCTCTACTCCCTGATGG |
| IL23R | XM_005076112.1 | 486-585 | CATAGACACCAAGTATGTGGTGCACATGAGGAGTTTAGAAACAGAAGAAGAGCAACAATATCTTGCCTCGAGCCATGTGAATATCTCCACTGACTCATTA |
| IL24 | XM_013120372.1 | 190-289 | AGTGTCCAGCTGTTGAAGCCACAGGTTCTTCAGGATGTCTCGGATGCTGAGAGCTGCTACCTTGTCCACAGTCTGCTGAAGTTCTACCTGAACACTGTTT |
| IL25 | XM_013124877.1 | 474-573 | CAGAGGGGCTCCCACATGGGCCTCATGGGCAACTCAGTACCACTCTACCACAACCAGACGGTCTTCTACAGGCGGCCATGCCACGGACAGCAGGGCTGCC |
| IL27 | XM_005088471.1 | 175-274 | AGCTATGTCCACAGCTTTGCGGAATCGCGACTGCCAGGAGTGAGCCTGGACCTCCTGCCGCTGGGACACCACCTCCCCAATGTTTCGCTGACCTTCCAGG |
| IL28A | XM_013115342.1 | 52-151 | CCCCTTCAAGAGCTGCAGGCCTTCAAGAAGGCCAAGGATGCCATAGAAGAGTGGTTGCTCAGGAAGGGTGTCAGGCGCAGCTCCTGCCTCTTCTCCAGGG |
| IL2RA | XM_005071734.1 | 426-525 | GGGAACATGAAGATACCAAGAGAATCTACCATTTCGTGGTAGGACAGAGACTTCACTATGAGTGCATTCAAGGATACAAGGCCCTACAGAAAGGTCCTGC |
| IL2RB | XM_013111063.1 | 944-1043 | GCGGTCACTCGCAGGCCAGCTGCTTTACCAACCAAGGCTACTTCTTCTTCCATCTGCCCAACGCCTTAGAGATTGAGCCCTGCCAAGTGTACTTCACCTA |
| IL2RG | XM_005081186.2 | 770-869 | TGTGCTCATCCCGGTTGGCTCCATGGGACTGATTATTACCCTGATCTTTGTGTATTGCTGGCTAGAACGAGCAATGCCTCGAATTCCCACCATCAAGAAT |
| IL4 | XM_005067769.1 | 144-243 | TCATTCACATTTTGAACCAGGTCACAGAAAAAGGGACACCATGCACCGAGATGGTCGTACCAGACGCCCTTTCAGCAAGGAAGAACTCCACGGAGAAAGA |
| IL4R | XM_005063394.2 | 172-271 | ACCTGCTTCTCTGACTACATCCGCAATTCCACTTGTGAGTGGTACCTGGATGGCGCTGTCGACTGCAGATCTCAGCTCCACCTGTTATACTGGCTGGACT |
| IL5 | XM_005067909.1 | 121-220 | CTACTGACAAGCAACGAGCACCAGCTGTGCATCGGAGAAATCTTTCAGGGTCTAGACATACTGAAGAATCAAACCGTCCGTGGGGGTACCGTGGAGACGC |
| IL5RA | XM_005066186.1 | 556-655 | ACACTGAACAGAAATGTTGCATGCTGGTTTCCCAGGACATTTATCAACAGCAAAGGGTTTGATCAGCTTGCAGTACATGTTAATGGCTCAAGCAAGCATG |
| IL6 | XM_005087110.1 | 274-373 | CCGGTGATACAAATAAATGATGGATGCCTCCAAACTGGATACAATTGGGAAATTTGCCTATTGAAAATCACCTCTGGTCTTCTGGACTACCAGATCTACC |
| IL6R | XM_013120699.1 | 762-861 | GAGTCCTGGGACTCAAGTTACTACATGTTGAAGTTTGAGCTTCGATACAGACCTGTGTGGTCAAAGTCATTCACAGTGTGGCTGCTCCCGGTGGCCCAGC |
| IL6ST | XM_013125522.1 | 2254-2353 | CAAACAGAACTGTAGGCAACCTGAAGCCAGTCCAGATATTCCACATTTTGAAAGGTCAAACCAAGTGTCATCGGGCAGTGAGGAGGATTTTGTCAGACTG |
| IL7R | XM_005078037.2 | 244-343 | TCTGCCAGCGTTGAAAAGTCAGAATTGTCTTTAAGACAGTGGGTTGGGTGGGCTGGGTGCAAGCATGTAACTTTATCGAATACAAGAACAACAATCCTAG |
| IL9 | XM_005078463.1 | 229-328 | TCACAGCTGACCAACACCACACAGAACGCAGAAATCTCAGTTGTTTTCCAACGGGTGAAAAAGACAGTTGATAGCCTAAAGATCAGCAAGTGTCCGCTTT |
| ILF3 | XM_005078545.2 | 1986-2085 | AACGATTTGTTATGGAGGTTGAAGTGGATGGACAGAAGTTTCAAGGTGCTGGTTCCAACAAAAAGGTGGCAAAGGCTTATGCTGCTCTTGCTGCATTAGA |
| IRAK1 | XM_013125875.1 | 888-987 | CACCCAAATATCGTAGATTTTGCTGGCTACTGTGCAGAAAGTGGCTTCTATTGCCTTGTTTATGGCTTCCTGCCCAATGGCTCCTTGGAGGATCGACTCC |
| IRAK2 | XM_013110313.1 | 1413-1512 | GGACCTCAACTCACACAGACAACAAACTCCAGGAAACATTCTTCCTTTATGAGACTTCTGGCCTTCCACACCAAACGTCTGGTTTGTGACCCAAGGATTT |
| IRAK3 | XM_013121539.1 | 895-994 | AGCTTGTATTTTGCGGAAGACCCTCCCACATCTCTGAAGTCCTTCAGGTGTCCTTCTCCTCTGTTCTTGGACAACATCCCAAGTATCCCAGTAGAAGATG |
| IRAK4 | XM_013111016.1 | 1463-1562 | ATGCTGACCCTGCTTCCGTTGAAGCGATGTATGCTACAGCTAGTCAGTGTCTGCACGAAAAGAAAAATAAGAGGCCAGACATTACGAAGGTTCAACAGCT |
| IRF1 | NM_001281646.1 | 1090-1189 | ACAGCCTGCTGCCACCCTCTGTGAGGCTATCCTCCATTCAAGCCATTCCTTGTGCACCATAGTTTGGGTCCCTGACCCATTCTTGCTCTTCTGGGTGAGC |
| IRF2 | XM_013110855.1 | 439-538 | AGAACCAGTTGAGTCATCTTTGGGGCTTAGTAATGGAGTAAGTGACCTTTCTCCTGAGTATGCGGTCCTGACTTCAGCTATAAAAAATGAAGTGGATAGT |
| IRF3 | XM_005084741.2 | 810-909 | ACCCTTTTGACCCTCATAAAGTGTATGAGTTTGTGACTCCAGCAGCAAGAGACTTTGTACATCTGGACACCTCTCCTGATACCAATGGCAAAAGCAGTCT |
| IRF4 | XM_005066422.2 | 223-322 | GCACTATTTAAAGGAAAATTCCGGGAAGGCATCGACAAGCCCGATCCTCCTACTTGGAAGACAAGATTACGATGTGCTCTGAACAAGAGCAATGACTTTG |
| IRF5 | XM_013119090.1 | 1450-1549 | AGAGAGGCCGCAGGCCACGTTCTCCCTAAAATTCAATTTGAGCACTGTCTTGGGAGTGATTTCTCTAATGTTTGGCTCTGAAGAAATGTAGCCAGCAGCT |
| IRF7 | XM_005063345.2 | 565-664 | TCACACAGTCTTCCGTGTGCCCTGGAAGCATTTCGGTCGCAGGGATCTGGATGAAGCTGATGCACGTATCTTTAAGGCCTGGGCCGTAGCCAGAGGGAGG |
| IRF8 | XM_005073104.2 | 592-691 | CCACTGGTGACCGGGTATGCCGCTTATGACACACATCATTCAGCCTTCTCCCAGATGTTGATCAGCTTCTACTATGGGGGCAAGCCCGTGGGCCAGGCTA |
| IRF9 | XM_013124894.1 | 752-851 | GTGCCCTCAACAAAAGTCCTGAATTTGAAGAGGTTCCTGAGAGAGGCCGTATGGATGTTGCTGAACCCTACAAAGTATATCGGATACTGCCACCAGGAAC |
| IRGM | XM_013111756.1 | 552-651 | TCCTTTACTCTCAAGAAGTCAATGAGAGAATTGCTCAGGCAGTGAAAGAGGGGAACCTGTGGAATGTGATCTCTATGGTCAGAAATATAATAATGCAGCG |
| ISG15 | XM_013119951.1 | 171-270 | AGAGGCAAATTTTCCACAAGACTGGTGTGCCAGCTTTCCAGCAACGCCTGGTCCATGAAAGTGGCACGATGCTACAAGAGAACATTACCCTCATCAGCCA |
| ITGA2B | XM_005070111.2 | 2227-2326 | AGCAGGGCACCCGGATCGGAATCAGAATGTTGGTGAGTGTGGAGAACCTAGAAGAAGTGGGACAGTATGTGTCCTTCCAGCTTCAGATCAGGAGCAAGAA |
| ITGA4 | XM_013122725.1 | 2817-2916 | GCCATCCGTCTTGGAAATGGATGAGACTTCATCGCTCAAGTTTGAAATAAGAGCAACAGCTTTTCCAGAGCCGCATCCAAAAATTATTGAGCTAAACAAG |
| ITGA5 | XM_013111374.1 | 2286-2385 | GGTGGCCTTCGGTTCACTGTTCCTCATCTTCAAGACACTAAGGAAAACATCCAGTTTGACTTCCAGATCCTCAGCAAGAACCTGAACAACTCGCAAAGCA |
| ITGA6 | XM_013119539.1 | 2397-2496 | GGTGATTAACTTAGGCAAACCTCTTAAAAACCTCGGCACGGCGACCTTGAATATACAGTGGCCCAAGGAAATTAGCAACGGCAAATGGTTGCTTTACTTG |
| ITGAE | XM_013111466.1 | 1788-1887 | CAGAGATTGCTATTGTTCTGGATGGCTCAGGAAGCATTGAGCCGGAAGACTTCCAGAGGGCCAAAGATTTCATCTCCAACATGATGAGGAACTTCTATGA |
| ITGAL | XM_005064364.2 | 3055-3154 | CCTCCTGTCTTTTGCCACAGTGAGGACATGAAGAGGCCATCTAATGAAACTGAGCCTTGTCTACCTGGAATTCTGTTCCGCTGTCCAATTGTCTTCAGGC |
| ITGAM | XM_005064322.2 | 1187-1286 | GGGGAGCCTTTCTGTACACATCAAAGGATAAAGTCACCTTCATCAATACAACCAGAGTGGATTCAGATATGAACGATGCTTACTTGGGCTATGCTTCTGC |
| ITGAX | XM_013111423.1 | 835-934 | GCTGCGGGCATCATTCGTTATGCAATTGGGGTAGGACAGGCCTTTTCCAATGCACATTCTTTGCAAGAATTAGTGAACATCGCATCGACGCCTTCCCAAG |
| ITGB1 | XM_013123083.1 | 1900-1999 | AGACTCCAGACTGTCCTACTGGTCCCGATATCATTCCAATTGTAGCAGGTGTGGTTGCTGGGATTGTTCTCATCGGCCTTGCCTTGCTGCTTATTTGGAA |
| ITGB2 | XM_013113856.1 | 1524-1623 | CGCTGCCAAGGATCCAAAGGCCCACTGTTTCCTTCCACCTTATGTCAAGGATGCCAGGTTTGCCACGAACTCATCTTCTGACCTGCACACTCTTGCCACA |
| ITK | XM_005071895.2 | 784-883 | TTCCTGGTCCGAGATTCCCGGATACCCGGCACATACACAGTCTCTCTGTTCACCAAGGCCATCATAAGTGAGAACCCCTCTATAAAACATTATCATATCA |
| ITLN1 | XM_005087783.2 | 396-495 | AGTGTTCAATAATGAGAGAGCAGCCAATGCCTTGTGTGCTGGTATGAAGGTCACTGGATGCAATACTGAAATTCACTGCATTGGTGGCGGAGGATTCTTC |
| JAK1 | XM_005086635.2 | 3531-3630 | CTGTGACTCGGATTTTAGTCCCATGGCCTTGTTCCTGAAAATGATAGGCCCAACTCATGGCCAGATGACAGTAACACGGCTTGTGAATACTCTGAAAGAA |
| JAK2 | XM_005063713.2 | 2659-2758 | GTGCTTTTGAAGATAGAGACCCTACACAGTTTGAAGAGAGACACTTGAAGTTTCTACAGCAACTTGGCAAGGGTAATTTTGGGAGTGTGGAGATGTGCCG |
| JAK3 | XM_013125268.1 | 380-479 | AGGCTCCGCTTCTATTTTCCTGACTGGTTTGGGCTGGAGACTTGCCACCGCTTCGGGCTGCGCAAAGATTTGACCAGTGCCATTCTCGACTCGCATGTTT |
| JUN | XM_013116518.1 | 1383-1482 | AACGGGCTGTGCCCGCGCGACCAGAACGATGAACTTTTCTTTAACATTGACCAAGAACTGCATGGACCTAACATTCGATCTCATTCAGTATTAAAGGGGG |
| KCNE1 | XM_013116135.1 | 1215-1314 | CAAATCTCTGGACATTAGCGGGTCACATGACTAAGTTCTGGCCAAAAGATATGGACATGATGAGCCCAGTTCGGTCTTTACCAACGATGGCGTTTCATTG |
| KCNJ2 | XM_005069969.2 | 637-736 | TCGTTGGCGATGGATGCTGGTTATCTTCTGCCTGGCATTTGTGCTCTCCTGGCTGTTCTTTGGCTGTGTGTTTTGGCTGATAGCTCTGCTCCACGGGGAT |
| KDR | XM_005080774.2 | 697-796 | GACATTGTGTACATCACCGAGAATAAGAACAAAACTGTGGTGATTCCGTGCCGAGGGTCAATTTCAAGCCTCAACGTGTCACTCTGTGCAAGATATCCAG |
| KEAP1 | XM_005078552.2 | 1624-1723 | GGGGGCTTCGATGGGACTAACCGACTTAACTCAGCAGAGTGTTACTATCCGGAGAGGAATGAGTGGCGGATGATTACACCAATGAATACCATCCGAAGCG |
| KIT | XM_013121055.1 | 3422-3521 | GAAAGTGAAACCCAAGCCCTTTGTGTGGAAAAGTAGACCATTATTAGAGCAGAGGACATGTGAAAACATCGGGGCTTAAGAAATCTAGTAGTTCATGCTG |
| KLRB1 | XM_005084084.2 | 491-590 | TGTGGAAGTGGATCAATGGCTCTACTTTAAACCCTGACCTATTACAGGTCACTGGCAAGGCCAAAGAAGACAGCTGTGCTGTTATCTCGAAGACAGAAGT |
| KLRC1 | XM_005087144.1 | 155-254 | ATCCCACAGTCTGCAGGGACTGCTGCTTCAAAGGTTTTCCATTTCCTCCAGAAAAACTCATCACTGGGATTCTGGGCATTATCGGCTTGGCCTTACTGGT |
| KLRG1 | XM_005066036.2 | 420-519 | CTGTGCGTACAAAGGCTCACATCTCCTTACATTTCTGGACGACAAGGAAGTGATTCCCTTCCAAGAGTACTTGGACAACGACTTTTACTGGATCGGCTTG |
| KLRG2 | XM_005085787.2 | 873-972 | CAGAGGCGGAGCCAGGTGCCAGTCATGCCCTCAAGGCTGGTTGTGGTCTCAGGAACACTGTTATTACCTCTCTGAAGAAGCCCAAGACTGGGAGGGCAGC |
| KLRK1 | XM_005087145.2 | 118-217 | TTACCAGCCAATCCAGAGAAAACGGTATTATAAGAAGAAACTCCCCTATAGAAGAACTCAAAATATCTCCATCGTTCGTTACCCGAGTCATTGCTGCAGC |
| KNG1 | XM_005071611.2 | 1360-1459 | GTCGAACCGACCGTCAACTGCCGAGCATTAGAAATGATTACAATGATGAGAAGGCCTCCAGGCTTTTCACCTTTCCGGTCAGTCCAAGTACAAGAAACAA |
| LAG3 | XM_013110290.1 | 1418-1517 | CACTGTTCCTTTTGGTGACTGGGGTCTTTGGATTTCACCTATGGAGAAGACAGTGGCGGTGGAGAAGGTTTTCCGCCTTGGAGAATAGGATTCACCCACC |
| LAIR1 | XM_013123705.1 | 1076-1175 | AGTATCAGTCCTTGGAAAGACCATGTTGCAGTAAGAGCCGCAGTTACAACCACCTAGCTATTTCCAGAGACAGAGTTACAGCTTGTTAGCTACAGCCTGT |
| LAMP3 | XM_005077577.2 | 499-598 | TTTTTCACAGCATTACACAAAACCACAACCAATCAGAAGCCAACTCCACCCACCTATGTTCCAGGAGCATCAGTACCTACACACAAGGTCAGCTTTGAAT |
| LAT | XM_005064443.2 | 129-228 | GAGTTTGTCCCCACGGAGTATCCTCATCAAGCCGTCTCAAACAGCCTTCTCCAGAGCAACTGCCAGTTCCTATCCTCCTGTTACTTCCTTTCCGCCCCTG |
| LCK | XM_005088216.2 | 989-1088 | CTCCCTCAGGCATCAAGTTGAACATCAACAAACTGTTGGACATGGCAGCACAGATCGCAGAGGGTATGGCATTCATTGAAGAGCAGAATTACATCCATCG |
| LCN2 | XM_005087677.2 | 264-363 | GGCAACACAGTCCAGAAAGAAGAACAAGGCCGTTTTATGATGTACAGCACCATCTACCAGCTGCAAAACGACACCAACTACAATGTCACCTCCATCCTGC |
| LCP2 | XM_005071991.2 | 309-408 | TGAGAAGGCGGTGAAGAAATACCGTCTGCCAAGTGTCTCTTTTTTACAGAACCTGACAGAAAATGACATCCAGAAGTTTCCTAAGCTGAGGATGCCAATT |
| LEF1 | XM_005082802.2 | 1016-1115 | CCCGGGAAGAGCAGGCCAAATATTATGAATTAGCACGGAAAGAAAGACAGCTACATATGCAGCTTTATCCAGGCTGGTCAGCAAGAGACAATTATGGCAA |
| LEPR | XM_005086642.2 | 3575-3674 | TGAATACACTGCCAAGCCATCACGGGGAGGTCTCTTTATTTCTAGCTAATAATAAGCCCAGGAAGTAGGATTTCTCTAAACATCTAGCCTCTGTGGAAAG |
| LGALS3 | XM_005085734.2 | 631-730 | CAGCTTTCCCATTCGAAAGTGGCAGACCGTTCAAAATACAAGTCCTGGTTGAAGCTGACCATTTCAAGGTTGCAGTCAACGATGCTCACTTGCTGCAGTA |
| LIF | XM_005068926.2 | 249-348 | GCCCAACATGACAGATTTCCCACCTTTCCATCCCAACGGGACAGAGAAGACCAAGTTGGTGGAGCTGTATCGGATAGTCGCATACCTGAGTGCTTCCCTG |
| LILRA2 | XM_013123687.1 | 313-412 | AAGCTCTCCTGGATTATGGACTCACAATATAACCAATCTTCTGGGCAGTTTGAAGCTCTGTTCTTTCAGGGCTCTTTGACTCCCAACCAGCGTTGGACAT |
| LILRA5 | XM_013123703.1 | 100-199 | TGGTTGCCAGAGGGAAGCAGGTAACCATCTCATGTGAGGTGACCACAGGAGCCCGGGAATTCCGTCTTTACAAAGAGGGGGGTCCACATCCCTGGCGCAC |
| LILRA6 | XM_005084183.2 | 2031-2130 | TAAGAAGCACACAGAGATTCCAGCGAATCCTAACAACAAGGCTGAATTCTCAATCAAAAAAATAAGCCGTCATGAAGCAGGGCAATACACCTGTCGATAC |
| LILRB3 | XM_013123690.1 | 531-630 | AACCTGGGGTACACAGACCCCAAAGGAGTCTGGAAACAAGTCCACGTTCTCCATTCCTTCTGTTGCAGACTACAGTGGGGGGAATTATCGCTGTTACTGT |
| LILRB4 | XM_013123676.1 | 228-327 | AACAAGGGAAAAGGCCAAGTTATACTGTCAACACATGACAATCCAACATGAAGAGATATATACATGTCACTATAAGAGCACTGCAGGTTTTTCAGAGCAC |
| LIMK1 | XM_013120758.1 | 605-704 | GGAGCACTCTAAGCTGTACTGTGGGCACTGCTACTTCCAGACTGTGGTAACTCCAGTTATTGAACAGATCCTGCCCGACTCACCCGGCTCCCACCTCCCC |
| LITAF | XM_005084576.2 | 611-710 | ACCCAGAAGAGGCACCTACCTTGATTTCATCTCTTCATGGGCTCCATCTTCGTGTCTTTTGGGGGAGGGGGTGTCAAAAAAGTAACAAGCCTCCAAACCC |
| LOC26010 | XM_005080895.2 | 802-901 | AGCGCTGCACTGTTTCTCTGACTAGATACCGTGTCATGATCAAAGAGGAAGTGGACAGTTCTGTGAAGAAGATCAAAGCAGCTTTTGCTGAGTTACACAA |
| LRIF1 | XM_005076592.2 | 1875-1974 | GTCTCACTAAAGATTTGAGAGTGGATCTTACTCGCATCCCTGACTGTCTGGGCTCTAGGAAAGGTTCTGATTCCTTTAGCAGTTTGATGAAGAGTAACTC |
| LTA | XM_005086798.1 | 355-454 | CCCATCTACCTGGCACACGAAGTCCAGCTGCTTTCCTCCCAGTATCCCTTCCATGTGCCTCTCCTCAGTGCGCAGAAGTCCGTGTATCCGGGGCTACAAG |
| LTB | XM_005086801.2 | 140-239 | TGCCCCAGGATCAGGGACGTCGGGTTGAGAAGATCACTGGCTCGGGAGCACAGGCTCAAAAGAGACTGGATGACCACAAGCCATCTTGCCTCCTGCCCTC |
| LTB4R | XM_005085619.2 | 441-540 | TGTGGGTGTTGGCAAGCATCTGGTTGGTGTCTTTTCTGCTGGCCACACCGGTCATTGCGTACCGCACAGTAACGCAGAACAACAGGACTCTGACTCTGGT |
| LTB4R2 | XM_005085618.2 | 439-538 | CTGGTCGCCCTGGTGCTCGCCATCCCGGCCGCCGTCTACCGCCACCTCTGGGGCGATCGCGTGTGTCAACTGTGCCACCCATCGGCGGTGCACGCCGCTG |
| LTBR | XM_013123606.1 | 653-752 | GCCCGGCACAGAAGCCGAGGTTACAGACGAGGTTATGGATACTGACATCAGCTGTGTCCCCTGCAAGCAAGGATACTTCCAGAATACTTCCTCCCCTACA |
| LTF | XM_013116962.1 | 1537-1636 | TGCCTGTTCCGGTCTGAAACCAAAAATCTTCTGTTCAATGACAACACTGAGTGTCTGGCTAGGCTCCATGGCAAAACAACATATGAGAAGTACCTGGGAA |
| LTK | XM_005064508.2 | 76-175 | GCTGGGAGCTGCCGGAACTATTCTTTGCTCCAACTCGGAGTTCCAGGCACCTTTTCTAATATCCTCACCCTTGCCGGTGCTAGTCGCCAACTCACAGGAG |
| LY6E | XM_005085960.2 | 1347-1446 | GGGGGGAACTACCCAAATGAATATATACCTGTGTGCGCAGTGTGTCTGTTTGTGAATGAAGCCATTCGGGATCTGGGATGGGCAAAGGGGACCTGGAAGA |
| LY96 | XM_013110915.1 | 215-314 | TACCATTTTCCTTCAGGGGAATACTATTTCCAAAGGGCCAATACAGATGTGTCGCAGAAGCCATTGTAGGGGATAATGAAGAAAAGCTGTTTTGTTTGAA |
| LYN | XM_005087588.2 | 1120-1219 | ATCATGAAAATGTGTTGGAAAGAAAAAGCAGAGGAGAGGCCGACGTTTGACTACTTACAGAGCGTCTTGGATGATTTCTACACTGCCACGGAAGGGCAGT |
| MAF | XM_013115615.1 | 2008-2107 | TGCCACAGTCAAGCCTGTATCAAGCTCCTGGGTATGACTCCTGAGTTTTGGCCTTAGGATGCCAAATCTGGAAGTTTAGCCTGCCATTAAAAAGCAAACA |
| MAFF | XM_005066981.2 | 302-401 | CCGTGGTCTCCAGCCCAAAGGGCACCTTCTGCAAAAATGTCTGTGGATCCCTTATCCAGCAAAGCCCTGAAGGTCAAGCGTGAGCTGAGCGAGAACACGC |
| MAFG | XM_005069800.2 | 3487-3586 | GACTGATGAGGAGCTGGTGACCATGTCCGTGCGGGAATTGAACCAGCACCTGCGAGGCCTGTCCAAGGAAGAGATCATCCAGCTGAAGCAGCGCCGCCGC |
| MAFK | XM_005079985.2 | 163-262 | GCTACGATTTCCTGGTGGTTCCGTCCTGGTGACAGGGCCCGGGTTATGACGACTAATCCCAAGCCGAACAAGGCATTGAAGGTTAAGAAGGAGGCGGGCG |
| MALT1 | XM_005074657.2 | 884-983 | TCAGAATCCAAGTTGCAAATCTGTGTTGAACCAAGGTCCCAAAAGCTGATGCCAGGCAGCACATTGGTTTTACAGTGTGTCGCTATTGGAAGCCCTATGC |
| MAP2K1 | XM_005075560.2 | 785-884 | ACATGGATGGTGGGTCCTTGGATCAAGTTCTGAAGAAAGCTGGAAGAATTCCTGAGCAAATTTTAGGAAAAGTTAGCATTGCTGTGATAAAAGGCCTGAC |
| MAP2K4 | XM_013111487.1 | 943-1042 | AGTGCGTCACGACAAGGGTATGATGTCCGCTCTGATGTCTGGAGTTTGGGGATCACATTGTACGAGTTGGCCACAGGCCGATTTCCTTATCCAAAGTGGA |
| MAP2K6 | XM_005069971.2 | 965-1064 | GTACAGTGTGAAGTCTGACATTTGGAGTCTGGGCATCACTATGATTGAGCTGGCCATCCTTCGGTTTCCCTACGATTCTTGGGGAACTCCCTTCCAGCAG |
| MAP3K1 | XM_005065477.1 | 2584-2683 | CCAGAGAACAGTTCCCTTGAGCACACAGCTCATTTAGAGAAAACTGGAAAAGGATTATGTGCTATGAGACTGAGTGCCAGTTCAGAGGACATTTCTGACA |
| MAP3K5 | XM_005065835.2 | 1520-1619 | GACATGTTTTTGGATTCCAATTTCACTGACACTGACAGCAGGGACCATGGAGCATCTTGGTTCAAAAAGGCGTTTGAGTCTGAGCCAACACTACAGTCAG |
| MAP3K7 | XM_013121065.1 | 941-1040 | TGATGAGATCGGTGGCCCAGCTTTCCGAATCATGTGGGCTGTTCATAATGGTACTCGACCACCACTGATCAAAAACTTACCTAAGCCTATTGAGAGTCTG |
| MAP3K9 | XM_013115430.1 | 2913-3012 | GTGTATCTTCTGCTCTTCCTGTGACTTGGTGTCTCCTGTGGTATGCAATGAATGTCTTGTGTCCTAAGTCTTCTTGTGCTAGCTACTGGGGACAGGTTGG |
| MAP4K1 | XM_013125343.1 | 2154-2253 | GGCATTTTCATTCTCAACCGGAACGACCAGGAGGCCACACTGGAGATGCTCTTCCCTGGCCGGACAACCTGGGTGTATTGCATCAACAACCTCCTCATGT |
| MAP4K2 | XM_005063941.2 | 941-1040 | TGGGAGCCCTCTCCCCTGAAGACTGTGAACTGGAGACCCAGGATATGTTTCCAGATACCATCCATTCCCGGGGCCATCATGGTCCTGCTGAGAGGACCCC |
| MAP4K4 | XM_005075368.2 | 2865-2964 | TACTACAGATTTCTCCATCTAGTGGAACGACAGTTACTTCCGTGGTGGGATTTTCCTGTGACGGAATGAGACCAGAAGCCATAAGGCAAGATCCTACTCG |
| MAPK1 | XM_005077568.2 | 982-1081 | AAAAGCTAGAAACTACTTGCTTTCTCTCCCGCACAAAAATAAGGTGCCGTGGAATAGGCTGTTCCCAAATGCTGACTCTAAAGCTCTGGACTTACTGGAT |
| MAPK11 | XM_013111098.1 | 359-458 | TTCACGCCGGCCACATCCATCGAGGATTTCAGCGAAGTGTACCTGGTGACCACCCTGATGGGCGCTGACCTGAATAACATCGTCAAGTGTCAGGCACTGA |
| MAPK14 | XM_005084901.2 | 401-500 | CATGGGGGCAGACCTGAACAACATCGTGAAGTGTCAGAAGCTTACCGATGACCACGTTCAGTTCCTTATCTACCAGATCCTCCGAGGGCTGAAGTATATA |
| MAPK3 | XM_005064413.2 | 796-895 | ATCAACATGAAGGCTCGAAACTACCTACAGTCTCTGCCCTCTAAAACTAAGGTGGCATGGGCCAAGCTTTTTCCCAAATCTGACTGCAAAGCCCTCGACC |
| MAPK8 | XM_005077642.2 | 595-694 | TTCTCTATCAAATGCTGTGTGGAATCAAGCACCTTCACTCTGCTGGAATTATTCACCGGGACTTGAAGCCTAGTAATATAGTAGTCAAATCAGACTGCAC |
| MAPKAPK2 | XM_005079859.2 | 776-875 | CCCTTCTATTCCAATCATGGCCTTGCCATCTCACCGGGTATGAAGACTCGTATCCGAATGGGCCAGTATGAATTTCCTAACCCAGAATGGTCAGAAGTAT |
| MAPKAPK5 | XM_013120919.1 | 521-620 | AGAGGAAGTTACTTGGCACCAAGCCAAAGGATGGTATTTATATACACGACCATGAGAATGGAGCTGAGGATTCAAATGTTGCCTTGGAAAAGCTTCGAGA |
| MARCO | NM_001281606.1 | 1233-1332 | CTGAAATTTTCTATAACAACGCCTGGGGGACAATTTGTGATGACAACTGGGATAATAACGATGCCACTGTCTTCTGCCGCATGCTCGGTTACTCCAGTGG |
| MASP1 | XM_005071606.2 | 1276-1375 | CATCAGCTGTGACACGGGCTACAAAGTTCTAAAGGATAGTGAGATAATGGACACGTTCCAGATCGAATGTCTGAAGGACGGTACATGGAGTAACAAGATT |
| MASP2 | XM_005079315.2 | 1436-1535 | CTCTTTTACACGACAACTGGGTTCTAACAGCTGCTCATGCTGTGTATGGGATAGCAGAGGCAGTGTCTTCTCTGGACATTCGCATGGGCATCCTCAAAAG |
| MAVS | XM_005068650.2 | 965-1064 | CAAAGTGTCCTCCAAGTTGCCCATCAGTACAAAGTCCACTGCTGCAATGCCTTCTGCTGTGCTCACCAATATAGCACCGTCAAAGTTACCCATCAACTCA |
| MAX | XM_005072751.2 | 425-524 | ACAGAGTATATCCAGTATATGCGAAGGAAAAACCACACGCACCAGCAAGACATTGATGACCTCAAGCGGCAGAATGCTCTTCTGGAGCAACAAGTCCGTG |
| MBL2 | XM_013114293.1 | 743-842 | CGGAATGCTGAGGAAAACAAGGCCGTTCAGAAGGTGGCTAGAGACATTGCCTACCTGGGCCTAACAGATGAGAGGACTGAAAACCGATTTTTGGATTTGA |
| MBP | XM_013123837.1 | 1169-1268 | GCTCAGCCTTCCCGAATCCTGCCCTCGGCTTCCTAATATAACCGCCTTAAACTTTATTTCTACCTGCACCAATGAGCTAGTTAGAGCAGACCCGCTGCTA |
| MCL1 | XM_013123782.1 | 556-655 | GATGGCGTAACAAACTGGGGCAGGATTGTGACTCTTATTTCTTTTGGTGCCTATGTGGCCAAACACTTGAAGAGCATAAACCAGGAAAGCTGCATCGAAC |
| MEF2A | XM_013123467.1 | 616-715 | CAAATCACACGCATAATGGATGAGAGGAACCGGCAGGTCACTTTTACAAAGAGAAAGTTTGGTTTGATGAAGAAAGCCTATGAACTCAGTGTGCTGTGTG |
| MEF2BNB | XM_013125322.1 | 829-928 | GATTTAGTGGGAAGTTGAGCTTGGCTGGGTTTCTCCAGGTGAGGTCCAGTGAAACGAAAGCGCCACTGGCCTCGGAGTTTGTGTTGGTGTAACGCAATAA |
| MEF2C | XM_005065693.2 | 1484-1583 | ATCAAAGGATAAATAACTCCCAGTCGGCTCAGTCATTGGCTACTCCAGTGGTTTCCGTAGCAACTCCTACTTTACCAGGACAAGGAATGGGAGGGTATCC |
| MEF2D | XM_013120610.1 | 3996-4095 | CCTCCCAGTTGGAGGGTAGAGTTAGACAAGGCTCAGTGTTGTAGTGAGATGGTCCTTGCCCACCTGTGACCCAGCCTTTTGTAGTATTTGACACTTGATG |
| MHC_IIA | XM_005086856.2 | 157-256 | CTACCAGTCTCAACAACCCAATGGCCAGTTCACGTTTGAGTTTGATGGCGATGAGTTGTTCTATGTGGACTCGGATAAGAAGGAGACGATCTGGAGGATT |
| MIF | XM_005070746.2 | 392-491 | ACATCAGCCCGGACCGGATCTACATCAACTATTACGACATGAGCGCGGCCAACGTGGGCTGGAACGGGTCCACCTTCGCTTGAGTGCTGGCCTCACTTAC |
| MKK7 | XM_005085285.2 | 1147-1246 | GGCTTCTCAGGGGACTTCCAGTCATTCGTGAAAGACTGCCTTACTAAAGATCACAGGAAGAGACCAAAGTATAATAAGCTACTTGAACACAGCTTCATCA |
| MKNK1 | XM_005072077.2 | 2210-2309 | CTGGCAAAGAATATGCTGTCAAAATCATTGAAAAGCAAGCAGGGCACAGTCGGAGTCGAGTATTCCGTGAAGTGGAGACACTATATCAGTGTCAAGGAAA |
| MME | XM_005077929.2 | 793-892 | GAATTCTACCCACCACATAATTCATTTTGACCAGCCTCGACTTGGCCTCCCTTCCAGAGACTACTATGAGTGTACTGGAATATATAAAGAGGCTTGCACA |
| MMP2 | XM_005079148.2 | 2145-2244 | GCAGGGCGGTGGTCACAGCTACTTCTTCAAAGGTGCCTACTACCTGAAGCTGGAGAACCAAAGTCTGAAGAGTGTGAAGTTTGGAAGCATCAAATCAGAC |
| MMP8 | XM_013118534.1 | 134-233 | TTGGCCATTCCTTAGGACTCTCCCACTCCTCTGATCCCGGTGCCTTGATGTATCCCAACTACGCTTACAGTGAACCCAGCACCTACTCACTACCTCAAGA |
| MMP9 | XM_005084984.2 | 589-688 | CCGGGCATTCAGGGAGACGCCCACTTCGACGATGAAGAGTTGTGGTCTCTGGGCAAAGGCGTCGTGGTTCCCACCTACTTTGGAAACGCAAATGGTGCCC |
| MPPED1 | XM_005067047.2 | 761-860 | GAAGAAGTTCAATGAATGGCTGGGTAGCCTGCCCTATGAATACAAGATCGTGATTGCGGGCAACCACGAGCTGACCTTCGACCAGGAGTTCATGGCTGAC |
| MR1 | XM_013114279.1 | 310-409 | AGTCACCTACAGAGGCACTACAGCCACTCAGGCATGATTCTTCACACTTATCAGAAAACGATGGGCTGCGAGTTGTTGAAAAATGGTAATGCCACAGGTT |
| MRC1 | XM_005081296.2 | 2048-2147 | GAAGCTACCATGAACTGTTTTGGTTAGGATTGACCTACGGAAGTCCTTCAGAGGGCTTCACTTGGAGCGATGGTTCTCCTGTTTCCTATGAAAATTGGGC |
| MRFAP1 | XM_005068842.2 | 212-311 | AGGTGGAGGTGCTGGAGCCCGAGGAGGACTTCGAGCAGTTTCTGCTGCCCGTCATCCACGAGATGCGCGAGGACATCGCGTCGCTGACGCGCGAGCGCGG |
| MS4A1 | XM_005063821.2 | 654-753 | TGATTTCTGCCTTCTTCCAGAAACTTGTGACAGCTGGCATCGTGGAAAACGACTGGAAAAGAATGTGCTCCAGACCCAAATCTAATGTGGTTCTGCTGTC |
| MS4A2 | XM_013110833.1 | 406-505 | GTCAGCAGCATCGCTGCAGGAACAGGGATCATCGTACTGATCCTCAACCTGAGCCACAATTCCACCTACCTGAAATACTGTAAGGATCTGAACGAGGACG |
| MSR1 | XM_005066635.2 | 585-684 | AGATCTCCAAGTCCTTGACGAGTCTCAATACTACACTGCTTGATGTCCAGTTCCATACAGAAACACTGAACAGCAAAGTCAGTGAGTCTACAGTGAAGCA |
| mTOR | XM_005079329.2 | 7181-7280 | GGGACTGCTTCGAGGTTGCTATGACCAGAGAGAAATTTCCAGAAAAGATTCCATTTAGACTAACAAGAATGTTGACCAATGCTATGGAGGTTACAGGTCT |
| MUC1 | NM_001281655.1 | 1523-1622 | CCAGCTCCAACTACTATCAAGAACTGAAGAGGAACGTCTCTGGATTGTTTCTGCAGGTTTTTAGCCGAGCTTTCCTGGGGATCTCTACCATCGAGTTCAG |
| MX1 | XM_013116138.1 | 1887-1986 | CAGGTCCCTCTGATCATCCAGTACTTTATCCTGCAAAGATTTGGGGAAGAGATGGAGAAAGCCATGCTTCAGCTCTTACAGGACAAGGTTAACTGTAACT |
| MX2 | NM_001281635.1 | 1667-1766 | GAAGAGGAGAACAACACAAAGTCAAGCTCCTTCAGTTCCTCTAAGTCTGAAGCTTTGCAGAACTCATCCATGGCTGAGATCTTTCAGCACCTGAATGCCT |
| MYC | XM_005078685.2 | 1566-1665 | ATCCTCAAAAAAGCCACCGCCTACATCCTGTCCATCCAAGCCGAAGAACACAAGCTCATCTCAGAAAAGGACTTACTGAGGAAGCGGAGAGAACAGTTGA |
| MYD88 | XM_005082348.2 | 877-976 | CCTGTGTCTGGTCCATTGCCAGTGAACTCATTGAGAAGAGGTGTCGCCGGATGGTGGTGGTTGTTTCTGACGATTACCTACAGAGCAAGGAATGTGACTT |
| MYL2 | XM_005080644.2 | 321-420 | TCTCAACGCATTCAAGGTGTTTGACCCTGAAGGCAAAGGGTCGCTGAAGGCTGACTATGTCCGGGAGATGCTTACCACCCAAGCAGAGAGATTTTCCAAA |
| NCAM1 | XM_013112926.1 | 1184-1283 | AACGCCGAGTATGAAGTATCTGTGGTAGCTGAGAATCAGCAAGGAAAATCCAAGGCAGCTCATTTTGTGTTCAAGACCTTAGCCCAGCCCACAGCCATCC |
| NCF4 | XM_005066953.2 | 713-812 | GAGGCCCTGTTTGACTTCACTGGGAACAGCAAACTGGAGCTCAGTTTCAAGGCTGGAGACGTGATCTTCCTTCTCAGCAGGGTCAACAAAGACTGGCTGG |
| NCR1 | XM_005088335.2 | 856-955 | ACCAACAGATCAGCAAGTTGGGAATGCAGAAGAAGACGGAGATCGCAATGTTCTCTCGAAGAGGAACAACGGGATGCAATTTCTATGGGGGAACTGAAGG |
| NEFL | XM_013117217.1 | 350-449 | TCCGTGCGCCGCAGCTACTCGTCCAGCTCCGGCTCGTTGATTCCCAGCTTGGAGAACCTCTATCTGAGCCAGGTAGCCGCCATCAGCAACGACCTCAAGT |
| NFATC1 | XM_013123448.1 | 3250-3349 | TGGCCGTCCTACACAATTTGCCTGCCTTATCCAGTGCATTTTAGAGGTCTTCCAATTGTCATCTCAGCTCTTTCGTAATGAGATCCCTCCTCCGTGTGAC |
| NFATC2 | XM_005074439.2 | 1877-1976 | TGGAAGCCACAGTGGATAAAGACAAGAGCCAGCCTAACATGCTTTTTGTTGAGATTCCTGAGTATCGGAACAAGCACATCCGCGTACCCGTGAAAGTGAA |
| NFATC3 | XM_005076354.2 | 414-513 | TCACTTGGCTTACCACATCATGGACTGCTGCAATCTCACTCTTCTGTTTTGTCACCATCATTTCAGCTCCAAGGTCACAAACATTATGAAGAAACTGGTG |
| NFATC4 | XM_013124875.1 | 2038-2137 | AAGCTGCGCCCGGAGGTCACCCCGTGGTCAAGCTCCTAGGCTACAGTGAGAAGCCACTGACGCTACAGATGTTCATTGGCACCGCAGATGAAAGGAGCCT |
| NFE2L2 | XM_005065132.2 | 632-731 | TTGGCAGAGACATTCCCATTTGTAGATGACCATGAGGTTTCTTCACCTGCATTTCAATCACTCGCCCTGGATATTTCCAGCCATGTTGAAAGCCCAGTCT |
| NFIL3 | XM_005066298.2 | 587-686 | GAAGCGCCGGCTCAATGACCTGGTTTTGGAGAACAAGCTGATAGCTCTGGGAGAGGAAAACGCCACTTTAAAAGCCGAGCTGCTCTCTCTCAAGTTAAAG |
| NFKB1 | XM_005081973.2 | 1023-1122 | AGGATGTCAACATTACAAAACCAGCGTCCGTGTTTGTTCAGCTTCGGAGGAAATCGGATTTGGAAACTAGCGAACCGAAACCCTTCCTCTACTACCCTGA |
| NFKB2 | XM_005063503.2 | 896-995 | CAGAAAGATGACATTGAGGTTCGGTTCTATGAGGATGACGAGAACGGATGGCAAGCCTTTGGGGACTTCTCTCCCACAGACGTTCATAAACAGTATGCCA |
| NFKBIA | XM_005080507.2 | 926-1025 | AGGAGAGTTACGACACAGAGTCAGAGTTTACGGAGGATGAGCTGCCCTATGATGACTGTGTGTTTGGAGGCCAACGTCTGACATTATGAGTGGAAAATGT |
| NFKBIZ | XM_005074792.2 | 1012-1111 | TCAGTTCTTACTCCAGAACATCTCAGTCTCCCAAGTACGAATCAAACCTCTTTGATAGTCAAGACCCACAGTTCTGCACGAGCCAGAGTTTTGTGTCTCT |
| NLRC4 | XM_013115956.1 | 1585-1684 | CACAGCTCAGAGGCTGACACCCAAGTATAAATTCTTTCATAAATCCTTCCAGGAGTACACAGCGGGACGAAGACTTAGCAGTTTGTTGATTTCCAGAGAA |
| NLRC5 | XM_013119507.1 | 1118-1217 | CTTTTTGAGTTCCGCCGGCTCAACATGGTCACACATATGCTGACGCTGCCCCAGCTCCTTTTTGATCTGTATCTGAGCCCCGAGTCAGACCCAGATGCTG |
| NLRP1 | XM_013111473.1 | 3630-3729 | TCTGATGTCAGACTCAGGAACTACATGCTACATGGATCTCACTCTCATCACAAGCGCCTTACAACTACTGAGATCAGAACTTAAAAAATTGAGACGAACT |
| NLRP3 | XM_013111444.1 | 2065-2164 | TCCCAAAATTGAGATCAACCTCTCTACCAGAATGGACCATGTGGTTTCTTCCTTTTGTATCAGGAACTGTCATCGGGTGAAAACACTTTCCCTGGGGTTT |
| NOD1 | XM_005076802.2 | 1924-2023 | CATCTCACACTCCAGTCCTTCTTTACCGCCTTCTTCCTGGTGGCAGATGACAAAGTGGGCACCAGAGAGTTGCTGAGGTTCTTTCGAGAGTGGACGTCTC |
| NOD2 | XM_005079168.2 | 2813-2912 | ATGTCTGTGACGAAGGGGTGTATTTTCTCGCAGAAGGACTGAAGAGAAATGCAAGTTTGAAAATCTTGAAGCTGTCCAACAATGGCATTAGCTACCGGGG |
| NOS2 | NM_001281644.1 | 3481-3580 | TGGCCACCAAGCTGAACTTGAACGAGGAGCAGGTTGAGGACTATTTCTTCCAGCTCAAGAGCCAGAAACGTTATCACGAGGATATCTTCAGCGCAGTCTT |
| NOTCH1 | XM_005083646.2 | 1588-1687 | TGAGATTGATGTCAATGAGTGCATCTCCAACCCATGTCAGAATGATGCCACCTGCCTGGACCAAATTGGGGAGTTCCAGTGTATATGTATGCCAGGTTAC |
| NOTCH2 | XM_013123808.1 | 7732-7831 | GCATTTATCTTCACTGAATATCTGTGCAGATTTAGGGGAGGACTCCACTCTGCTGATCTGTGACCATCGGACAAGTTCATGGAGATGCAAGATGAATCCA |
| NOX1 | XM_005072497.2 | 798-897 | CCCTGAGTCTTGGAAGTGGATCCTTGCACCGATCACTCTATATATCTTTGAAAGGATGCTTCGCTTTTATCGCTCCCAGCAGAAGGTTGTGATTACTAAG |
| NR3C1 | XM_005069186.2 | 1995-2094 | CTCATGGATGTTTCTCATGGCATTTGCCCTGGGTTGGAGATCATACAAGCAAGCAAGTGCAAACCTGCTGTGTTTCGCTCCTGATCTGATTATTAATGAG |
| NR4A2 | XM_005068973.2 | 1669-1768 | TATTTGTTCTTCGATTAGCGTACAGGTCCAACCCAGTGGAGGGTAAACTCATCTTTTGCAATGGGGTGGTCTTGCACAGGTTGCAATGCGTGCGTGGCTT |
| NT5E | XM_005073660.2 | 1208-1307 | CCTGATCTGTGATGCTATGATTAACAACAACCTCAGACACCCAGATGAAATGTTCTGGAACCACGTGTCCATGTGCATTTTAAATGGAGGTGGCATCCGG |
| NUP107 | XM_005081498.2 | 818-917 | CCAGCGAGAAGACTGTTGTGGAAGCTTTATTTCAGAGAGACTCACTGGTCCGACAGAGTCAGCTGGTGGTAGATTGGTTAGAAAGTATTGCCAAAGATGA |
| OAS1 | XM_013119795.1 | 574-673 | GAAATTGAGAAAGAGCTGTGCCAGTTACAGGGCGAGCAAAACTTTCCAGTGAAATTTGAGGTCTTCAGATCATGGGGGATCAACTCCCTTGGACTAATCT |
| OAS2 | XM_005079048.1 | 1140-1239 | AAAGGCTGTTGACATCATCTGTGCATTCCTTAAAGAGAACTGCTTCCGACATTCAAGCACCAAAGTTCTGAAGTCCGTCAAGGGAGGATCCACCACCAAA |
| OAS3 | XM_013119800.1 | 1273-1372 | AGCCAAAGGTGCCCGGTGTCTTGCATTTCCGGCTGATGTCAGCAGACAAGGAAAACTGGATGGATGTTAGCCTGGTGCCTGCCTTCGATGTCCTGGGACA |
| OASL | XM_013119728.1 | 367-466 | CTGGACCTCGGACTCGATAACCTGGGAGTGACAGAAGGATTCCCCAGTGGTCTCATCTTCACTTTGCGGACAAGGGAGACGTGGGAGCCAATCACTGTCA |
| OCLN | NM_001281666.1 | 104-203 | TGCACGTTCGGCCAATGCTGTCTCAGCCGGCATACTCCTTTTACCCAGAAGATGAAATTCTTCACTTCTACAAATGGACCTCGCCTCCGGGAGTGATTCG |
| PAI1 | XM_005080401.2 | 174-273 | CTGGTCTTTGGGAAAAGCTCGGCTTTACATCTTCAAGAGTCCCGCACAGCCCATCAGGCCACCGACTTCGGAGTAAAAGTGTTTAAGCATGTGGTCCAGG |
| PAK1 | XM_013116264.1 | 1433-1532 | CTGTTACTCCAACTCGGGATGTGGCTACATCTCCTATTTCACCTACCGAGAATAACACTGCTCCACCAGATGCTTTGACCCGGAATACTGAAAAGCAGAA |
| PANX1 | XM_005084033.2 | 1417-1516 | TGCATTTCAAGTCTGAAGGCTACAACGACTTGAGCCTCTACAACCTCTTTCTGGAAGAGAACATAAGCGAGCTCAAATCGTACAAGTGTCTTAAGGTGCT |
| PAX5 | XM_005078758.1 | 564-663 | CCCCAGTGCCGACACCAACAAGCGCAAGAGGGATGAAGGTATCCAGGAGTCTCCAGTGCCAAACGGCCACTCACTTCCCGGCAGAGACTTCCTGCGGAAG |
| PDCD1 | XM_005077183.2 | 172-271 | AACTGGTCCGAGGACCTTATGCTGAACTGGTACCGCCTGAGCCCCAGCAACCAGACTGTAAAACAAGCCGCCTTCTGCAAAGGGTTAAGCCAGCCCGTCC |
| PDCD1LG2 | XM_013110451.1 | 419-518 | TGTACACGGCAGAGTTCGGCAGCAACGTGAGTCTGGAGTGTGATTTTGACCGCAGTGAATGCACAGAGTTTGGAGAAATAAGAGCCAGCTTGCAGAAGGT |
| PDCD2 | XM_005083026.2 | 451-550 | TTTCTCTTCTGCTGTCGCGAGCCGCCGTGCTGCGCCGGCCTGCGAGTTTTTCGGAATCAGCTACCAAGGAAAAATGGGTTTTACTCCTATGAGCCGCCTT |
| PDGFA | XM_005080002.2 | 699-798 | GGAGACAAGCCTGAGAGCCCACAGGTCCCATGCCATTAAGCATGTGCCGGAGAAGCGGCCTGTGCCTATTCGCAGGAAGAGAAGCATTGAGGAAGCCATC |
| PDGFB | XM_013111327.1 | 1437-1536 | AGGAGACCTGTCTATTATATTCCACTCCAGCCAAGACTGCCCAGGAAGGTTGTGTGGGATGTGTGTTGCACATGCTCACACCCTGAATGTGGCCAATTTG |
| PDGFRB | XM_005074687.2 | 1413-1512 | GACCTTGGGTGACTCCAGCGCCGGTGAGATGGTTCTGTCTACTCGCAACGTGTCTGAGACCCGGTACGTGTCAGAATTGACCTTGGTGCGTGTGAAGGTG |
| PDK1 | XM_005064640.2 | 1161-1260 | GGTTATGGATTGCCCATATCACGCCTCTATGCACAGTATTTCCAGGGGGACCTAAAGCTGTACTCATTGGAGGGCTACGGGACTGATGCTGTTATCTATA |
| PECAM1 | XM_013113224.1 | 1241-1340 | ATTTTGGATCTGTTTTGCTCTGTCCCGGGCATACCTACAGCCAACTTCACCATCCAGAAGGAAAAGGTGATCTTGTCTCAGGATCAGAACTTCAGCAAGA |
| PIAS1 | XM_005075574.2 | 200-299 | AACTCTACAGGAGGCGGTTCCCTCAGAAAATTATGACGCCTGCGGACTTGTCTATCCCCAACGTACATTCAAGTCCTATGCCAGCGACTCTGTCTCCATC |
| PIGR | XM_005079862.2 | 856-955 | TTCCAGTGCTGATAAAAACAATGTTGACCTCCAGGTAATAGAGCCTAAGCCAGAGCTGGTTTATGGAGATCTGAGAGCCTCATTAGCTTTTAACTGTGAT |
| PIK3C2G | XM_013115494.1 | 2981-3080 | ATGCTGATCCGATGGGTGAAAACATCGGTGTTATTTTTAAGGCTGGGGATGATCTCCGGCAGGATATGCTTGTTCTGCAAATTATTCAAGTGATGGACAA |
| PLA2G2A | XM_013121230.1 | 392-491 | CTACAGTTGGAAGTACCTCATGGGTATCAAGAAATCTTGCGAAGGAGAGTCACCCAGCTGCTCAGAGAGCCGCTTTTTTTTCACATAGCCCAACCCAGTC |
| PLA2G2E | XM_005081046.1 | 224-323 | TGGGCTGTGAACCCAAGCTGGAAAAATACCTCTTCTCTGTTGGTCGAGACACCATCTTCTGTGGTGGCAGGACGACTTGCCAGCGGCAGACCTGCGAATG |
| PLA2G4A | XM_005073467.2 | 1802-1901 | CAGTGGGCTCACCTTTAACCTGCCGTATCCCTTGATTCTCCGACCTCAGAGAGGTGTGGATCTCATCATCTCCTTTGACTTTTCTGCAAGGCCAAGTGAC |
| PLA2G6 | XM_005066847.2 | 1686-1785 | GCATCCTGGCCCTGGCCATTCTGCACAGTAAGTCCATGGCCTATATGCGTGGCGTGTACTTCCGTATGAAGGACGAGGTGTTTCGGGGCTCACGGCCCTA |
| PLAU | XM_005083537.2 | 1039-1138 | GATGCTCCTTTTGGTTCAGACTGTGAGATCACTGGCTTTGGATATGAGAATGAAGGTGACTATATCAACCCAAAGAACCTGAAGATGTCTGTTGTGAAGC |
| PLCB1 | XM_005068490.2 | 1481-1580 | AAAATACCCACTGGAATCTGGGGTACCTCTTCCAAGCCCTATGGATTTAATGTATAAAATCTTGGTGAAAAACAAGAAGAAGTCCCACAAGTCATCGGAG |
| PLCG2 | XM_005073071.2 | 3017-3116 | TTTGGAAAACCCTGACTTCCGAGAAATCCGCTCCTTCGTGGAGACAAAGGCGGACAGCATTGTGCGGCAGAAGCCCATTGATCTATTGCGGTACAATCAG |
| PLK3 | XM_005072046.2 | 425-524 | GATCGAGTTGCACCGAGACCTACAGCACCGCCATATCGTTCGCTTTTCGCACCATTTCGAGGATGCTGACAACATATACATTTTCTTGGAGCTCTGTAGC |
| PMCH | XM_013111842.1 | 295-394 | CAAAGAACACAGGCTCCAAACAGAATCTCATAAATCATGGTCTGCCACTGAATCTGGCTCTAAAGCCTTACCTCGCTCTGAAAGGATCAGTAGCTTTTCC |
| PML | XM_013112977.1 | 1664-1763 | TGTCCTAGAAGAAACCCTCCTACTCACCAACAACCTTGTTACCAGCGACACAGGGGAAACAGAAGAGCGAATTGTGGTGATTAGCAGCTCAGAAGACTCA |
| POU2F2 | XM_013125974.1 | 1455-1554 | ACGCAAGAAGAGAACCAGCATTGAGACGAATGTCCGCTTCGCCTTAGAGAAGAGTTTTCTAGCGAACCAGAAGCCTACCTCAGAGGAGATCCTGCTGATC |
| PPARG | XM_013110341.1 | 317-416 | TGGCCATTGAGTGCCGAGTCTGTGGGGATAAAGCATCAGGCTTCCACTATGGAGTTCATGCTTGCGAAGGATGCAAGGGTTTTTTCCGAAGAACCATCCG |
| PPBP | XM_005068160.2 | 201-300 | TCTTGAATTGCGCTGTAGATGTGCTAATACAGTCTCTGGAATCCCACTCAGCAATATTTCCTTTGTGAATGTGTTCAGGCCAGGAGTCCACTGTGCCAAC |
| PPIB | XM_005075522.2 | 540-639 | CTCAGTTCTTCATAACTACAGTCAAGACGTCCTGGCTAGATGGCAAACATGTGGTTTTTGGCAAAGTTCTAGAGGGCATGGACGTAGTGAGGAAGGTGGA |
| PPP1R12B | XM_013115846.1 | 803-902 | GCTGCTGCCAAGGGTTACTCTGAAGTCCTCAGACTTTTAATTCAGGCTGGCTATGAACTCAATGTTCAGGATCACGATGGCTGGACTCCTCTTCATGCTG |
| PRDM1 | XM_005070414.2 | 836-935 | ATCCTAAAACTGGACTCGAATCCCACCAAAAGGAAGGACTTCTACTGTTCTGACATTTCATCCCTCACTTCAGAAAAGGACATGGATGGCTTCAGGAAAA |
| PRF1 | XM_005070839.2 | 1470-1569 | CGATGACCTTCTTGGTTCTTGTGACAGGGCTCCCAAGTCTGGCTACCACAGGACGACCTGTAGCCTGAACCATGGCAGTGTGACGTTTGTCTACAATATT |
| PRG2 | XM_005065081.2 | 314-413 | AGGACACAATGAGTCTGATGGGCATCCCTGGATGTAAGACCTGTCGCTTCCTGCTGGTGAGGAATCCTCGGCCGTTTGACAACGCTCAGTCAGTTTGCCG |
| PRKCA | XM_005069988.2 | 649-748 | AAACAAGGCTTCCAGTGCCAAGTTTGCTGTTTCGTGGTCCACAAGAGGTGCCATGAGTTTGTTACATTCTCCTGTCCGGGTGCAGACAAGGGACCCGACA |
| PRKCB | XM_013111539.1 | 1276-1375 | GGTTGTGGACCTGAAGGGGAACGAGACATTAAAGAACATGCATTTTTCCGGTATATTGACTGGGAGAAACTTGAACGCAAGGAGATTCAGCCACCATATA |
| PRKCD | XM_013124593.1 | 1783-1882 | CCACATTCAGGACAAAGGCCGCTTCGAACTCTACCGGGCTACGTTTTATGCAGCTGAGATCATTTGCGGGCTACAGTTTCTACACAGCAAAGGCATCATT |
| PSMB5 | XM_005139340.2 | 386-485 | GTTGGCTCGACAGTGTCGAATCTATGAGCTTCGAAATAAGGAACGCATCTCTGTCGCAGCGGCCTCCAAACTTCTTGCTAACATGGTCTATCAGTACAAA |
| PSMB7 | XM_005082082.2 | 494-593 | TATCCTCATGGATCAACTGATAAATTGCCTTATGTCACCATGGGTTCTGGCTCCTTGGCAGCAATGGCTGTGTTTGAAGATAAGTTTAGGCCTGATATGG |
| PSMB8 | XM_005086859.2 | 392-491 | AGGTCCTTTGGTGGTGACCAAGAAAGGAATGTTCAGATTGAGATGGCCCACGGCACAACCACACTAGCCTTCAAGTTCCAGCATGGAGTCATCGTGGCCG |
| PSMB9 | XM_005086860.2 | 461-560 | CGACAGCCCTTTGCCATCGGTGGTTCTGGCAGCACCTACATTTACGGTTATGTGGACTCAGCATATAAAGCAGGCATGACCCCTGAGGAGTGCAGGCGCT |
| PSMC2 | XM_005080885.2 | 430-529 | TCAATGTGAAGCAGTTTGCCAAGTTTGTGGTGGATCTCAAGGGTCTTGAAAATTGTGGGGCTCGAATGGTTCGTGAGCTTTTTGAAATGGCCAGAACAAA |
| PSMD7 | XM_005073019.2 | 495-594 | TCCTACCGAAGCTTACATTTCAGTGGAAGAAGTTCATGATGACGGAACACCAACATCCAAAACTTTTGAGCATGTGACCAGTGAAATTGGGGCAGAGGAA |
| PTAFR | XM_013120133.1 | 1306-1405 | GAGTAACTGCACTGGCAGCTTGTTGGAAAATACCAAACTTGAATGTTCCTTTTCACCCATTTTTGGGTTAACAACTAGCGACTGTGGCTGATTGGCTCAT |
| PTEN | XM_005063688.2 | 901-1000 | TACAATCTATGTGCTGAAAGACATTATGACACCGCCAAATTTAACTGCAGAGTTGCACAGTATCCTTTTGAAGACCATAACCCACCACAGCTAGAACTCA |
| PTGDR2 | XM_005063275.2 | 116-215 | ACATCACACAGAAGCTGCTCTGTCCACTCTTGGAGGAGATGATCCATCTTCCAAGCCACAGCAACTCTAGCATCCGCTATATTGACCACGTGTCGGTGCT |
| PTGER1 | XM_005087553.2 | 676-775 | CTTCGCCTGTATGCTGCAGGACGTTCGCCTGCTGGTGGGGCGTGTCATTTCCTGGGCGGCTGCATGGTTTTCTTCGGCCTGTGCCCACTTTTACTTGGCT |
| PTGER2 | XM_005085790.2 | 1221-1320 | TTCTACCCAGTCCAGTTCCAGCAAACAGACTGACCTTTGTGGACAGTTATGAGGACACACTTCATGAGGGACATCTGCAAAAGCCTTTGAATGGTTTCCT |
| PTGER3 | XM_005083474.2 | 1036-1135 | AATTCCTTTCTAATTGCAGTGCGCCTGGCTTCGCTGAACCAGATCTTGGACCCCTGGGTTTACCTGCTGCTGAGAAAGATCCTTCTTCGGAAGTTCTGCC |
| PTGER4 | XM_005077978.2 | 1371-1470 | CGGGCGCGGAGATCCAGATGGTCATCTTACTCATCGCCACCTCCCTGGTGGTGCTCATCTGCTCCATTCCGCTCGTGGTGCGAGTGTTCATCAACCAGTT |
| PTGFR | XM_005083635.2 | 320-419 | AGCAAACTCTACCTGTCAGATGCAAGACCGGCTTTCAGTGTTTTTCTCAGTAATCTTCATGACAGTGGGGATCTTATCTAACAGCCTGGCCATCGCCATT |
| PTGIR | XM_005086459.2 | 1989-2088 | CTCCTCTTAGAACTGGACCTCAATTCACCGTCTTCGGAACATCCACAAAGATTTTACTCATCCATTCAACAAACTCCTATGGGACCCTGAAGTCAGCAAG |
| PTGS1 | XM_013121983.1 | 1019-1118 | GGATGATGAGCAGCTCTTCCAGACCACTCGTCTCATCCTTATAGGGGAAACCATCAAGATTATCATCGAGGAGTATGTGCAGCACTTGAGTGGCTACTTC |
| PTGS2 | XM_005073469.2 | 1256-1355 | TTCAAACAGTTCCTCTACAACAACTCCATTCTCCTCGAGCACGGACTCGCCCAGTTTGTTGAGTCATTCACCAGACAGATTGCTGGTCGGGTTGCTGGGG |
| PTK2 | XM_013124760.1 | 1762-1861 | TGCACGCTTGGAGAGCTGAGGTCATTTTTGCAAGTAAGGAAATACAGCTTGGATCTGGCATCTTTGATCCTATATGCCTATCAGCTTAGCACAGCACTCG |
| PTPN2 | XM_005074634.2 | 290-389 | CGAAATGAGTCCCATGACTATCCTCATAGAGTGGCCAAGTTTCCAGAAAACAGAAATCGAAACAGATACAGAGATGTAAGCCCATATGATCACAGTCGTG |
| PTPN22 | XM_013118092.1 | 1303-1402 | GCGCCAGTGGTTCGGACCAAATCAACTCCTTTTGAACTAATTCAGCAGAGAAAAACAAATGAGTTGGACATGGAAGACGGTTCTTTGCTCTTGGGATCTC |
| PTPRC | XM_013115831.1 | 1724-1823 | CCCTATGATTACAACCGTGTTGAACTCTCTGAAATAAATGGAGATGCAGGGTCCACCTACATAAATGCCAGCTACATTGATGGCTTCAAAGAACCAAGGA |
| PTX3 | XM_005077938.2 | 896-995 | GTCACTCTGTTCCTGAGGGTGGACTCCTACAGATTGGTCAAGAAAAGAATGGTTGCTGTGTAGGTGGGGGCTTTGACGAAACATTAGCATTTTCTGGAAG |
| PYCARD | XM_005064325.2 | 360-459 | AGCAGGAGCAGCACATTTCGTTGACCGGCACCGGCAAGCACTCATTGCCAGAGTCACAGACATCGATGGCGTGCTGGATGCCCTGTATGGCTGTGTGCTG |
| RAC1 | XM_005079946.2 | 365-464 | TTATGGTAGATGGAAAGCCAGTGAATCTGGGCTTATGGGATACAGCTGGACAAGAAGATTATGACAGATTGCGTCCCCTCTCCTATCCGCAAACAGATGT |
| RAF1 | XM_005066112.2 | 1306-1405 | CACTACCTTCTTTGACAATGCGTCGGATGCGAGAGTCTGTTTCCCGGATGCCTGCTAGTTCCCAGCACAGATACTCCACACCTCATGCCTTCACTTTCAA |
| RAG1 | XM_013120728.1 | 1881-1980 | CATGAGAATTACCATAGATCACGACTCGAAGAATGTGAAGGTGTTTGAGGAACCCAAACCCAATTCTGAACTGTGTTGCAAGCCATTGTGTCTTATGCTG |
| RAG2 | XM_013120950.1 | 400-499 | CCCGAAGCCAGATATGGTCATTCCATTGATGTGGTGTATAGTCGAGGGAAAAGTATGGGTGTTCTCTTCGGAGGACGATCATACATGCCTTCTACCCAGA |
| RAPGEF2 | XM_005076139.2 | 3288-3387 | CGTGAAGGATCTGCCACCTTTCGGCATAAACTCTCCTCAAGCGTTAAAGAAAATCCTGTCCTTGTCTGAAGAAGGGAGTTTGGAGCGACATAGGAAACAA |
| RELA | XM_005063992.2 | 1746-1845 | GCTCCTGAACCAGGGTGTGTCCATGTCTCACCCCACAGCTGAGCCCATGCTCATGGAGTACCCCGAAGCTATAACTCGGCTAGTGACAGGGTCCCAGAGG |
| RELB | XM_013125430.1 | 752-851 | GCGCCGCCTGGAACCCGTCCTCTCTGAGCCCGTCTATGATAAGAAGTCCACCAACACATCAGAGCTGCGGATTTGCCGAATCAACAAGGAGAGCGGGCCC |
| REPS1 | XM_005065845.2 | 1719-1818 | AAGGCCACAGCCCTCTCATTCTAGATCATCATCCCTAGATATGAATCGGACCTTTGCAGTTACCACAGGACAGCAACAGGCTGGAGTAGTTGCCCACCCT |
| RHOA | XM_005088273.2 | 376-475 | GCTCTGCAAGCCAGACGTGGGAAGAAAAAATCTGGGTGCCTCATTTTGTGAAGCCTTGTGCAAGCACAGCCCTCATGCGGTTAATTTTGAAGTGCTGTTT |
| RIPK1 | XM_013110581.1 | 1679-1778 | GTACCATTCTGGAACAACGGATTATATAGTCAGCATGGGTTTGGAACTTCAGGTGCAGGAGTTTGGTATGGGCCAAATGCAAGCCAAATGTATAATACTT |
| RIPK2 | XM_005066813.2 | 892-991 | GGTCACCAATCCTTTGCAGATCATGTACAGTGTGTCACAGGGACATCGGCCTGACACCAGTGAAGAGAATTTGCCATTTGACATACCTCATCGAGGTCTC |
| RNAseL | XM_005071446.2 | 1339-1438 | TGCTGTTGAGCACAACCTGAATGAAATAGTTCAGTTACTGTGTGATAAGGGAGCCAGTACCGAGTATGGGGATCTTGTTGAGATAGCCAGGCGAAATTAT |
| ROCK2 | XM_013124377.1 | 2731-2830 | CTTGCATATTGGTATGGATAGTTCCAGTATAGGCAGTGGACCAGGGGATGCTGAGCCTGATGATGGATTTCCAGAGTCAAGATTAGAAGGATGGTTGTCA |
| RORC | XM_005087689.2 | 1361-1460 | AGAGAAAAGAAGAGTGGAGCATCTGCAATACAATTTGGAGCTGGCCTTCCATCATCATCTCTGCAAGACTCATCGCCAAGGCCTCCTAGCCAAGCTGCCA |
| ROS | XM_005073341.1 | 2632-2731 | ATTTCCCAGAATGCACTGATGTACTACAGTGGTCGGCTCTTCTGGATCAATGGGTTTAGGATTATTATAGCACAGGAAATAGGTCAGAGAACCAGTGTGT |
| RPL18 | XM_005084699.2 | 154-253 | TGGCCAGAAGAACCAACTCCACCTTCAATCAGGTTGTGCTGAAAAGGTTGTTTATGAGTCGCACTAACCGGCCGCCTCTGTCCCTGTCCCGGATGATCCG |
| RPS6KA5 | XM_005068407.2 | 2014-2113 | TATACAATGCTGTCAGGGCAGGTACCATTCCAGTCTCATGACAGAAGCTTAACGTGCACCAGTGCAGTAGAAATCATGAAGAAAATTAAAAAGGGAGATT |
| RRAD | XM_005078661.2 | 573-672 | ACGCAGAAGCCGCAGGGCACACATATGATCGTTCTATCACTGTGGATGGAGAAGAGGCATCACTCATGGTCTATGACATTTGGGAACAGGATGGGGGCTG |
| RSAD2 | XM_005082038.2 | 652-751 | AGCAATGAAGAATTTGAAGCATTCCTAGAACGTCACAAAGAGGTGTCCTGTTTGGTGCCCGAATCTAACCAGAAGATGAAAGACTCCTACCTTATTCTGG |
| RTP4 | XM_005071699.2 | 362-461 | GACAGCATCCAAAGGATTCTGGAGAACCTAGTTAGTTATATTTTGCGGAGATACTATAGACATAGCTCGAAGAAGCCACCATCAACCTTGTATGAGAGGG |
| RUNX1 | XM_013116148.1 | 544-643 | ATCACAGTGGACGGACCGCGAGAACCCCGAAGACATCGGCAGAAACTAGATGAGACCAAGCCGGGGAGTTTGTCCTTTTCCGAGCGGCTCAGTGAACTGG |
| S100A8 | XM_005080310.2 | 73-172 | TGGAAAACATAGTTAATGTCTACCATCAGTATTCTGGGACGAAGGGGAACCACCATGCCCTCTACAGGGATGATTTGAAGAAACTGCTCACGACTGAGTG |
| S100A9 | XM_005080242.2 | 274-373 | CTGAGCTTTGAGGAGTTTGTGATCCTGATGGCAAAGATGGTCCATGCCACCCATGAGAAGATGCACGAACATAACTCACGTGGGCATAACCACAGCCACG |
| S1PR1 | XM_005077098.2 | 873-972 | CCCAGTGGTTTGTGCGGGAAGGGAGTATGTTTGTGGCTCTGTCTGCCTCGGTGTTCAGCCTACTTGCCATTGCCATCGAGCGCTACATCACCATGCTGAA |
| SAMD4A | XM_005085801.2 | 1083-1182 | CTATGCTTTGATCCACCCGGCCACTTCCTTAGAAGACCGAAGTGCTCTAGCCATGTGGCTCAACCATTTGGAGGACCGCACATCGACTAGCTTTGGCAGC |
| SELE | XM_005071368.1 | 988-1087 | TCATCCTGCAACTTCACCTGTAAGCAAGGTTTCGTGTTGGAGGGACCAGCCCAAGTTGAATGCAGTGTAGAAGGGCAGTGGATACCACAGTTCCCAGTGT |
| SELL | XM_013114268.1 | 1113-1212 | CTCGGGGCTGGCATTTATCATTTGGCTGGCAAGGAGGTTAAAAAAAGGCAGGAAATCTCAGGAAAGGAAGGACGATCCATACTAACTCATCCTTTGTGAA |
| SELPLG | XM_013119725.1 | 836-935 | GCCTATTGACTCTGCCATCTTCTTTGGGCCATCTGTGACTCACAAGAATAATGATGTGGCAACTGACCACTTAAATGACAATGCTTTGAAGAACGGGCTG |
| SEPT4 | XM_005075779.2 | 1029-1128 | GCGGGTCAACATTGTGCCTATCTTGGCTAAGGCAGATACACTGACACCTCCTGAAGTGGACCGAAAGAAATGCAAAATCCGGGAGGAGATTGAGCACTTT |
| SERPING1 | XM_013122156.1 | 1189-1288 | AAAACTGGGAAGGCATCTTTCCGCTACAAAAACTCTGTGATTAAAGTGCCCATGATGAGTAGCAAAAAGTACCCGATGGCCCATTTCAGTGACCAGACTT |
| SH2D1A | XM_005076829.2 | 180-279 | AACAGGTTCTTGGAGTGCTGAGACAGCACCTGGAGTACACAAAAGATTTTTCAGGAAAATAAAGAATCTGATTTCAGCATTTCAGAAGCCAGATCAAGGC |
| SHB | XM_005078744.2 | 899-998 | CAGAGTTCCAGAGGCAGGAGAGCGTTCGGTCCCAACACAAAGGCATCCAGTTATATGACACGCCTTATGAGCCCGAAGGCCAGAATGTGGACTCAGACTC |
| SHC1 | XM_005080186.2 | 1702-1801 | CCAACGACAAAGTCATGGGACCTGGGGTTTCCTACTTGGTTCGGTACATGGGTTGTGTGGAGGTCCTGCAGTCAATGCGAGCCCTTGACTTCAATACCCG |
| SHIP | XM_013122102.1 | 1445-1544 | ACTCAAGCACTCCCTGCAAGAAGTCACCAGCATGACGTTTAAAACAGTTGCCATCCACACACTCTGGAACATTCGCATAGTTGTGCTTGCCAAGCCAGAG |
| SHP2 | XM_005080652.2 | 1285-1384 | GAGCAAATGCGTCAAGTACTGGCCTGATGAGTATGCACTCAAAGAATATGGGGTCATGCGTGTTAGGAATGTCAAAGAAAGTGCCGCTCATGACTATACC |
| SIGIRR | XM_005064222.2 | 1855-1954 | ATTGGGCAATGGAAGCCACTTCAGCCTCCATCAGGATTTTTGGGTCAGTGCCAACTTCTCAAAGGTTGTGTCCAGTGTTCTAGTGCTCAACTTGACCAAT |
| SIGLEC1 | XM_013112266.1 | 2705-2804 | TGAGGATTCAGGAAGCTACCACTGTGAGGCCACAAATGTTCTCGGATCAGCCAACAGTTCACTCTTCTTCCAGGTCAGAGGGGCCTGGGTTCAGGTTTCA |
| SIT1 | XM_005078796.2 | 75-174 | GATCCCCTCCATAACTTTTGCATGGGGACTGTGGGTCCTCTTAGGGCTTGTGACAGTGATGCTTCTCATTTTGCTGACTGCATTCTTGTCCCAGTGGACC |
| SITPEC | XM_013119366.1 | 583-682 | AGACAGAGTTTCTGCTGACTCAGATATTTGGACGCATAAGTTACCCCATGCTCAAGTTCCTGCGGATGAAGCTGTGGCTCACCCGCTTCAAGAATATCAA |
| SKI | XM_005079242.2 | 277-376 | CCCGGGCCCTTCTTCATGCCGTCGGATCGCTCCACCGAACGCTGTGAGACCGTGCTGGAAGGGGAGACCATCTCTTGTTTCGTAGTGGGAGGCGAGAAGC |
| SLAMF1 | XM_005078217.1 | 580-679 | CTCCTGTACATCACCCTTAGCAACCAGCATCAGGACAGCATCTACAACTGCACTGCAAGCAACCCTGTCAGCAGTCACTCTCGGACCTTTGACCTATGGC |
| SLAMF6 | XM_005078261.2 | 307-406 | TCACGAAGGAGAAGCATCACAAGTCACTACAATCCTTATCATCCAGCTAAATAAGTCTGGGACTCCACAAATCATACACAGTGATCTAAAGATGCGAGAG |
| SLAMF7 | XM_013119167.1 | 307-406 | CGTGCAGAGATTCACAATACATCACTTCAGTCTCCCTTCTCCCAGGAGTTCGTGCTGTGCGTCTATGAGAGTCTGTCAACACCAAAAGTCACCATGGATG |
| SLC2A1 | XM_005083859.2 | 2140-2239 | CCCACATAGGGGTCGGGCTCCATTTTAGGATTCGCCCATTCCTATCTCTTCCTTCCCAACCACTGAATTAATCTTTCCTTGCCTGAGACCAGTTGGAAGC |
| SLC39A8 | XM_013121936.1 | 1566-1665 | CGGATTTCACCTTCTTCATGATCCAGAACGCCGGGATGTTGACTGGGTTCACCGCCATCCTGCTCATCACGTTGTATGCGGGGGAAATCGAGTTGCAGTA |
| SMAD2 | XM_005076458.2 | 845-944 | ACAGCTTGGATTTGCAGCCAGTTACTTACTCGGAACCTGCATTTTGGTGTTCAATAGCTTATTATGAACTAAACCAGAGGGTTGGAGAGACCTTCCATGC |
| SMAD3 | XM_005075564.2 | 846-945 | CATGAAGAAGGACGAAGTGTGCGTCAATCCCTACCACTATCAGAGAGTAGAGACACCAGTTCTACCTCCAGTGTTGGTGCCACGCCATACCGAGATCCCG |
| SMAD5 | XM_005078393.2 | 540-639 | ACCACTATAAGAGAGTGGAAAGTCCAGTCTTACCTCCAGTGTTAGTGCCTCGCCACAACGAGTTCAACCCACAACACAGCCTTCTGGTTCAGTTTAGGAA |
| SMAD7 | XM_005076452.2 | 1242-1341 | GACAACCCGGACTCTAGGACGCTGTTGGTGCATAAGGTGTTCCCTGGCTTCTCCATCAAGGCTTTCGACTATGAGAAAGCATACAGCCTGCAGCGACCCA |
| SMPD3 | XM_005076321.2 | 1689-1788 | TGCAATTTCAAGTGTCTCAACAGCGGTCTCTTCTTTGCCAGCCGCTACCCTGTCATGGATGTGGCCTATCACTGTTACCCCAATGGGTGCAGCTTCGATG |
| SOCS1 | XM_005084570.2 | 640-739 | CGCTGCAGGAGCTGTGTCGCCAGCGCATCGTGGCCGCCGTGGGTCGCGAGAACCTGGCGCGCATCCCTGTTAACCCGGTACTCCGCGACTACCTGAGTTC |
| SOCS3 | XM_013113173.1 | 732-831 | ACCAGCGCCACTTCTTCACGCTGAGCGTCAAGACCCAGTCAGGGACCAAGAACCTACGCATCCAGTGCGAGGGGGGCAGCTTTTCGCTGCAGAGCGACCC |
| SOS1 | XM_005073625.2 | 940-1039 | AGCAGAGGAACTAGCATTTGACCCATATGAATCATATGCTCGGGATATTTTACGGCCAGGATTCCATGACCGTTTCCTTAGTCAGTTATCAAAGCCTGGG |
| SP1 | XM_005067311.2 | 1362-1461 | AAACCTTCACAACTCAAGCTATTTCCCAGGAAACCCTTCAGAACCTCCAGCTTCAGGCTGTTCAGAACTCTGGTCCCATCATCATTCGGACACCAACAGT |
| SPP1 | NM_001281641.1 | 548-647 | TGACAGTTTGGCTTATGGACTACGGGCAAAGTCTAGGAAGTTCCACATTTCTGATGACCAGTATCCTGATACTACAGATGAAGACCTTAGCTCCCACATG |
| SRC | XM_005084592.2 | 1450-1549 | GCGAGCTGCCAATATCCTGGTTGGGGAGAATCTGGTGTGTAAGGTGGCTGACTTTGGTCTGGCCCGGCTCATAGAAGACAACGAATACACAGCCCGGCAA |
| SRF | XM_005072345.2 | 938-1037 | TATACCTTCGCTACCCGCAAACTGCAGCCCATGATCACCAGTGAGACCGGCAAGGCACTGATTCAGACCTGCCTCAACTCGCCAGACTCTCCGCCCCGCT |
| STAT1 | NM_001281685.1 | 721-820 | AGAAAGGAGATAGTTCACAAGATAAGAGAGTTGTTGAATGCCATCGAGCTCACACAGAACAACCTGATTAACGATGAGTTAGTGGAGTGGAAGCGAAGAC |
| STAT2 | NM_001281622.1 | 956-1055 | GTCACATGCTTAAGTATGACGGGGACATGTTTAGCAAAGGGGTGGACCTGCAGAATGCCCAAGTCACAGAGTTACTACAACGCCTGCTCCAAAGGTCCTT |
| STAT3 | XM_013113246.1 | 1168-1267 | GCCAATTGTGACGCCTCCTTGATTGTGACTGAGGAGCTGCATCTGATCACCTTCGAGACTGAGGTGTACCACCAGGGCCTCAAGATTGATCTAGAGACCC |
| STAT4 | XM_013123968.1 | 1122-1221 | ATTTGTGCTTTGTGGAACTCAAGTCAAAGCCATGTCCATCGAGGAATCCTCCAATGGGAGCCTCTCAGTAGAATTTAGACATTTGCAACCAAAGGAAATG |
| STAT5A | XM_005070267.2 | 146-245 | AAGTTCACGGTCCTATTTGAGTCTCAGTTCAGTGTTGGCAGCAATGAGCTGGTGTTCCAGGTGAAGACGCTGTCCCTCCCTGTGGTTGTCATCGTTCACG |
| STAT5B | XM_013126205.1 | 2421-2520 | ATGTCCCTGAAACGAATCAAGAGGTCTGATCGCCGTGGTGCAGAGTCGGTGACGGAAGAGAAGTTCACGATCCTGTTTGACTCACAGTTCAGCGTGGGTG |
| STAT6 | XM_005079747.2 | 1588-1687 | TACGTCACAAGCCTTCTCCTCAACGAGCCAGACGGAACTTTCCTCCTCCGCTTTAGCGACTCTGAGATTGGTGGCATCACCATTGCCCATGTTATCCGGG |
| SYK | XM_013110528.1 | 1532-1631 | GGATAAGAACATCATCGAGTTGGTTCACCAGGTTTCCATGGGAATGAAGTATTTGGAAGAATGCAATTTCGTGCACAGAGATCTGGCTGCGAGGAACGTG |
| SYT17 | XM_005075623.2 | 1532-1631 | GAACTGGAAAATGCCAGCCTAGTATTCACAGTGTTTGGTCACAACATGAAAAGCAGCAACGACTTCATCGGGAGGATCGTCATTGGCCAGTATTCCTCCG |
| TAB1 | XM_013111072.1 | 817-916 | GTGGATCGTGTAAAGCGTATCCACAGTGATACCTTTGCCAGTGGCGGGGAGCGTGCCAAGTTCTGCCCACGGCATGAAGACATGACCCTGCTGGTGAGGA |
| TAB2 | XM_005065875.2 | 1307-1406 | ACAGCCAAACCTTAAATAGAAATCAGCCCACTGTTTACATAGCTGCCAGTCCGCCAAGTACTGATGAGATGATCTCCCGTAGTCAACCTAAGGTCTATAT |
| TAGAP | XM_005065924.2 | 580-679 | CCCGGTGCATCTCCTGGCTGTGGTCTTCAAGGACTTCCTCCGAGGAATCCCTCTGAAACTGCTGTCCTGTGACCTCTTTGAGGAATGGATGGACGCCCTG |
| TAL1 | XM_013114861.1 | 694-793 | CTCAGCCAGCCACTGGCCTCTCTCGGCAGTGGGTTCTTTGGGGAGCCAGATGCCTTCCCTATGTTTACCAACAACAACCGGGTGAAGAGGAGGCCCTCCC |
| TAP1 | XM_013125846.1 | 1042-1141 | CACATGCACAGCCGTGCGCATGGAGAGGTGTTTCGGGCTGTCCTTCACCAGGAGACAGGGTTTTTCCTGCAGAACCAAGCAGGTTCCATCACGTCTCGGG |
| TAP2 | NM_001281603.1 | 182-281 | GTGTGGGGACCTTTCCGCCCTTGCTCTGCCTGGCTACCCCGCTGTTTTTCTCTCTAAGAGCGCTGGTGGGAGGCACCATGAGTTCCCCAGCAGTCAGAGT |
| TAPBP | XM_005084920.2 | 282-381 | CCTCTGCCCCTGCTCATCGCTGTGGCTTTGGGCCTGGTGCCCGCCGTCTCTGCTGGGCTGGAGGCGATTGAGTGCTGGTTCGTGGAAGATGCAGGTGGGG |
| TBK1 | XM_005079789.2 | 1065-1164 | CGGATGATAATCCATGTGTTCTCACTGCAACAGATGACAGCACATAAGATTTACATCCATAGCTACAATACGGCTGCTGTGTTCCATGAACTGGTCTATA |
| TBX21 | XM_005075903.2 | 525-624 | CGGCGAATGTTCCCATTCCTCTCTTTCACTGTGGCTGGACTGGAGCCCACAAGCCATTACAGGATGTTTGTGGATGTGGTCTTGGTGGACCAGCACCACT |
| TBXA2R | XM_005083260.2 | 726-825 | CTCCTGGGCGGCGCGTCCGTCGGTCTGTCCCTCCTGCTCAACACCGTGAGCGTCGTCACCCTCTGCCGCGTCTACCACGACCGCGAGGCCGCGCGCCAGC |
| TCF4 | XM_005074614.2 | 1217-1316 | AATGTTGGGCAATTCCTCTCATATTCCGCAGTCTAGCAGCTACTGTAGCCTGCATCCACATGAACGTTTGAGCTACCCATCCCACTCCTCAGCAGACATC |
| TCF7 | XM_005067778.2 | 281-380 | GGGAGCGCCCGAGGCCCTGGGACGAGAGCACACTTCGCAGAGACTTTTCCCGGACAAACTTCCAGAGTCCCTAGAGGACGGCCTGAAGGCCCCGGAGTGT |
| TFRC | XM_005071554.2 | 1955-2054 | ATTCTGTCATTTGTGAAGGAGCTGAACCAATTCAGAGTGGATATCAAGGCAATGGGTCTGAGTCTACAGTGGCTGTACTCTGCTCGTGGAGACTTTTTCC |
| TGFB | AF046214.1 | 90-189 | GGGCTACCACGCCAACTTCTGTCTGGGGCCCTGTCCCTACATTTGGAGCCTGGACACACAGTACAGTAAGGTCCTTGCCCTCTACAACCAACACAACCCG |
| TGFB1 | XM_013125593.1 | 1035-1134 | CTGCGTCTACAGAGATTCAAGTCAAATGTAGAACAACACGTGGAACTCTACCAGAAATACAGCAACAATACCTGGCGTTACCTTGGTAACAGACTCCTGT |
| TGFB2 | XM_005082438.2 | 747-846 | CCATCCCGCCCACTTTCTACAGACCCTACTTCAGAATTGTCCGCTTCGATGTCTCGATGATGGAGAAGAACGCTTCTAATTTGGTGAAGGCAGAGTTCAG |
| TGFB3 | XM_005084008.2 | 1408-1507 | CCAAAGGGATTACCTCTAAGGTTTTTCGCTTCAACGTGTCCTCAGTGGAGAAAAATGGAACTAATCTGTTCCGGGCAGAGTTCCGGGTCTTGCGGGTGCC |
| TGFBI | XM_005078391.2 | 1867-1966 | CTCGAAAAACAATGTAGTGAGCATCAATAAGGAACCTGTTGCCGAAACTGACATCATGGCCACGAATGGTGTGGTCTATGCCATCAACAGTATTCTGCAA |
| TGFBR1 | XM_005078729.2 | 495-594 | CTGCATTGCACTTATGCTGATGGTCTATATCTGCCATAATCGCACTGTCATTCACCATCGCGTGCCAAATGAAGAGGACCCATCACTAGATCGCCCTTTC |
| TGFBR2 | XM_005075040.2 | 866-965 | CTGACCTGTTCCTGGTCATTATCCAAGTGACCGGCGTCAGCCTCCTGCCTCCGCTGGGAATCGCCATAGCTGTGATCGTCATCTTCTACTGCTATCGTGT |
| THBD | XM_005072559.2 | 1393-1492 | CACAGGTGTGAATTGTTCTGCAATGAAAGCTCATGCCCAGCTGACTGTGACCCTTACGCTCCTGACTTTTGCTATTGTCCTGAGGGCTTCATCCTGGATG |
| THBS1 | XM_005064519.2 | 3201-3300 | TACGATGAGTTCAACGCCGTGGACTTCAGTGGTACCTTCTTCATCAACACTGAGAGAGATGACGACTATGCTGGCTTTGTGTTCGGTTACCAGTCCAGCA |
| THY1 | XM_005069414.2 | 219-318 | ACCTTCGTCTGGACTGTCGTCATGAGAATAACAGCCTCTTGACCATCCAACACGAGTTCAGCCTGACTCGAGAGAAAAAGAAGCATGTGCTGGCAGGCAC |
| TICAM1 | XM_005088421.2 | 1865-1964 | GCCATCAACCACTCAGGGTATACTATCCTCCTCCTGACCACCAACTTTGATTGTCGCCTGAGCCTGCACCAAGTCAACCATGCCCTTATGAACAGCCTCA |
| TICAM2 | XM_013112742.1 | 423-522 | TGTCAATGGGTCTGCCTGGACGATCTTATTACTGACTGAGAACTTTCTGAGAGACACCTGGTGTAACTTTCAGTTCTACACCTCGCTGATGAATTCTGTG |
| TIGIT | XM_005074799.1 | 83-182 | TAGAGACAAACAGGAACATTTCCGCAGAGGAAGGAGGTTCTGTCATCTTACAGTGCCTATTCTTCTCCAACACTTCTGAAGTGACTCAGGTCAACTGGGA |
| TIMM2 | XM_005069851.2 | 633-732 | GGTCTCGCTGGACGTTGGAGGAAAGAAGGAGTATCTAATTGCAGGAAAGGCAGAAGGAGACGGCAAGATGCACATTACCCTCTGTGACTTCATTGTGCCC |
| TIRAP | XM_005085836.2 | 1599-1698 | GCGGAAGAGGCAGCAGGTCTCAGGTCCTTCACATCCACACTACTGTAGTTCTGTCCCAGAGGCGTCAACTCACTTTATAAGGCTGGGTGAAAGGCACCTC |
| TJP2 | XM_013116110.1 | 1014-1113 | CTTCATGAGGGGGACATAATTCTCAAGATCAATGGGACTGTTACTGAGAACATGTCTTTAACTGATGCTCGGAAGCTAATAGAAAAGTCTAGAGGGAAGC |
| TLR1 | XM_005074382.2 | 1134-1233 | CAAAGCGTCACATTGTCTGGAACACACATGCTTCACATGGTTTGCCCATCCCAAGCTAGCCCATTTCTGCATTTGGACTTCTCAGATAACCTCTTAACAG |
| TLR10 | XM_005139227.2 | 1068-1167 | GCCTTCTTTGAAAACCCTCATTTTGAGGGGCAACAAGCTGGAGAGTCTTTCTTTGGTGAGCGTCTTTGCCAACAGCACACCCTTGCTTCACTTGGATCTG |
| TLR11 | XM_005087494.1 | 190-289 | CTGCTTCCTAACCTTTCCTCCTATATCCGGTTCTGCCCATTGGCCCCGGGACTGCACCTCTTGGCATCTTGCTCGAATGTTAAAGACTTGGCTCACACCC |
| TLR2 | XM_005080078.2 | 798-897 | ACGATGAAAAAGCTGGCATTCCGAGACACAGATATCACTGACGAGAGTTTTAATGAACTTTTGAAGCTGCTGCGTTACACTCCAGAACTGTCAGAGGTGG |
| TLR3 | XM_005066656.2 | 1181-1280 | AGAATCTCTCTCTGGCTAACAACCAGCTTCTTGCAACCAAAGATACCACATTCTCCGGACTGAAGCAGACAAATCTCACCTCGCTTGATCTTTCCTACAA |
| TLR4 | XM_005075127.2 | 1525-1624 | TGTTCTTACTCTGATTTGGGAACAAGAAGCCTGAAACACTTAGACCTCAGCTTCAACGGTGTCATCAGGATAAATGCAAACTTCATGGGTTTAGAAGAGC |
| TLR5 | XM_005078118.1 | 1608-1707 | TAGCGGCTTGGTTTCGTTAAGGTTGCTCAGTCTTAATGCTAACAGGCTGACCACTCTCTCTCCCGGCAGTTTACCTGCTAACTTAGAGATTCTTGATGTA |
| TLR6 | XM_005139225.2 | 1323-1422 | TGAGTTTCTTGGGGTTAAGCGCTACAAAGTTCCGGCATCTGGATCTGCTCCCAGTCGCTCACTTGCACCTAAACTACATTCTTCTGGGCCTGGTGGGTTA |
| TLR7 | XM_013116406.1 | 1568-1667 | AGCCATTAAAAGAGCTGCGGTACTTAGACTTCTCGAACAACAGGCTTGATTTACTCTACTCAACAGCTTTTGAAGAGCTCCACAATCTCGAAGTTCTGGA |
| TLR8 | XM_005074182.1 | 772-871 | AATTGCACACCTTGCCACGGAAACGCTTCTATCCACATACATCCTCTCGCTTTTCAAAGTCTCACCCAACTTCGCTATCTAAACCTCTCTAGCACGTCCC |
| TLR9 | XM_013116942.1 | 1405-1504 | AAGAACTTCATGGTCAGGTGTAGGAGCCTCAATTTTACTTTGGATCTGTCTCGGAATAACCTGGTGGCAATTCAGCCAGAGATGTTTGCCAACCTCTCTC |
| TMEM173 | XM_005065271.2 | 820-919 | ACCCCAACATTCGCTTCCGAGACATGCTGCCCCAGCAAAACATGAACCGTGCTGGCATCAAGAATCGGGTTTATTCCAACAGTGTCTATGAACTTCTGGA |
| TNF | XM_005086799.2 | 223-322 | CATCGGCTCCATGGCCCAGACACTCACACTCAGATCATCTTCTCAAAATTCGAACGACAAGCCTGTGGCCCACGTTGTAGCAAACCACCAAGTGGAGGAG |
| TNFAIP3 | XM_005065840.2 | 1915-2014 | TTTGGGACTCCCGAAAACAAGGGCTTCTGCACGCTGTGTTTCATCGAATACAGAGAAAATAAACAGTTTGTCACTGCGTCTGGGAAAGCGGGTTCCGTGG |
| TNFAIP6 | XM_013112595.1 | 660-759 | TCTTCTACCTACACTTTTTCTTGGCAGGCCCATGTGTTGCAAGAGCGCTGCACTTGGGAGTTTACAAACTTGGTCCGCAGGAGCACGGGATTGAGTCAGG |
| TNFRSF11A | XM_005081463.2 | 767-866 | TAAATGGAAATAAGGAGTCCTCAGGTGACTGTTGTATCGGTGCCCACTCAACAACGTCCAGTCAACGAGAAGTGTGTGAAGGTGTCTTCCTAATGACTCT |
| TNFRSF13B | XM_013111604.1 | 365-464 | AACTGGCCCTCGTCTACTGCACCCTCGGGCTCTGCCTCTGTGCCATATTCTGCTGTTTTTTGGTGGCCTTGGCCTGCTTCCTCCGGCGTAAAGAGCCGCT |
| TNFRSF13C | XM_013111140.1 | 67-166 | CCGGGACAGCCCGATGCCCACGCCTTGCGTTCAGACACTGTGCTTCGACCCTCTGGTGCGACACTGCGTGGCCTGTAACCTCCTCCGCACGCCAGACCCT |
| TNFRSF14 | XM_005079370.2 | 711-810 | TCACCTGCTCCTTCTCCGGGCAATTCTACACCATTTTGTCCATCCCTTTGGTTTTGGCTGTCCTAACAGCCTGCATCTACTTTGGAAGGAAACTACTGCA |
| TNFRSF1A | XM_005084089.2 | 774-873 | GAGTCAGGACACCGTGTGTAACTGCCACAAAGGATTCTTCCGAAGTGGAAATGAGTGTGTCTCTTGCAGTCGCTGCAAAGGAAATCACAACTGTTTCCAG |
| TNFRSF1B | XM_005079349.2 | 1165-1264 | CTCCAGGAGTTCAGATTCCTCCCACGGCAGCCATGGGACCCATGTCAATGTCACCTGCATTGTGAATGTCTGCAGTAGCCCTGACCACAGCTCACAGTGC |
| TNFRSF4 | XM_005079218.2 | 708-807 | CTGTCCAGTCCACCACAGTCTGGCCCAGGACTTCTCAGTTGCCTTCTACACCCACCTTGGTGGCTCCTAGGGGCCCTGCATTTTCCGGTTTCCTAGGCCT |
| TNFRSF8 | XM_013119976.1 | 405-504 | AGGCACAGCAGAGAAGGACACCGTCTGTGAATTGCCTTCATCAGGACCTGTCCGTGATTGTTCCAATCCAGATGACTGCAAGACACTTACTAGCCATGCC |
| TNFRSF9 | XM_005079387.2 | 388-487 | CAAAGTGTGCCTGGTGTGAAAAGGATTGCAAGCCGGGTCAAGAGTTAACAGAGCAGGGTTGTAAAAACTGTGGCTTTGGGACATTTAACGATAAGAACGG |
| TNFSF10 | XM_005069757.2 | 476-575 | CTAGGAAAGGGCATTCGTTCCTCAACTATGTGCTTTTGAGAAATGGAGAGCTGGTCATCCAGGAACAGGGCCTTTACTACATCTATTCCCAGACGTACTT |
| TNFSF11 | XM_005070940.1 | 370-469 | TTTCACTGGGCTGTGCAAAGGGAATTACAACATATTGTCGGGCCACAGCGCTTCACGGGAGTCCCAGCTATGATGGAAGGTTCATGGTCCGAACTGACCC |
| TNFSF12 | XM_013111679.1 | 541-640 | GTCAGGGCTGGGCTCTACTACCTTTACTGTCAGGTGCACTTTGATGAGGGGAAGGCTGTCTATCTGAAGCTGGACTTGCTGGTGAATGATGTGCTGGCCT |
| TNFSF13B | XM_005077356.2 | 574-673 | CCTTGGAGGAAAAAGAGAATAAAATCGTGGTGAAGCAAACGGGTTACTTCTTCATCTACAGCCAGGTTCTATACACAGATACCATCTTTGCCATGGGGCA |
| TNFSF14 | XM_005081346.2 | 134-233 | TGGGCGTGGGGCTGGCTACTCAGGGCTGGTTCCTACTGAGACTGCATCAGCGTGTTGGGGACACAGTAGCTCGCCTCCCGGAGAGAGACACGGGCTCCTG |
| TNFSF15 | XM_005075109.2 | 393-492 | ATGAACTACACCAACAAATTCCTGGTGATCCCAGAGTCGGGAGACTATTTCATTTACTCCCAGATCACATTCCGAGGGCTCAAATCCAGGTGTGGCGACA |
| TNFSF4 | XM_005071338.2 | 313-412 | ATGAAGGGCTCCTTTTTCCAGGAGGTCAAGATCAACCTTCACTTCCGAAAGAGTCGAAGTCCCATCTCTATGCCCATGCTGAACAATGGTCAAAGGGTCG |
| TNFSF8 | XM_005075108.2 | 281-380 | CCTTGAAAAGGACTCCATTCAAGAAGTCATGGGCCTACCTCCAAGTGTCAAAACATCTCAATAATACCAAACTGTCATGGAACCCAGACGGCGTCATCCA |
| TNK2 | XM_005071551.2 | 421-520 | TGTTGGAGCTGCTGTCCGAGGTGCAGCTTCAGCAGTATTTCCTGAGGCTTCGCGATGACCTCAACGTTACCCGTCTGTCCCACTTTGAGTATGTCAAGAA |
| TOLLIP | XM_005064192.2 | 288-387 | AGGCAAAATTGGCAAAGAATTATGGCATGACTCGCATGGACCCTTATTGTCGTCTGCGTCTGGGCTATGCTGTTTATGAAACTCCCACAGCACATAATGG |
| TP53 | NM_001281661.1 | 1091-1190 | CCCAGCCAAAGAGAAAAACACTTGACGGAGAATATTTCACCCTTAAGATCCGTGGTCAAGAACGCTTCAAGATGTTCCAAGAATTGAATGAGGCCTTGGA |
| TPA | XM_013110872.1 | 1535-1634 | GAGGAATCATCTCCTTTCTTCTCTGATCGGCTGAAGGAAGCCCACGTGAGGCTGTACCCATCGAGTCGCTGTACTTCACAGCATTTGTCTAACAAAACCG |
| TPT1 | XM_005070954.2 | 517-616 | GTTTTTTATTGGTGAGAACATGAATCCAGATGGCATGGTGGCTCTCCTGGACTACCGTGAAGATGGTGTGACTCCTTTCATGATTTTCTTTAAGGACGGC |
| TRADD | XM_005076194.2 | 719-818 | CTTTTCTGTTTCGTGGGCAGCCCGTAGTGAACCGGCCATTGAATCTGCAAGACCAGCAGACATTCGCGCGCTCAGTGGGCCTCAAGTGGCGCAGGGTGGG |
| TRAF1 | XM_005082079.1 | 836-935 | GTCACGAGTCAGTGTGTGGCAGGACTGTCAGCCTCTTCTCTCCAGCTTTCTACACGGCCAAGTATGGTTATAAGTTGTGTCTGCGGTTGTACCTGAATGG |
| TRAF2 | XM_005083680.2 | 649-748 | AGAAGAAAATCTCTCGGGAGAAGTTTCAGGACCATGTTAGATCATGCAGCAAGTGTCGGGTTCCGTGCAGATTCCATACCATCGGCTGTTCTGAGATGGT |
| TRAF3 | XM_005068328.2 | 1416-1515 | TTTCTACACGGGCTATTTTGGCTATAAGATGTGTGCCAGGGTCTACCTGAACGGGGACGGAATGGGAAAAGGGACACACTTGTCGCTGTTTTTTGTCATT |
| TRAF4 | XM_005077031.2 | 1059-1158 | CAAGGCCAAGCCTAACCTGGAGTGCTTCAGTCCAGCCTTCTATACACATAAGTACGGCTACAAGCTGCAGGTGTCTGCATTCCTCAATGGTAACGGCAGT |
| TRAF5 | XM_005082861.2 | 951-1050 | CAAGTGTCTGAGGACTATTCCGAGAACTGAGGTGAATGAACACCTTACCGTGTGTCCTGAGGCCGAACAAGACTGTCCCTTTCAGCACTACGGCTGTACT |
| TRAF6 | XM_013118156.1 | 1104-1203 | GTTTTTGGCTGTCATGAGAAGATGCAGAGAAATCACCTAGCACGACACCTGCAAGAGAATACTCAGTTGCACATGAGATTGTTGGCCCAGGCTGTTCATA |
| TRAP1 | XM_013121731.1 | 1620-1719 | TACTCTTCTGCTACGAGCAGTTTGATGAGCTTACTTTGCTGCACCTGCGGGAGTTCGACAAGAAGAAGCTGATCTCTGTGGAGACAGACATCGTCGTTGA |
| TREM1 | XM_005072398.2 | 128-227 | AGTACACACTGAAAGATGACCCTGATGAGGCCATGTTACATGTCCAAATGGATGACCTTCGAGTGACAGACTCTGGATTGTACCGCTGTGTCATTTACCG |
| TREM2 | XM_005072468.2 | 496-595 | TGGTGGAGGTGCTGGCAGACCCTCTAGATGAACAAGATGCCGAAGACCTATGGGTCCCTAAGGAATTGGAGAGTTTTGATGGTGCCCAAGTGGAACACAG |
| TSAD | XM_013120569.1 | 852-951 | CCCTGCCTCACGACGCCATCGAGCTCTGCCCACTAACGCCATCTACCAGGAACCTGATGAACCCATAGCCTTCTACGCCATGGGACGGGGCAGCCCCGGG |
| TSLP | XM_013123196.1 | 584-683 | GGGAAGACCCTCCAGCCGTTCTGGCGTTTTCTTTGCTCCCGGAACTCTGTGAGCAGACAGATTGTCTCATGAAAATCGAGCACCTTACTCTCAATCCTGT |
| TWIST2 | XM_013122113.1 | 139-238 | CCGGGCAAGCGCGGCAAGAAGGGCAGTCCGAGCGCGCAGTCCTTCGAGGAGTTGCAGAGCCAGCGCATCCTGGCCAACGTGCGCGAGCGCCAGCGCACCC |
| TYK2 | XM_005078559.2 | 3590-3689 | CCCGGAATGCCTGAAGGAGTGCAAATTTTACTATGCATCAGATGTCTGGTCCTTCGGGGTGACCTTGTATGAGCTGTTGACGTACTGTGACTCTGGACAG |
| TYMP | XM_013111290.1 | 658-757 | TCCCTGCTGATGGAATCCTGTATGCTGCACGAGATGTGACAGCTACTGTGGACAGTGTGCCACTCATTACAGCCTCAATCCTCAGTAAGAAGGTCGTGGA |
| TYROBP | XM_005081821.2 | 118-217 | AGGATTAAGTCCAGTCCAGGCCCAGAACGAATGCAACTGCTCTCCCGTGAGCCCAGGCGTACTGGCTGGGATTGTGCTTGGTGACTTGATGCTGACATTG |
| UBE2L3 | XM_005077563.2 | 121-220 | TTTATTGACTTGGCAAGGGCTTATTGTTCCTGACAACCCTCCATACGACAAGGGGGCCTTCAGAATTGAAATCAACTTCCCAGCAGAGTATCCATTCAAA |
| VCAM1 | XM_005077100.2 | 1897-1996 | CTGCTCAAGCGATGGTTTTCCAGCTCCAAAAATCCTGTGGAGCAGACAACTAAATAATGGGGAAATGCAGCCTCTTTCCGAAGATACAGTGCTCACCTTA |
| VCAN | XM_005065515.2 | 3663-3762 | AATTTGCATCAACTTTGAGGCCCTTCAGAGCTCAAGTCACACAGCTTATGGAGGAAACTACTGAAGAACGAAAAAAGTCATCCCTAGACTACACAGATTT |
| VTN | XM_005077006.2 | 1475-1574 | AGCAGCAGCCACAGTTTGAACTCTCGTCGCTCATCGCGTTCAGTCTGGTTCTCTTTGTTCTCTAGTGAGGAGAGCGGGCTAGGAGACTACAACTATGATT |
| XAF1 | XM_005067820.2 | 711-810 | CGAGGGCTGCTCTTCCTAAAGGAGATGAAACAGCCTATGACATCTTTCGGAAGTGCTGCTGGTGCAGCATCTTACTTCCCTTGCCTATTCTAAATCAGCA |
| XBP1 | XM_005087805.2 | 37-136 | AGGCCTTCAAGGCCCACGGGCAGAAAAGCTGTGGTTTTTGTCTTTTTGAGAGGAGGAGCCTCAGAATGTATTTACCACTGTTTAGCCTTATTCTGGAAAG |
| XCL1 | XM_005071377.2 | 850-949 | CACTGAAGTCCTAGAAGAGAGTTTCTGTGTGAGCTTAACAGCACAACAGTTGCCGGTTCAAAAAATCAAGACCTATACCATCAGGGAGGGCATATTGAAA |
| XCR1 | XM_005082283.1 | 486-585 | TGTGTGGACAGCCAGCATCCTGTCCTCTATTCCCGATGCTATCTTCCACAAGGTGAATGTCTCAGCATGTGATTATTCTGAACTTCGTGGGTTCTTGGCC |
| ZAP70 | XM_013117333.1 | 658-757 | AAGGAGCAGGGAACATACGCGCTGTCCCTGATCTATGGGAAAACTGTGTATCACTATCTCATCAGCCAGGACAAGGCTGGCAAGTTCTACATTCCCGAAG |
| ZBTB16 | XM_005069286.2 | 778-877 | TTCGGTCTCCACCTCTTTTGGACTTTCAGCCATGAGTCCTACCAAGGCAGCAGTGGACAGTTTGATGACCATAGGGCAGTCTCTCCTGCAAGGAACCCTT |
| ZEB1 | NM_001281620.1 | 2231-2330 | CAGCTGTGTTACAGACTCAGAACCAGTTGTAAATGTAATCCCACCAAGTGCCAACCCCATAAATATTGCTATTCCTACAGTCACTGCCCAGTTACCCACC |
| ZNF205 | XM_005081745.2 | 907-1006 | GGGGAAGGCTGGACCACACACAGCAAAATTTCTACAGGGATGTACTACAGGAGGAAAATGGGCTGGCTTTGGGGTTCCCGTGCAGCAGACCGTTCAGGGT |
